# A brain-tumor neural circuit controls breast cancer progression in mice

**DOI:** 10.1172/JCI167725

**Published:** 2023-10-17

**Authors:** Si-Yi Xiong, Hui-Zhong Wen, Li-Meng Dai, Yun-Xiao Lou, Zhao-Qun Wang, Yi-Lun Yi, Xiao-Jing Yan, Ya-Ran Wu, Wei Sun, Peng-Hui Chen, Si-Zhe Yang, Xiao-Wei Qi, Yi Zhang, Guang-Yan Wu

**Affiliations:** 1 Breast and Thyroid Surgery, Southwest Hospital,; 2 Department of Neurobiology, Chongqing Key Laboratory of Neurobiology, College of Basic Medical Sciences,; 3 Department of Medical Genetics, College of Basic Medical Sciences,; 4 Experimental Center of Basic Medicine, Chongqing Key Laboratory of Neurobiology, College of Basic Medical Sciences,; 5 Department of Biochemistry and Molecular Biology, College of Basic Medical Sciences,; 6 Department of Clinical Biochemistry, Faculty of Pharmacy and Laboratory Medicine, and; 7 Biomedical Analysis Center, Army Medical University, Chongqing, China.

**Keywords:** Neuroscience, Oncology, Breast cancer

## Abstract

Tumor burden, considered a common chronic stressor, can cause widespread anxiety. Evidence suggests that cancer-induced anxiety can promote tumor progression, but the underlying neural mechanism remains unclear. Here, we used neuroscience and cancer tools to investigate how the brain contributes to tumor progression via nerve-tumor crosstalk in a mouse model of breast cancer. We show that tumor-bearing mice exhibited significant anxiety-like behaviors and that corticotropin-releasing hormone (CRH) neurons in the central medial amygdala (CeM) were activated. Moreover, we detected newly formed sympathetic nerves in tumors, which established a polysynaptic connection to the brain. Pharmacogenetic or optogenetic inhibition of CeM^CRH^ neurons and the CeM^CRH^→lateral paragigantocellular nucleus (LPGi) circuit significantly alleviated anxiety-like behaviors and slowed tumor growth. Conversely, artificial activation of CeM^CRH^ neurons and the CeM^CRH^→LPGi circuit increased anxiety and tumor growth. Importantly, we found alprazolam, an antianxiety drug, to be a promising agent for slowing tumor progression. Furthermore, we show that manipulation of the CeM^CRH^→LPGi circuit directly regulated the activity of the intratumoral sympathetic nerves and peripheral nerve–derived norepinephrine, which affected tumor progression by modulating antitumor immunity. Together, these findings reveal a brain-tumor neural circuit that contributes to breast cancer progression and provide therapeutic insights for breast cancer.

## Graphical abstract

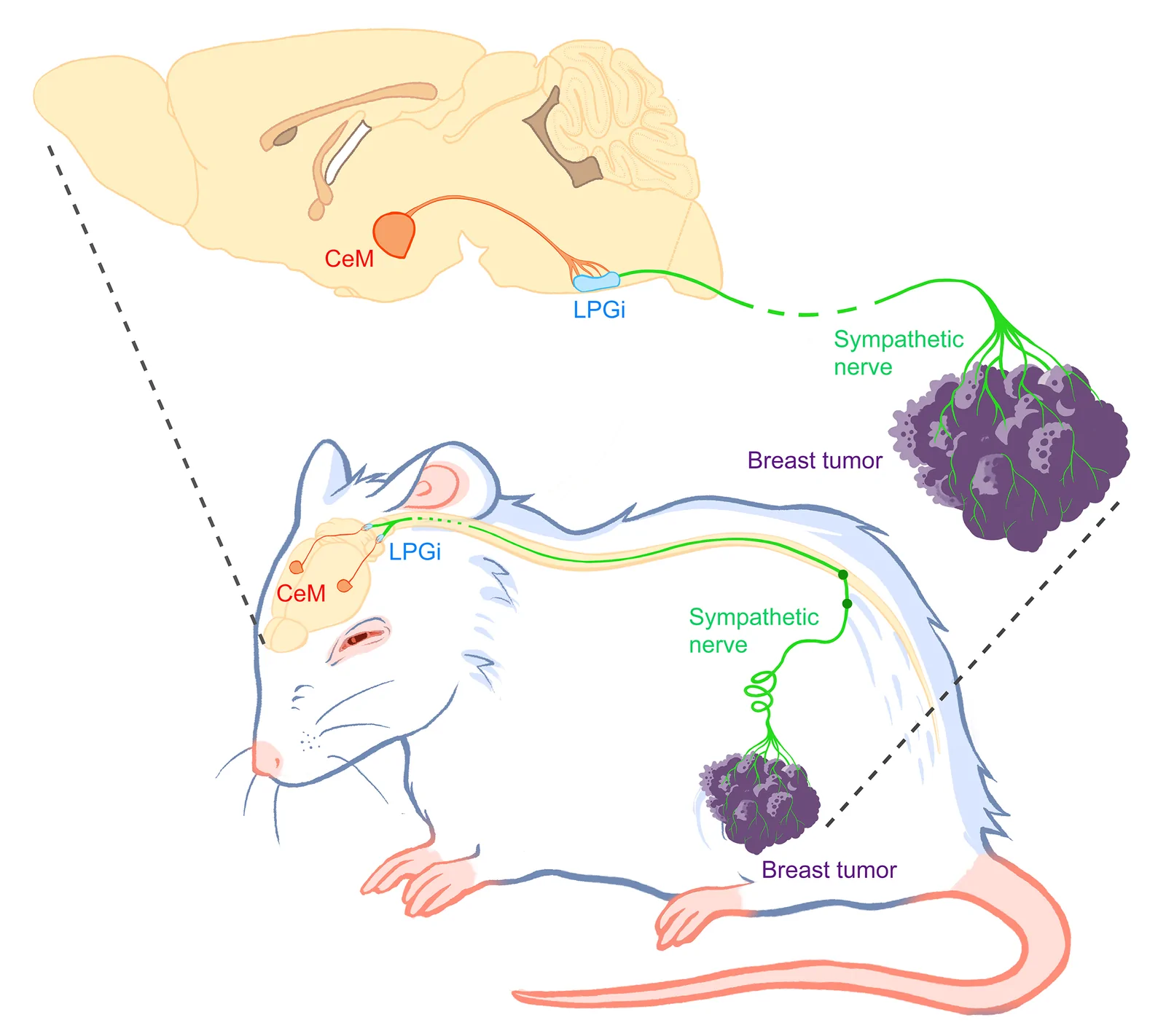

## Introduction

Tumor burden is perceived as a chronic stressor that can induce widespread anxiety (1–5). A growing body of evidence suggests that cancer-induced anxiety can accelerate tumor progression (6–10). Clinical data have demonstrated that psychotherapy and medication treatment can slow cancer progression by reducing anxiety (11–13). However, how brain neural activity underlying anxiety promotes tumor progression remains unclear.

The nervous system is widely distributed throughout the body and can regulate the development of organs and maintain homeostasis through peripheral nerves, which are controlled by the brain. Accumulating evidence indicates that the nervous system plays an important role in cancer pathogenesis (14–16). Neuronal synaptic communication with brain cancer cells can regulate the growth of malignant gliomas through neurotransmitter- and voltage-regulated mechanisms (17). Mounting evidence indicates that there is also a direct connection between peripheral nerves and tumors (15, 18, 19). For example, a study revealed that surgical or pharmacological denervation of the stomach can markedly reduce tumor progression (19). The sympathetic nervous system is an important pathway by which stress can promote tumor growth (20). Notably, tumor-specific sympathetic denervation suppresses prostate cancer and breast cancer progression in mouse models (21, 22). Nerve-cancer crosstalk can occur directly or through nervous system control of other cell types in the tumor microenvironment, such as immune cells and stromal cells (15, 18, 19). The sympathetic nervous system neurotransmitter norepinephrine (NE) may activate α- and β-adrenergic receptors of the tumor cells or other cells in the tumor microenvironment (20). It is known that anxiety could affect the activity of the sympathetic nervous system (23, 24), but it remains unclear how brain neural activities underlying anxiety control tumor progression.

The neural circuits of the amygdala and connected brain regions are thought to be essential for anxiety processing (25–27). Patients with cancer exhibit a higher incidence of anxiety disorder, which is characterized by hyperactivity of the amygdala (28, 29). The central medial amygdala (CeM) is the main output nucleus of the amygdala, which integrates cortical and intra-amygdala afferents and regulates anxiety by projecting to the brainstem nuclei including the lateral paragigantocellular nucleus (LPGi). Moreover, corticotropin-releasing hormone CeM (CeM^CRH^) neurons orchestrate physiological and behavioral responses to anxiety (30, 31). Previous studies suggested that LPGi catecholaminergic (LPGi^CA^) neurons innervate sympathoadrenal preganglionic neurons and are involved in regulating autonomic responses to various stressors (32, 33). Given that there are abundant nerves in tumors and that they are ultimately connected to the brain (21, 22), we explored whether brain CeM^CRH^ neurons and associated circuitry control anxiety-promoting tumor progression via direct neural connections.

In this study, we injected rAAV2/2-CRH-Cre together with rAAV2/9-EF1α-Flex-taCasp3-TEVp into the CeM to ablate the CRH neurons and used optogenetic and chemogenetic approaches to specifically activate or inhibit CeM^CRH^ neurons to investigate their role in cancer-induced anxiety and tumor progression. We demonstrate, using a transplanted (4T1) breast cancer model, that ablation or inhibition of CeM^CRH^ neurons and the CeM^CRH^→LPGi circuit markedly decreased anxiety-like behaviors and tumor growth. This manipulation led to an obvious reduction in intratumoral sympathetic activity, as evidenced by diminished local NE levels in tumors. Consistently, activation of CeM^CRH^ neurons or their projection to LPGi significantly promoted anxiety-like behavior and tumor progression. Alprazolam was found to slow down tumor progression by inhibiting CeM^CRH^ and LPGi^CA^ neurons. The peripheral nerve–derived NE thus affecting tumor progression was mediated by immune system functions. Consistently, the above results were validated in a spontaneous mouse MMTV-PyMT mouse model of breast cancer. Therefore, our research reveals a brain-tumor neural circuit that contributes to breast cancer progression.

## Results

### Tumor-bearing mice exhibit severe cancer-induced anxiety.

**Figure 1:**
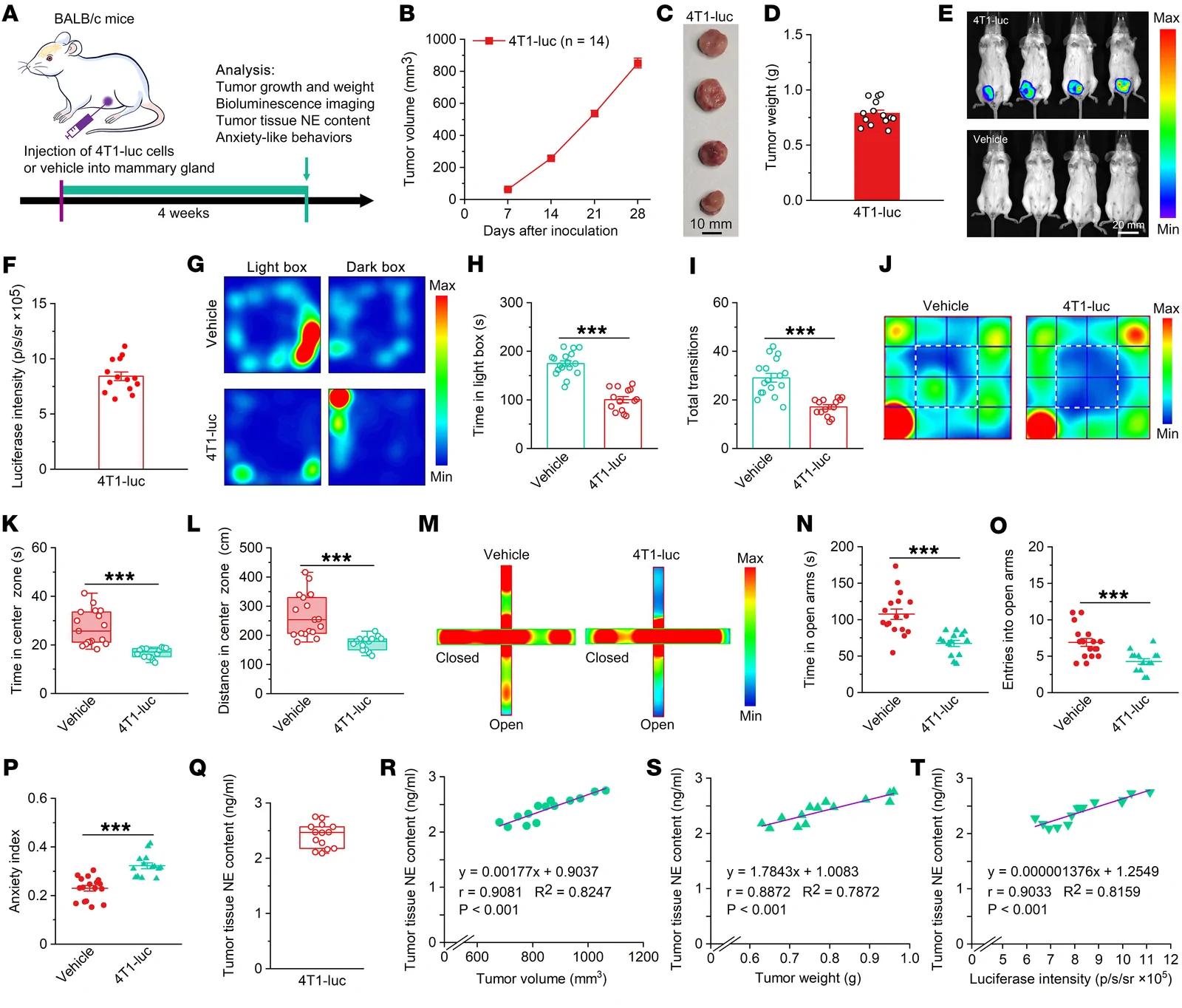
4T1 tumor–bearing mice show obvious anxiety. (**A**) Schematic of the experimental design. (**B**) Tumor growth of mice injected with 4T1-luc cancer cells (*n* = 14). (**C**) Representative tumors dissected from 4T1 tumor–bearing mice. (**D**) Tumor weights 4 weeks after inoculation with 4T1-luc cancer cells (*n* = 14). (**E**) Representative bioluminescence images of mice of the 2 groups. Scale bar: 20 mm. (**F**) The luciferase intensity of tumor 4 weeks after inoculation with 4T1-luc cells (*n* = 14). (**G**–**I**) LDT: representative heatmaps (**G**) and quantitative summary of the time spent in the light box (**H**) and the total number of transitions (**I**) in the vehicle (*n* = 17) and 4T1-luc (*n* = 14) treatment groups. (**J**–**L**) OFT: representative heatmaps (**J**) and quantification of the time spent in the center zone (**K**) and the distance traveled in the center zone (**L**) in the vehicle (*n* = 17) and 4T1-luc (*n* = 14) treatment groups. (**M**–**P**) EPM test: representative heatmaps (**M**) and quantification of the time spent in the open arms (**N**), entries into the open arms (**O**), and the anxiety index (**P**) in the vehicle (*n* = 17) and 4T1-luc (*n* = 14) treatment groups. (**Q**) NE content of tumor tissue 4 weeks after 4T1-luc cell inoculation (*n* = 14). (**R**–**T**) Correlation between tumor volume and tumor tissue NE content (**R**), tumor weight and tumor tissue NE content (**S**), and tumor luciferase intensity and tumor tissue NE content (**T**). Data are presented as the mean ± SEM, except in the box plot (**K**, **L**, and **Q**), in which the centerline indicates the median, box edges represent the first and third quartiles, and whiskers denote minimal and maximal values. ***P < 0.001, by 2-tailed, unpaired Student’s *t* test (**H**, **I**, **K**, **L**, and **N**–**P**) and 2-sided linear regression analysis (**R**–**T**). Max, maximum; Min, minimum.

Accumulated clinical and experimental evidence has clearly demonstrated that tumor burden, considered an important chronic stressor, can induce widespread negative emotions, such as anxiety (34, 35). To investigate the relationship between breast cancer and anxiety-like behaviors in rodents, we first injected 4T1 cancer cells into the mammary glands of BALB/c mice to develop a mouse model of orthotopic breast cancer. After 28 days of tumor development (Figure 1, A–F), the 4T1 tumor–bearing mice exhibited multiple anxiety-like behaviors in routine assays, including the light-dark box test (LDT) (reduced light box time and total transitions; Figure 1, G–I), the open-field test (OFT) (reduced time and distance in the center zone; Figure 1, J–L), and the elevated plus maze (EPM) test (decreased time and entries in open arms and increase in the anxiety index) (Figure 1, M–P) compared with control mice. Notably, 2-sided linear regression analysis illustrated that the degree of cancer-induced anxiety was strikingly correlated with the volume, weight, and luciferase intensity of the 4T1 tumors (all *P* < 0.001, *R^2^* ≥ 0.6506; Supplemental Figure 1; supplemental material available online with this article; https://doi.org/10.1172/JCI167725DS1) . Moreover, 7 days after 4T1-luc cancer cell injection, the 4T1 tumor–bearing mice also displayed severe anxiety-like behaviors at the early tumor stage (Supplemental Figure 2). In addition, we also assessed the anxiety-like behaviors of the PyMT tumor–bearing mice in a spontaneous breast cancer model. As expected, we obtained similar results in PyMT tumor–bearing mice. Behavioral results revealed that PyMT tumor–bearing mice had obvious anxiety-like behaviors at 13 weeks of age (Supplemental Figure 3). Together, these results consistently suggest that anxiety-like behaviors were reliably induced by these breast cancer models and that there was frequent and common anxiety in the breast tumor–bearing mice.

In addition, numerous studies have shown that the breast tumors are innervated by sympathetic nerves (22, 36) and that the activity of the sympathetic nervous system is commonly associated with anxiety levels (37, 38). Therefore, we next assessed the relationship between intratumor sympathetic activity and tumor progression. The content of the sympathetic nervous system neurotransmitters (i.e., NE) in peripheral tissues is an indicator of sympathetic activation (39). We determined the NE concentration by ELISA. Analysis of the tumor tissue NE concentration revealed that the NE concentration in the 4T1 tumor tissues was highly associated with the volume, weight, and luciferase intensity of the tumors. The high positive correlations were confirmed by 2-sided linear regression analysis (all *P* < 0.001, *R^2^* ≥ 0.7872; Figure 1, Q–T). These results indicate that intratumoral sympathetic activity was positively correlated with breast cancer progression.

### A neural circuit connects the brain with breast tumors.

**Figure 2:**
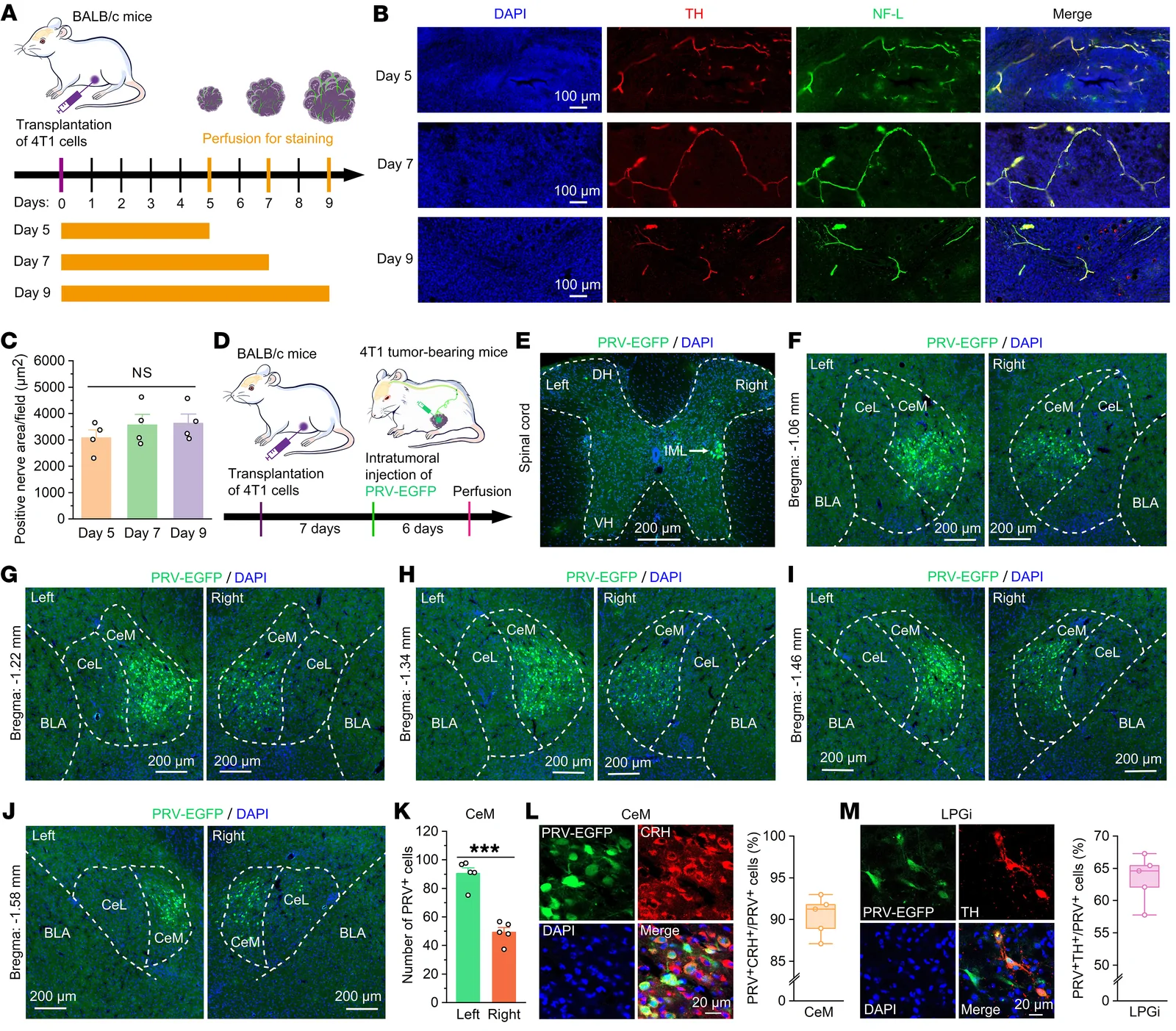
Newly formed sympathetic innervation of 4T1 tumors connects to the brain. (**A**) Schematic diagram of immunofluorescence staining for TH and NF-L in breast tumors (*n* = 4 for each group). (**B**) Representative images showing immunofluorescence staining for TH and NF-L at days 5, 7, and 9 after 4T1 cell inoculation. Scale bars: 100 μm. (**C**) Quantification of TH^+^ sympathetic nerve fibers in outer regions of the tumor (field surface = 0.15 mm^2^; *n* = 4 for each group). (**D**) Experimental scheme showing the transplantation of 4T1 cells and intratumoral injection of the neurotropic retrograde transpolysynaptic pseudorabies virus PRV-EGFP. (**E**–**J**) Representative images showing PRV-infected neurons (green) in the intermediolateral cell column (IML) (**E**) and CeM (**F**–**J**) from the mice 6 days after PRV-EGFP injection into the tumor tissue. Scale bars: 200 μm. (**K**) Quantification of PRV^+^ neurons in the left and right CeM (*n* = 5). (**L**) Representative images and quantification of PRV^+^CRH^+^ neurons among PRV^+^ neurons in the CeM (*n* = 5). (**M**) Representative images and quantification of PRV^+^TH^+^ neurons among PRV^+^ neurons in the LPGi (*n* = 5). Scale bars: 20 μm (**L** and **M**). Data are presented as the mean ± SEM, except in box and half violin plots (**L** and **M**), in which center lines indicate the median, box edges represent the first and third quartiles, and whiskers denote minimal and maximal values. ***P < 0.001, by 1-way ANOVA followed by Tukey post hoc test (**C**) and 2-tailed, unpaired Student’s *t* test (**K**). BLA, basolateral amygdaloid nucleus; CeL, central nucleus of the amygdala, lateral division; DH, dorsal horn; VH, ventral horn.

A large body of evidence suggests that many brain areas are activated during anxiety (40–42). Importantly, anxiety-induced hyperactivation of some brain regions can cause overactivity of the sympathetic nervous system (43, 44). A recent study has suggested that the activity of local sympathetic innervation in breast tumors makes a significant contribution to tumor growth and progression (22). However, it remains unknown whether the breast tumor is innervated by newly formed sympathetic nerves in the initial phases of cancer development. To address this situation, we injected 4T1 cancer cells into the mammary glands of BALB/c mice to develop a mouse model of orthotopic breast cancer. Immunofluorescence staining was then performed 5, 7, and 9 days after tumor cell inoculation, respectively (Figure 2A). Double immunofluorescence staining with the newly formed neuron-specific marker neurofilament-L (NF-L) and the sympathetic nerve marker tyrosine hydroxylase (TH) revealed that 4T1 mammary tumors displayed denser sympathetic innervation 5, 7, and 9 days after tumor development (Figure 2, B and C, and Supplemental Figure 4). Additionally, in spontaneous MMTV-PyMT tumors, we detected TH^+^ sympathetic innervation in tumors of mice at 10 weeks of age (Supplemental Figure 7A). Therefore, these results suggest that the breast tumor recruits newly formed sympathetic nerve fibers distributed in the tumor stroma at the early carcinoma stage.

Next, to investigate whether the newly formed sympathetic nerves connect to higher-order circuits, we performed retrograde transpolysynaptic tracing using fluorescent protein–producing pseudorabies virus (PRV). Six days after injection of PRV-CAG-EGFP into the 4T1 breast tumor stroma (Figure 2D), PRV ascended from the 4T1 tumor up into the spinal cord, brain stem, hypothalamus, and forebrain (Figure 2, E–J, and Supplemental Figure 5), which was similar to the injection of PRV into the mammary gland of WT BALB/c mice (only showing the CeM; Supplemental Figure 6, B and C). The mammary gland contained a dense network of local sympathetic fibers (Supplemental Figure 6A). Moreover, this observation is also similar to previous findings with injection of PRV directly into the stellate ganglion and adrenal gland of rats (45). Additionally, in the MMTV-PyMT mouse model, we also observed PRV-infected neurons in these regions (only showing the CeM), with injection of the same PRV into the PyMT tumor (Supplemental Figure 7, B and C). Notably, the CeM and lateral paragigantocellular nucleus (LPGi) were prominently infected by PRV (Figure 2, F–K, and Supplemental Figure 5G). Next, to determine the neurotransmitter type of PRV-infected CeM and LPGi neurons, we performed immunofluorescence staining. Given that CeM^CRH^ neurons play a critical role in the modulation of anxiety and that LPGi^CA^ neurons are involved in regulating autonomic responses to various stressors (30, 46, 47), we chose to focus on exploring whether PRV-infected CeM and LPGi neurons express CRH and TH, respectively. Immunofluorescence results indicated that PRV-infected CeM neurons were predominantly colocalized with a CRH-specific antibody (Figure 2L) and that the majority of PRV-infected LPGi neurons expressed TH (Figure 2M). Taken together, these results reveal that the newly formed sympathetic innervation of the breast tumor polysynaptically connected to CeM^CRH^ neurons and LPGi^CA^ neurons.

### CeM^CRH^ neurons are activated in breast cancer.

**Figure 3:**
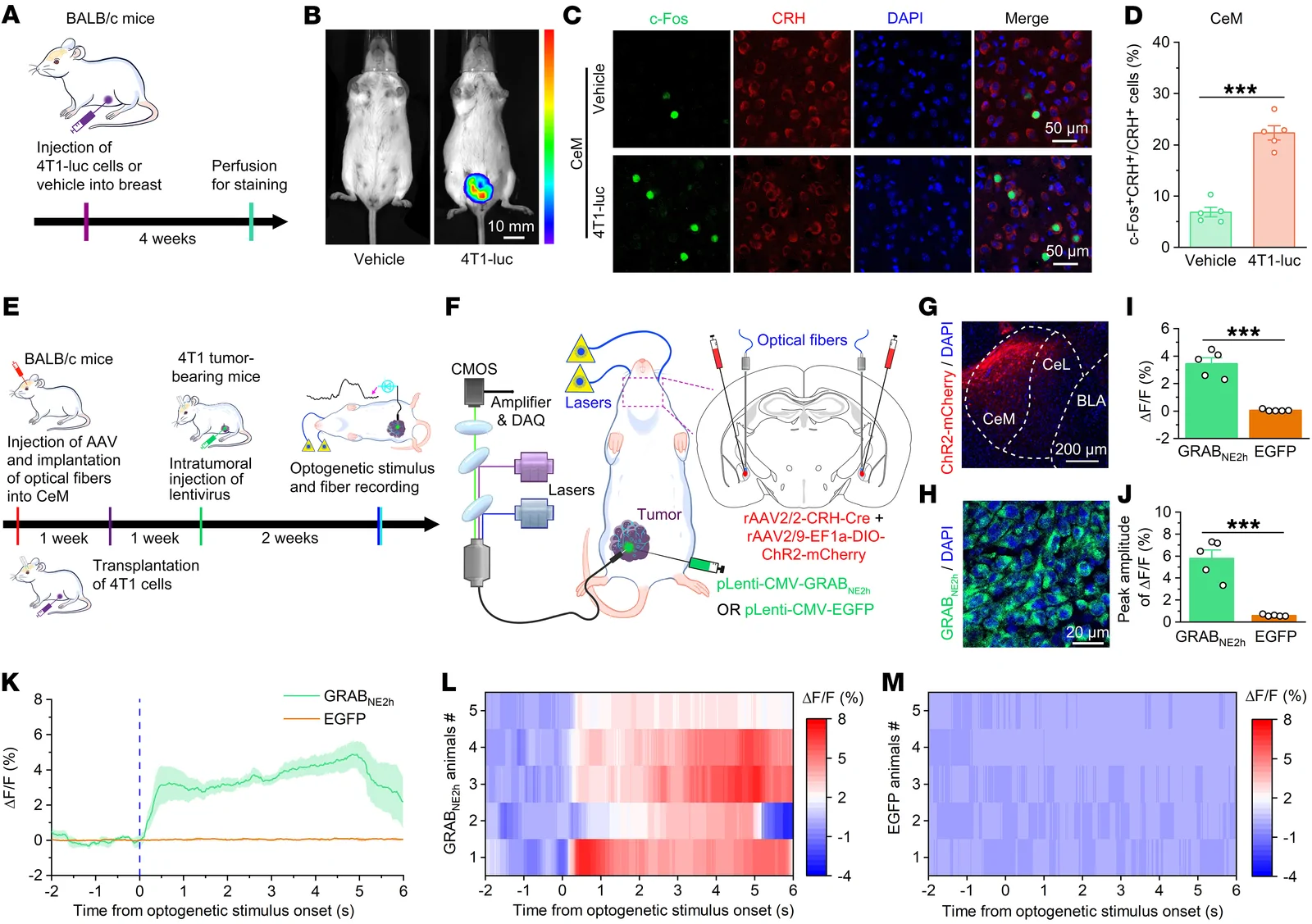
Activation of CeM^CRH^ neurons increases the activities of local sympathetic nerves distributed in mammary tumors. (**A**) Timeline for c-Fos and CRH immunofluorescence staining. (**B**) Representative bioluminescence images of mice 4 weeks after injection of vehicle or 4T1-luc breast cancer cells. Scale bar: 10 mm. (**C** and **D**) Representative images (**C**) and quantification (**D**) of c-Fos^+^ neurons colocalized with CeM^CRH^ neurons from the vehicle and 4T1-luc treatment groups (*n* = 5 for each group). Scale bars: 50 μm. (**E** and **F**) Timeline and scheme for recording the activities of sympathetic nerves distributed in tumor stroma during optogenetic stimulation of CeM^CRH^ neurons. (**G** and **H**) Typical image of virus expression in CeM (**G**) and tumor stroma (**H**). Scale bars: 200 μm (**G**) and 20 μm (**H**). (**I** and **J**) Comparison of the mean ΔF/F (0–5 s) (**I**) and peak amplitude of ΔF/F (**J**) between the GRAB_NE2h_ and EGFP groups (*n* = 5 for each group). (**K**) Average fluorescence change in the GRAB_NE2h_ and EGFP groups, with shaded areas indicating the SEM. (**L** and **M**) Heatmaps show the average fluorescence change in the GRAB_NE2h_ (**L**) and EGFP (**M**) groups. Data are presented as the mean ± SEM. ***P < 0.001, by 2-tailed, unpaired Student’s *t* test (**D**, **I**, and **J**).

It is well known that CeM^CRH^ neurons play a central role in mediating anxiety (30, 31). To determine whether CeM^CRH^ neurons are activated in breast tumor–bearing mice with anxiety, we assessed the expression of c-Fos (an immediate-early gene marker of neural activity) in CeM^CRH^ neurons 28 days after 4T1 tumor development (Figure 3, A and B). Consistently, double immunofluorescence staining showed that CeM^CRH^ neurons had significantly higher c-Fos expression in tumor-bearing mice than that in control mice (Figure 3, C and D), suggesting that CeM^CRH^ neurons were activated in the tumor-bearing mice.

Next, to determine the direct functional effects of activation of CeM^CRH^ neurons on the activity of sympathetic nerves distributed in the tumor stroma, we injected Cre-dependent recombinant adeno-associated virus (rAAV) expressing channelrhodopsin-2 (ChR2, light-gated cation pumps can depolarize neurons and evoke action potentials) fused with mCherry (rAAV2/9-EF1α-DIO-ChR2-mCherry) together with rAAV2/2-CRH-Cre virus into the bilateral CeM and implanted optical fibers above the bilateral CeM for optogenetic activation of these neurons. One week later, we injected 4T1 breast cancer cells into the mammary gland. Two weeks later, we injected pLenti-CMV-GRAB_NE2h_ or pLenti-CMV-EGFP (as a control) virus into the 4T1 tumor stroma of the mice (Figure 3E). After virus expression, we performed optogenetic activation of CeM^CRH^ neurons and simultaneously used fiber photometric recording of the fluorescent signals of the GPCR activation–based NE (GRAB_NE2h_) sensor (a genetically encoded NE biosensor by AAV injection) or of EGFP in anesthetized mice. Tissue NE concentration is an indicator of local sympathetic activity (39). Therefore, sympathetic nerve activity in the tumor was measured by GRAB_NE2h_ fluorescence (Figure 3, F–H). We found that optogenetic stimulation of CeM^CRH^ neurons induced a robust increase in fluorescence signals of GRAB_NE2h_ in 4T1 tumor tissue. In contrast, the fluorescence signals in control mice expressing EGFP in 4T1 tumor tissue showed no significant change during optogenetic stimulation (Figure 3, I–M). Altogether, these results suggest that CeM^CRH^ neurons were connected to and could directly activate sympathetic nerve fibers distributed in the breast tumor stroma.

### Ablation of CeM^CRH^ neurons decelerates the cancer-induced anxiety and progression of breast tumors.

**Figure 4:**
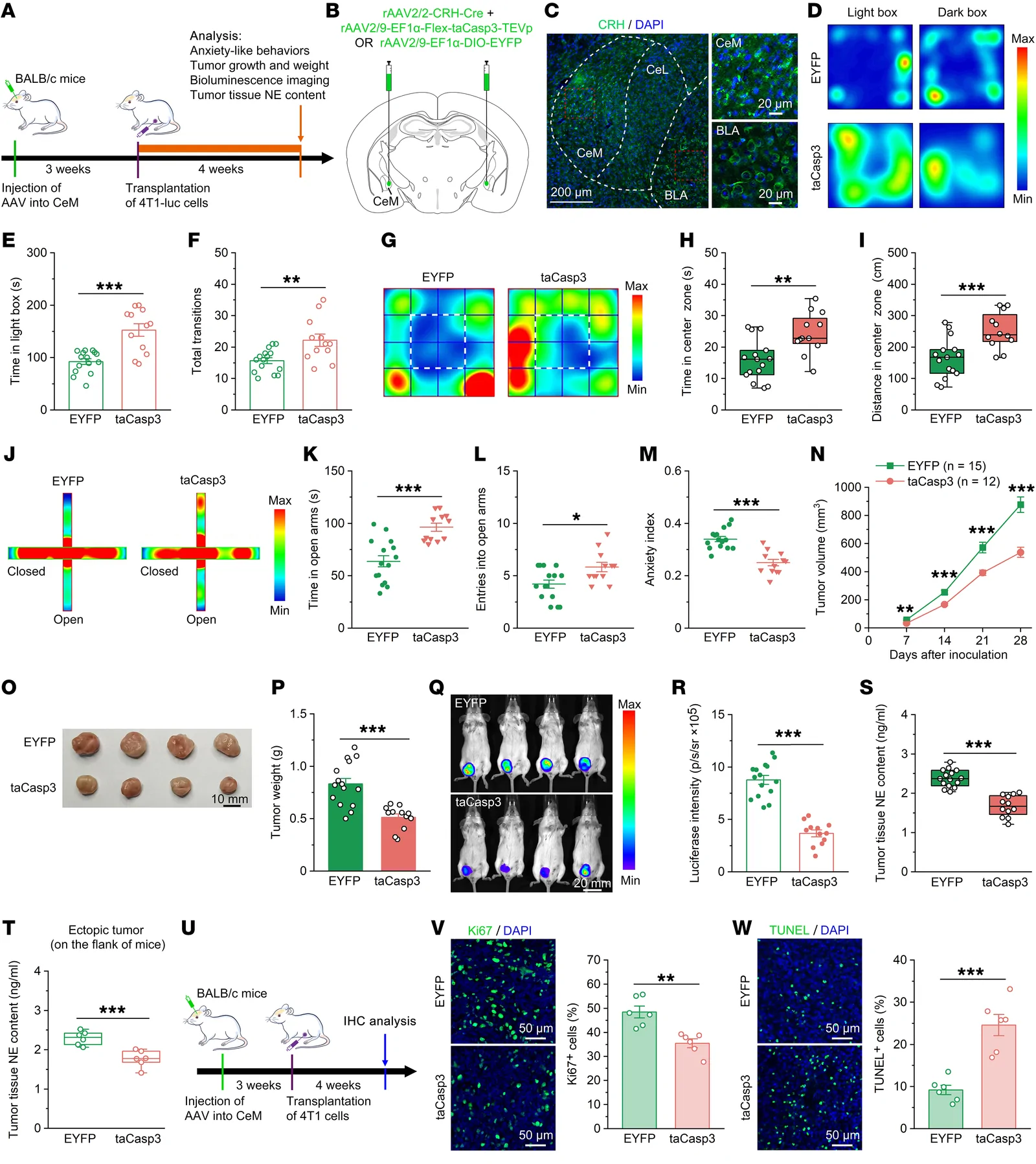
Specific ablation of CeM^CRH^ neurons significantly reduces cancer-induced anxiety and suppresses 4T1 tumor progression. (**A**) Schematic of the experimental design. (**B**) Schematic showing bilateral injection of viruses into the CeM. (**C**) Representative images showing the successful ablation of CeM^CRH^ neurons. Scale bars: 200 μm and 20 μm. (**D**–**M**) Representative heatmaps and summary data for the EYFP (*n* = 15) and taCasp3 (*n* = 12) groups in the LDT (**D**–**F**), the OFT (**G**–**I**), and the EPM test (**J**–**M**). (**N**) Ablation of CeM^CRH^ neurons significantly slowed 4T1 tumor growth. (**O**) Representative images of 4T1 tumors dissected from mice of the 2 groups. Scale bar: 10 mm. (**P**) The ablation of CeM^CRH^ neurons significantly reduced 4T1 tumor weight. (**Q**) Representative bioluminescence images of mice of the 2 groups. Scale bar: 20 mm. (**R**) The ablation of CeM^CRH^ neurons significantly reduced the luciferase intensity of 4T1 tumors (**P** and **R**: EYFP, *n* = 15, taCasp3, *n* = 12). (**S** and **T**) The ablation of CeM^CRH^ neurons significantly decreased NE content of 4T1 orthotopic mammary (**S**) and ectopic (**T**) tumors (**S**: EYFP, *n* = 15, taCasp3, *n* = 12; **T**: *n* = 6 for each group). (**U**) Timeline for immunofluorescence staining of 4T1 tumor tissues. (**V** and **W**) Representative images and quantification of Ki67^+^ cells (**V**) and TUNEL^+^ cells (**W**) within 4T1 tumors (*n* = 6 for each group). Scale bars: 50 μm. Data are presented as the mean ± SEM, except in box plots (**H**, **I**, **S**, and **T**), in which center lines indicate the median, box edges represent the first and third quartiles, and whiskers denote minimal and maximal values. *P < 0.05, **P < 0.01, and ***P < 0.001, by 2-way, repeated-measures ANOVA followed by separate 1-way ANOVA (**N**) and 2-tailed, unpaired Student’s *t* test (**E**, **F**, **H**, **I**, **K**–**M**, **P**, **R**–**T**, **V**, and **W**).

We subsequently aimed to determine whether anxiety-induced hyperactivation of CeM^CRH^ neurons influences breast cancer progression. Three weeks after the injection of rAAV2/2-CRH-Cre together with rAAV2/9-EF1α-Flex-taCasp3-TEVp (encoding the fusion protein taCasp3-TEVp, which causes host neuron apoptosis; ref. 48) or rAAV2/9-EF1α-DIO-EYFP (as a control) viruses into the bilateral CeM to ablate the CRH neurons, we inoculated 4T1-luc tumor cells into the mammary glands of BALB/c mice (Figure 4, A and B). The successful ablation of CeM^CRH^ neurons by rAAV2/2-CRH-Cre together with rAAV2/9-EF1α-Flex-taCasp3-TEVp was confirmed by staining brain slices from the mice 3 weeks after virus injection (Figure 4C). The behavior results of the LDT, OFT, and EPM tests demonstrated that ablation of CeM^CRH^ neurons significantly alleviated anxiety-like behaviors in 4T1 tumor–bearing mice (Figure 4, D–M). We found that ablation of CeM^CRH^ neurons significantly inhibited 4T1 tumor growth (Figure 4N) and significantly decreased the weight (Figure 4, O and P), luciferase intensity (Figure 4, Q–R), and NE content (Figure 4, S and T) of 4T1 tumors compared with the control mice. Next, to determine whether the reduced tumor cell proliferation rate and the increased tumor cell apoptosis rate were responsible for the slowed tumor growth observed in the taCasp3 group, we quantified the percentage of Ki67^+^ cells and TUNEL^+^ cells using immunofluorescence staining. Histological analysis revealed that there were significantly fewer Ki67^+^ cells and significantly more TUNEL^+^ cells in 4T1 tumors following ablation of CeM^CRH^ neurons (Figure 4, U–W).

Currently, it is widely believed that the composition of the tumor microenvironment, including the immune cells (such as T cells, macrophages), plays important roles in the progression of cancer and that the sympathetic nervous system is involved in modulation of the immune system (14, 49, 50). Hence, we examined whether ablation of CeM^CRH^ neurons alters antitumor immunity. Mice were subjected to the same injection of viruses to ablate CeM^CRH^ neurons, or to sham ablation, 3 weeks before 4T1 cells injection (Supplemental Figure 8A). Indeed, flow cytometric analysis showed that ablation of CeM^CRH^ neurons significantly increased the percentage of infiltrated CD45^+^ leukocytes in 4T1 tumors (Supplemental Figure 8B). Profiling of the increased infiltrated CD45^+^ leukocytes showed that there were significant increases in the CD4^+^ and CD8^+^ T cell populations following ablation of CeM^CRH^ neurons (Supplemental Figure 8, C and D). Moreover, ablation of CeM^CRH^ neurons significantly decreased the percentage of Tregs (CD4^+^CD25^+^FOXP3^+^ Tregs), CD4^+^PD-1^+^ T cells, and CD8^+^PD-1^+^ T cells, but significantly increased the percentage of CD4^+^IFN-γ^+^ T cells and CD8^+^IFN-γ^+^ T cells in 4T1 tumors (Supplemental Figure 8, E–I). The spleen is an essential organ in systemic antitumor immunity. Therefore, we further examined CD4^+^ and CD8^+^ T cells in spleens from these 4T1 tumor–bearing mice. CeM^CRH^ neuron–ablated mice showed an increased percentage of CD4^+^ and CD8^+^ T cells in spleens compared with mice in the control group (Supplemental Figure 8, J and K). Moreover, ablation of CeM^CRH^ neurons significantly increased the percentage of CD11b^+^F4/80^+^CD86^+^CD206^−^ M1 macrophages and the ratio of M1/M2 macrophages, but did not markedly affect the percentage of CD11b^+^F4/80^+^CD86^−^CD206^+^ M2 macrophages in 4T1 tumors (Supplemental Figure 9).

Additionally, in the MMTV-PyMT mouse model, we also examined the functional role of CeM^CRH^ neurons in the progression of spontaneous mammary tumors by using the caspase-3–based method to ablate the CeM^CRH^ neurons (Supplemental Figure 10, A and B). As expected, ablation of CeM^CRH^ neurons significantly suppressed anxiety-like behaviors of PyMT tumor–bearing mice (Supplemental Figure 10, C–L). Consistently, this ablation also significantly slowed PyMT tumor growth (Supplemental Figure 10M) and significantly decreased the weight and NE content of PyMT tumors (Supplemental Figure 10, O and P). Furthermore, flow cytometric analysis showed that ablation of CeM^CRH^ neurons resulted in significant increases in infiltrated CD45^+^ leukocytes, CD4^+^ T cells, and CD8^+^ T cells in PyMT tumors (Supplemental Figure 11, B–D). Ablation of CeM^CRH^ neurons significantly reduced the percentage of Tregs (CD4^+^CD25^+^FOXP3^+^), CD4^+^PD-1^+^ T cells, and CD8^+^PD-1^+^ T cells, whereas the same manipulation significantly increased the percentage of CD4^+^IFN-γ^+^ T cells and CD8^+^IFN-γ^+^ T cells in PyMT tumors (Supplemental Figure 11, E–I). We observed significant increases in the percentage of CD4^+^ and CD8^+^ T cells in the spleens of PyMT tumor–bearing mice after ablation of CeM^CRH^ neurons (Supplemental Figure 11, J and K). Furthermore, ablation of CeM^CRH^ neurons significantly increased the percentage of CD11b^+^F4/80^+^CD86^+^CD206^−^ M1 macrophages, CD11b^+^F4/80^+^CD86^−^CD206^+^ M2 macrophages, and the ratio of M1/M2 macrophages in PyMT tumors (Supplemental Figure 12). Together, these results suggest that ablation of CeM^CRH^ neurons significantly inhibited cancer-induced anxiety and sympathetic nerve activity and significantly enhanced antitumor immunity, decelerating cancer progression in both orthotopic and spontaneous mammary tumor–bearing mice.

### Inhibition of CeM^CRH^ neurons suppresses cancer-induced anxiety and breast tumor progression.

**Figure 5:**
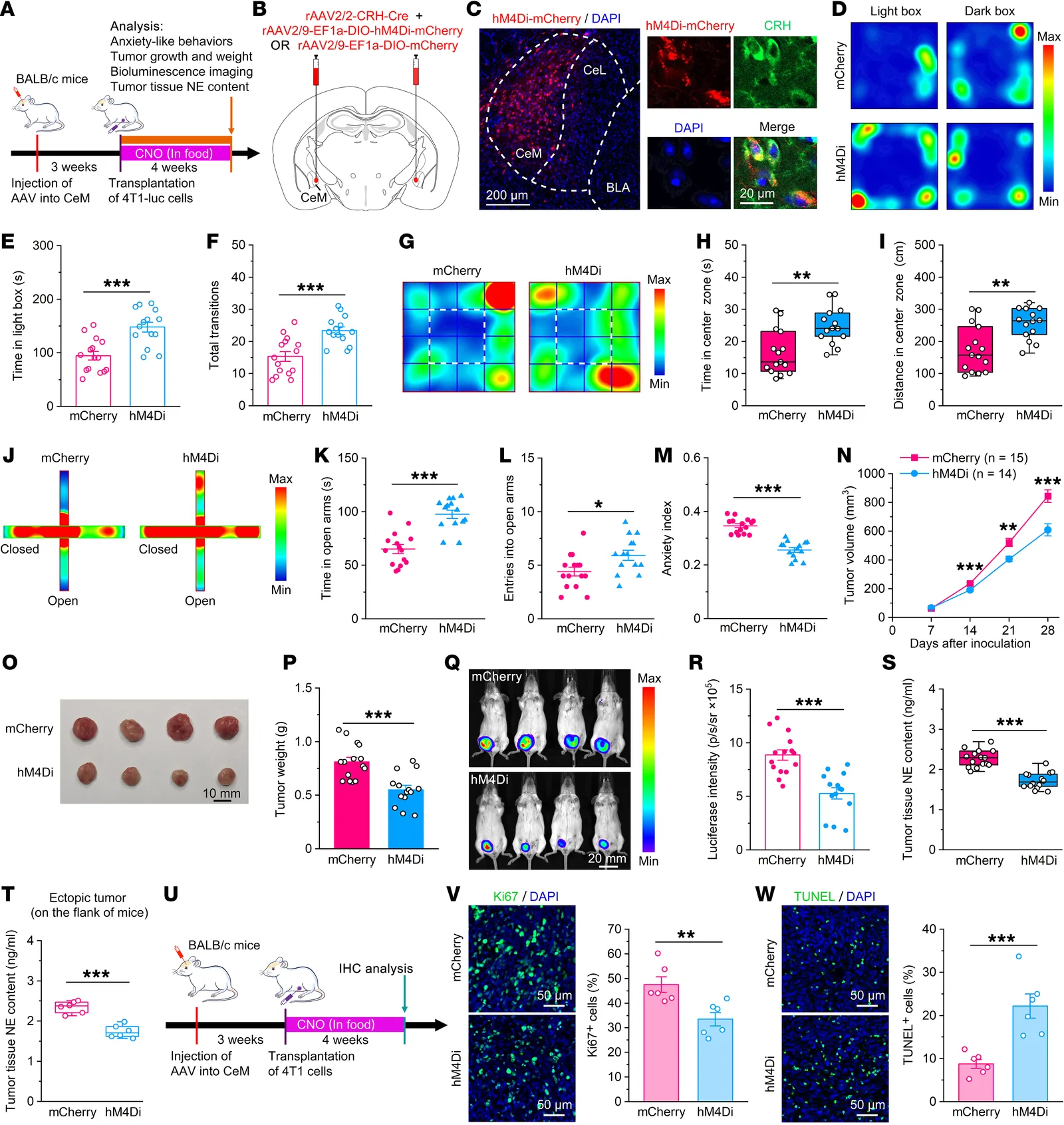
Chemogenetic inhibition of CeM^CRH^ neurons significantly attenuates both cancer-induced anxiety and 4T1 tumor progression. (**A**) Schematic of the experimental design. (**B**) Schematic showing bilateral injection of viruses into the CeM. (**C**) Representative images showing hM4Di-mCherry expression in CeM^CRH^ neurons. Scale bars: 200 μm and 20 μm. (**D**–**M**) Representative heatmaps and summary data of the mCherry (*n* = 15) and hM4Di (*n* = 14) groups in the LDT (**D**–**F**), the OFT (**G**–**I**), and the EPM test (**J**–**M**). (**N**) Chemogenetic inhibition of CeM^CRH^ neurons significantly slowed 4T1 tumor growth. (**O**) Representative tumors dissected from mice of the 2 groups. Scale bar: 10 mm. (**P**) Chemogenetic inhibition of CeM^CRH^ neurons significantly reduced 4T1 tumor weight. (**Q**) Representative bioluminescence images of mice of the 2 groups. Scale bar: 20 mm. (**R**) Chemogenetic inhibition of CeM^CRH^ neurons significantly reduced the luciferase intensity of 4T1 tumors (**P** and **R**: mCherry, *n* = 15, hM4Di, *n* = 14). (**S** and **T**) Chemogenetic inhibition of CeM^CRH^ neurons significantly decreased NE content of 4T1 orthotopic mammary (**S**) and ectopic (**T**) tumors (**S**: mCherry, *n* = 15, hM4Di, *n* = 14; **T**: *n* = 6 for each group). (**U**) Timeline for immunofluorescence staining of 4T1 tumor tissues. (**V** and **W**) Representative images and quantification of Ki67^+^ cells (**V**) and TUNEL^+^ cells (**W**) within 4T1 tumors (*n* = 6 for each group). Scale bars: 50 μm. Data are presented as the mean ± SEM, except in box plots (**H**, **I**, **S**, and **T**), in which center lines indicate the median, box edges represent the first and third quartiles, and whiskers denote minimal and maximal values. *P < 0.05, **P < 0.01, and ***P < 0.001, by 2-way, repeated-measures ANOVA followed by separate 1-way ANOVA (**N**) and 2-tailed, unpaired Student’s *t* test (**E**, **F**, **H**, **I**, **K**–**M**, **P**, **R**–**T**, **V**, and **W**).

Ablation of CeM^CRH^ neurons might cause compensation by altering the neural circuit to regulate the activity of the sympathetic nervous system. Therefore, to further determine the functional role of CeM^CRH^ neurons in the progression of breast cancer, we used the pharmacogenetic method of designer receptors exclusively activated by designer drugs (DREADDs) to inhibit CeM^CRH^ neuron activity and examined the effect on tumor progression. We first injected rAAV2/2-CRH-Cre together with rAAV2/9-EF1α-DIO-hM4Di-mCherry (hM4Di is an inhibitory DREADD receptor, exclusively activated by the “designer drug” clozapine *N*-oxide (CNO) viruses or rAAV2/9-EF1α-DIO-mCherry virus (as a control) into the bilateral CeM. Three weeks after virus injection, we inoculated 4T1 tumor cells into the mammary glands of BALB/c mice (Figure 5, A–C). We observed similar behavioral phenotypes. Selective pharmacogenetic inhibition of CeM^CRH^ neurons in 4T1 tumor–bearing mice significantly reduced anxiety-like behaviors (Figure 5, D–M). Administration of CNO via the diet throughout the remainder of the experiment significantly decelerated 4T1 tumor growth (Figure 5N) and significantly reduced the weight (Figure 5, O and P), luciferase intensity (Figure 5, Q and R), and NE content (Figure 5, S and T) of 4T1 tumors in hM4Di-expressing mice compared with control mice. Immunofluorescence staining showed that pharmacogenetic inhibition of CeM^CRH^ neurons resulted in a significant decrease in the percentage of Ki67^+^ cells, but a significant increase in the percentage of TUNEL^+^ cells in 4T1 tumors (Figure 5, U–W). Notably, as a control, to rule out the possibility that CNO had any effect on the proliferation and apoptosis of 4T1 cells in vitro, 4T1 cells were cultured with or without CNO. As expected, we did not observe a significant effect on the proliferation or apoptosis of 4T1 cells in vitro (Supplemental Figure 13).

In addition, flow cytometric analysis showed that pharmacogenetic inhibition of CeM^CRH^ neurons resulted in significant increases in infiltrated CD45^+^ leukocytes, CD4^+^ T cells, and CD8^+^ T cells in 4T1 tumors (Supplemental Figure 14, B–D). Pharmacogenetic inhibition of CeM^CRH^ neurons significantly reduced the percentage of Tregs (CD4^+^CD25^+^FOXP3^+^), CD4^+^PD-1^+^ T cells, and CD8^+^PD-1^+^ T cells, whereas the same manipulation significantly increased the percentage of CD4^+^IFN-γ^+^ T cells and CD8^+^IFN-γ^+^ T cells in 4T1 tumors (Supplemental Figure 14, E–I). The significant increases in the percentage of CD4^+^ and CD8^+^ T cells in spleens of 4T1 tumor–bearing mice were observed following pharmacogenetic inhibition of CeM^CRH^ neurons (Supplemental Figure 14, J and K). Furthermore, pharmacogenetic inhibition of CeM^CRH^ neurons significantly increased the percentage of CD11b^+^F4/80^+^CD86^+^CD206^−^ M1 macrophages and the ratio of M1/M2 macrophages, but did not significantly affect the percentage of CD11b^+^F4/80^+^CD86^−^CD206^+^ M2 macrophages in 4T1 tumors (Supplemental Figure 15). Notably, these significant differences in antitumor immunity were not due to CNO compound administration, since antitumor immunity did not differ in 4T1 tumor–bearing mice (without virus injections) with or without CNO in their food (Supplemental Figures 16 and 17).

Consistent with the results of pharmacogenetic suppression, optogenetic inhibition (Supplemental Figure 18, A–C; we used AAV encoding light-driven chloride ion pump eNpHR3.0 for optogenetic inhibition) of CeM^CRH^ neurons not only significantly inhibited anxiety-like behaviors of 4T1 tumor–bearing mice (Supplemental Figure 18, D–M), but also significantly slowed 4T1 tumor growth (Supplemental Figure 18N). Moreover, this manipulation also led to significant reductions in the weight (Supplemental Figure 18, O and P), luciferase intensity (Supplemental Figure 18, Q and R), and NE content (Supplemental Figure 18S) of 4T1 tumors. Thus, these results strongly suggest that suppression of CeM^CRH^ neurons significantly inhibited cancer-induced anxiety and sympathetic nerve activity and significantly improved antitumor immunity, delaying cancer progression in mammary tumor–bearing mice.

### Activation of CeM^CRH^ neurons accelerates cancer-induced anxiety and breast tumor progression.

**Figure 6:**
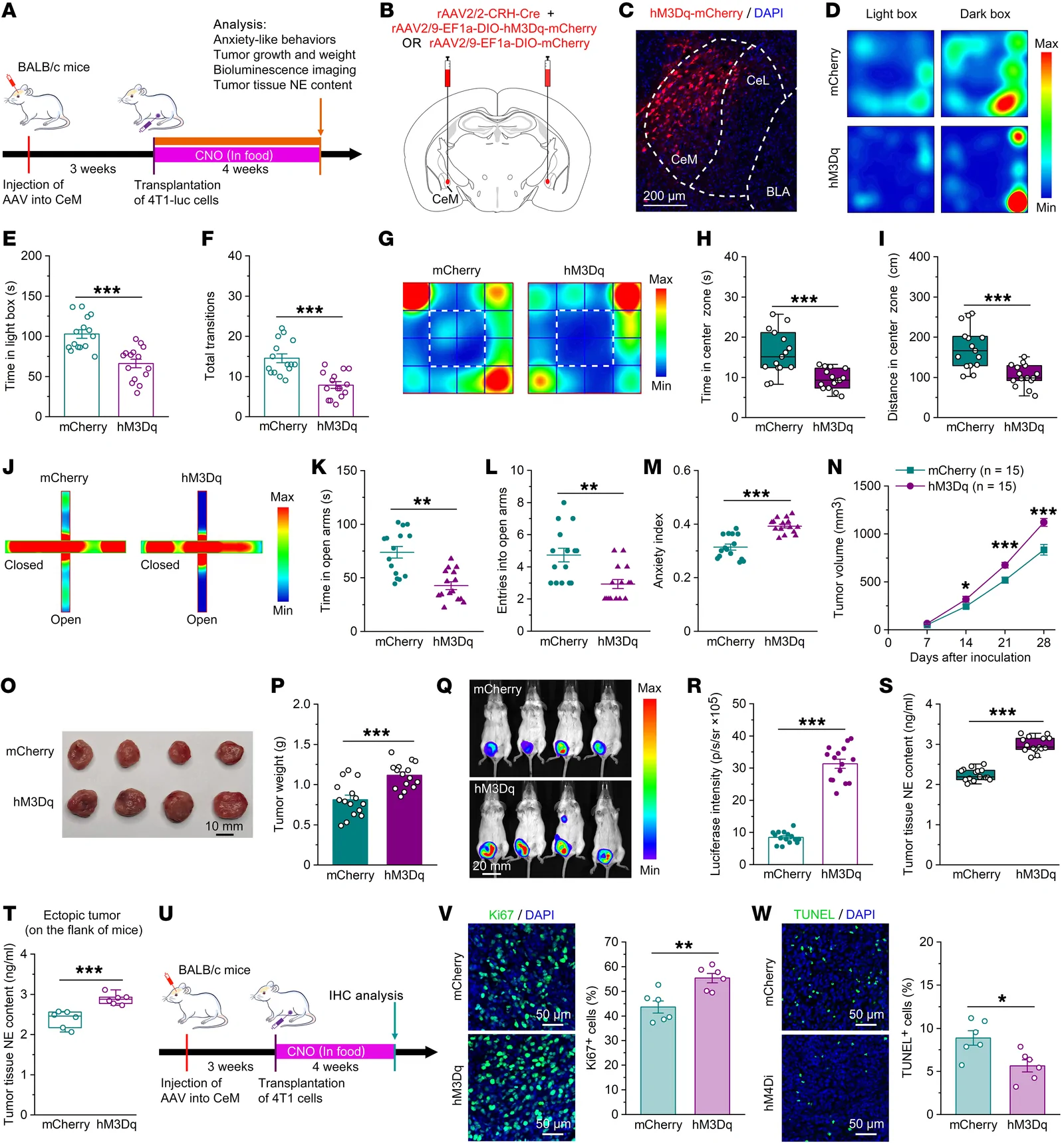
Chemogenetic activation of CeM^CRH^ neurons significantly increases cancer-induced anxiety and accelerates 4T1 tumor progression. (**A**) Schematic illustration of the experimental design. (**B**) Schematic showing bilateral injection of viruses into the CeM. (**C**) Representative image showing hM3Dq-mCherry expression in the CeM. Scale bar: 200 μm.(**D**–**M**) Representative heatmaps and summary data of the mCherry (*n* = 15) and hM3Dq (*n* = 15) groups in the LDT (**D**–**F**), the OFT (**G**–**I**), and the EPM test (**J**–**M**). (**N**) Chemogenetic activation of CeM^CRH^ neurons significantly accelerated 4T1 tumor growth. (**O**) Representative image of tumors dissected from mice of the 2 groups. Scale bar: 10 mm. (**P**) Chemogenetic activation of CeM^CRH^ neurons significantly increased 4T1 tumor weight. (**Q**) Representative bioluminescence images of mice of the 2 groups. Scale bar: 20 mm. (**R**) Chemogenetic activation of CeM^CRH^ neurons significantly increased the luciferase intensity of 4T1 tumors (**P** and **R**: *n* = 15 for each group). (**S** and **T**) Chemogenetic activation of CeM^CRH^ neurons significantly increased NE content of 4T1 orthotopic mammary (**S**) and ectopic (**T**) tumors (**S**: *n* = 15 for each group; **T**: *n* = 6 for each group). (**U**) Timeline for immunofluorescence staining of 4T1 tumor tissues. (**V** and **W**) Representative images and quantification of Ki67^+^ cells (**V**) and TUNEL^+^ cells (**W**) within 4T1 tumors (*n* = 6 for each group). Scale bars: 50 μm. Data are presented as the mean ± SEM, except in box plots (**H**, **I**, **S**, and **T**), in which center lines indicate the median, box edges represent the first and third quartiles, and whiskers denote minimal and maximal values. *P < 0.05, **P < 0.01, and ***P < 0.001, by 2-way, repeated-measures ANOVA followed by separate 1-way ANOVA (**N**) and 2-tailed, unpaired Student’s *t* test (**E**, **F**, **H**, **I**, **K**–**M**, **P**, **R**–**T**, **V**, and **W**).

To gain further insight into the crucial role of activated CeM^CRH^ neurons in breast tumor progression, we also increased the activity of CeM^CRH^ neurons with pharmacogenetics and optogenetics. In pharmacogenetic activation experiments (Figure 6, A–C), CNO administration to 4T1 tumor–bearing mice expressing a stimulatory DREADD receptor (hM3Dq) in CeM^CRH^ neurons resulted in a significant increase in anxiety-like behaviors compared with 4T1 tumor–bearing mice expressing the control mCherry fluorescent protein in CeM^CRH^ neurons (Figure 6, D–M). Furthermore, pharmacogenetic activation of CeM^CRH^ neurons not only significantly accelerated the 4T1 tumor growth rate (Figure 6N), but also significantly increased the weight (Figure 6, O and P), luciferase intensity (Figure 6, Q and R), and NE content (Figure 6, S and T) of 4T1 tumors. Immunofluorescence staining showed that pharmacogenetic activation of CeM^CRH^ neurons resulted in a significant increase in the percentage of Ki67^+^ cells, but a significant decrease in the percentage of TUNEL^+^ cells in 4T1 tumors (Figure 6, U–W). In addition, flow cytometric analysis showed that pharmacogenetic activation of CeM^CRH^ neurons resulted in significant decreases in infiltrated CD45^+^ leukocytes, CD4^+^ T cells, and CD8^+^ T cells in 4T1 tumors (Supplemental Figure 14, B–D). Pharmacogenetic activation of CeM^CRH^ neurons significantly increased the percentage of Tregs (CD4^+^CD25^+^FOXP3^+^), CD4^+^PD-1^+^ T cells, and CD8^+^PD-1^+^ T cells, but significantly decreased the percentage of CD4^+^IFN-γ^+^ T cells and CD8^+^IFN-γ^+^ T cells in 4T1 tumors (Supplemental Figure 14, E–I). The significant decreases in the percentage of CD4^+^ and CD8^+^ T cells in the spleens of 4T1 tumor–bearing mice were observed following pharmacogenetic activation of CeM^CRH^ neurons (Supplemental Figure 14, J and K). Furthermore, pharmacogenetic activation of CeM^CRH^ neurons significantly decreased the percentage of CD11b^+^F4/80^+^CD86^+^CD206^−^ M1 macrophages and the ratio of M1/M2 macrophages, but did not significantly affect the percentage of CD11b^+^F4/80^+^CD86^−^CD206^+^ M2 macrophages in 4T1 tumors (Supplemental Figure 15).

Similar results were obtained in optogenetic activation experiments. We also found that optogenetic activation of CeM^CRH^ neurons (Supplemental Figure 19, A–C) not only significantly increased anxiety-like behaviors of 4T1 tumor–bearing mice (Supplemental Figure 19, D–M), but also increased the growth (Supplemental Figure 19N), weight (Supplemental Figure 19, O and P), luciferase intensity (Supplemental Figure 19, Q and R), and NE content (Supplemental Figure 19S) of 4T1 tumors. Thus, these lines of evidence suggest that activation of CeM^CRH^ neurons significantly increased the cancer-induced anxiety and sympathetic nerve activity and significantly suppressed antitumor immunity, thereby promoting cancer progression in mammary tumor–bearing mice.

### Inhibition of the CeM^CRH^→LPGi circuit suppresses cancer-induced anxiety and breast tumor progression.

**Figure 7:**
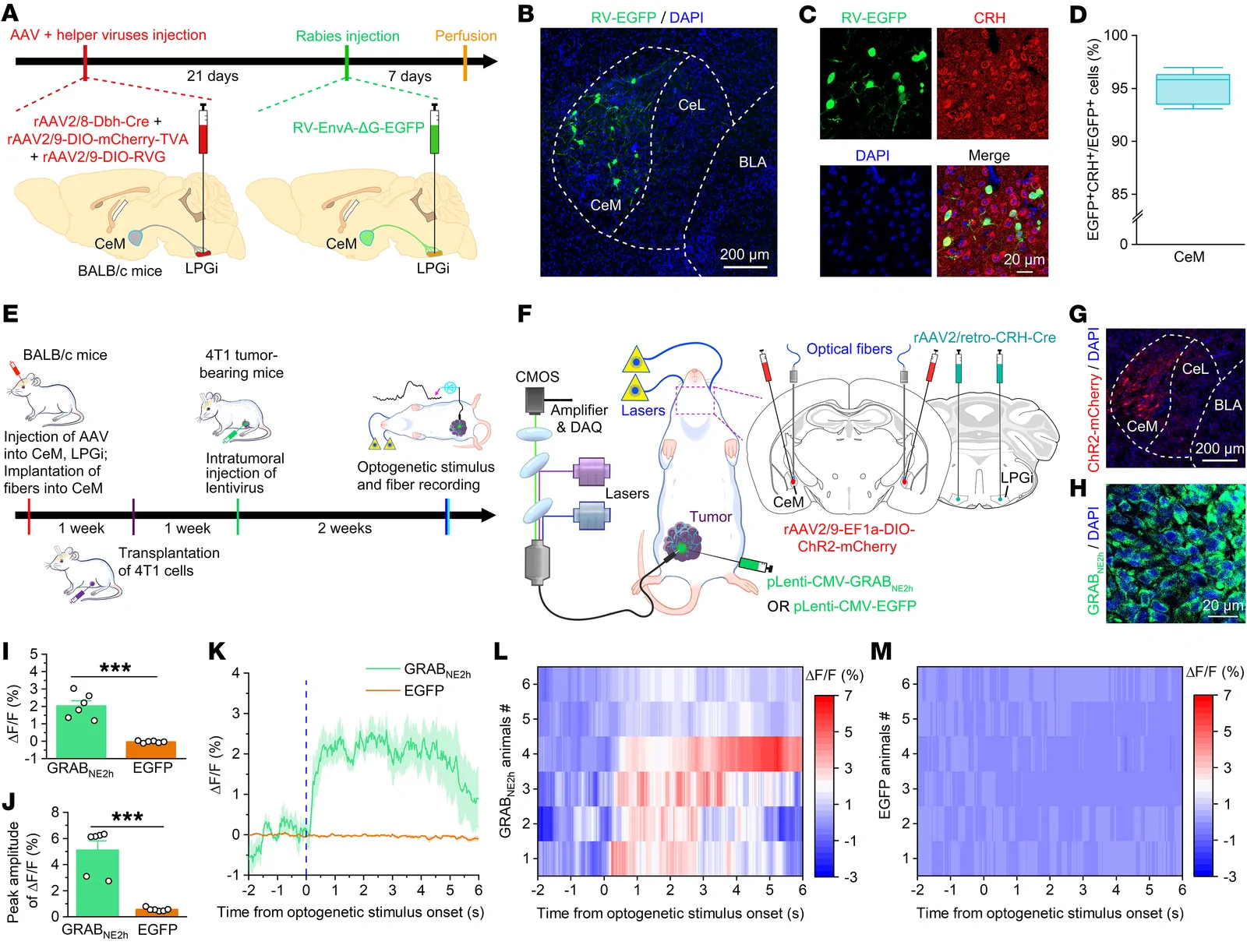
Optogenetic stimulation of the CeM^CRH^→LPGi circuit activates sympathetic nerves in 4T1 tumors. (**A**) Schematic of the Cre-dependent retrograde transmonosynaptic rabies virus–tracing strategy in WT BALB/c mice. (**B**) EGFP-labeled neurons in the CeM traced from LPGi^CA^ neurons. Scale bar: 200 μm. (**C** and **D**) Representative images showing colocalization of EGFP with CRH in the CeM (**C**) and summarized data (**D**; *n* = 5). Scale bar: 200 μm. (**E** and **F**) Timeline and scheme for recording the activities of sympathetic nerves distributed in the tumor stroma during optogenetic stimulation of the CeM^CRH^→LPGi circuit in the CeM. (**G** and **H**) Typical image of viruses (AAV and pLenti) expression in the CeM (**G**) and tumor stroma (**H**). Scale bars: 200 um (**G**) and 20 um (**H**). (**I** and **J**) Comparison of the mean ΔF/F (0–5 s) (**I**) and peak amplitude of ΔF/F (**J**) between the GRAB_NE2h_ and EGFP groups (*n* = 6 for each group). (**K**) Average fluorescence change in the GRAB_NE2h_ and EGFP groups, with shaded areas indicating the SEM. (**L** and **M**) Heatmaps show the average fluorescence change in the GRAB_NE2h_ (**L**) and EGFP (**M**) groups. Data are presented as the mean ± SEM, except in box and half violin plots (**D**), in which center lines indicate the median, box edges represent the first and third quartiles, and whiskers denote minimal and maximal values. ***P < 0.001, by 2-tailed, unpaired Student’s *t* test (**I** and **J**).

Because distinct subpopulations of CeM^CRH^ neurons project to different hypothalamic and brain stem structures, it is unclear which of these projections participates in the circuit that modulates cancer-induced anxiety and breast tumor progression. Our data showed that there was a neural connection between the sympathetic nerves distributed in the tumor stroma and LPGi^CA^ neurons (Figure 2M and Supplemental Figure 5G). Moreover, previous studies suggested that LPGi^CA^ neurons innervate sympathoadrenal preganglionic neurons (33, 45). To identify monosynaptic projections from CeM^CRH^ neurons to LPGi^CA^ neurons, we used a cell-type–specific retrograde transmonosynaptic tracing system (Figure 7A). We injected rAAV2/8-Dbh-Cre virus together with Cre-dependent AAV-helper viruses into the LPGi region. After 21 days, the retrograde transmonosynaptic rabies virus RV-EnvA-ΔG-EGFP was injected at the same site (Figure 7A). The histological results showed that EGFP-labeled neurons were located in the CeM (Figure 7B). Immunofluorescence staining showed that most of the EGFP-labeled neurons in the CeM colocalized with CRH (Figure 7, C and D). These data suggest that CeM^CRH^ neurons sent monosynaptic projections to LPGi^CA^ neurons. In addition, fiber photometric recordings in 4T1 tumors also indicated that direct optogenetic stimulation of LPGi-projecting CRH neurons in the CeM (CeM^CRH^→LPGi circuit) also induced a robust increase in fluorescent signals of GRAB_NE2h_ in tumor tissue. In contrast, the fluorescent signals in control mice expressing EGFP in tumor tissue showed no significant change during optogenetic stimulation (Figure 7, E–M).

**Figure 8:**
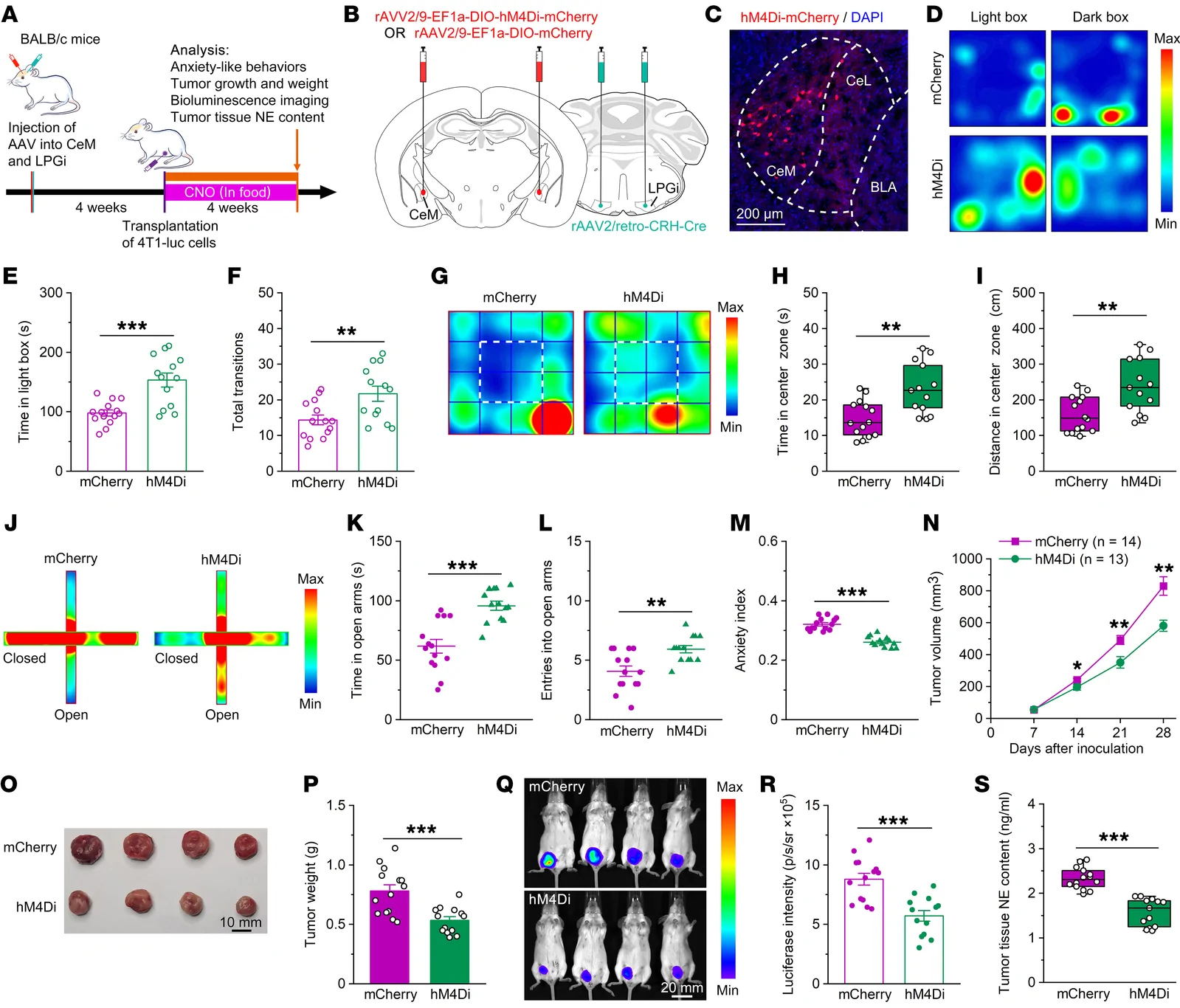
Chemogenetic inhibition of the CeM^CRH^→LPGi circuit significantly suppresses both cancer-induced anxiety and 4T1 tumor progression. (**A**) Schematic of the experimental design. (**B**) Schematic showing bilateral injection of rAAV2/retro-CRH-Cre into the LPGi and of rAAV2/9-EF1α-DIO-hM4Di-mCherry or rAAV2/9-EF1α-DIO-mCherry into the CeM. (**C**) Representative images showing hM4Di-mCherry expression in the CeM. Scale bar: 200 μm. (**D**–**M**) Representative heatmaps and summary data of the mCherry (*n* = 14) and hM4Di (*n* = 13) groups in the LDT (**D**–**F**), the OFT (**G**–**I**), and the EPM test (**J**–**M**). (**N**) Chemogenetic inhibition of the CeM^CRH^→LPGi circuit significantly decelerated 4T1 tumor growth. (**O**) Representative tumors dissected from mice of the 2 groups. Scale bar: 10 mm. (**P**) Chemogenetic inhibition of the CeM^CRH^→LPGi circuit significantly reduced 4T1 tumor weight. (**Q**) Representative bioluminescence images of mice of the 2 groups. Scale bar: 20 mm. (**R** and **S**) Chemogenetic inhibition of the CeM^CRH^→LPGi circuit significantly reduced the luciferase intensity of 4T1 tumors (**R**) and tumor tissue NE content (**S**) (**P**, **R**, and **S**: mCherry, *n* = 14, hM4Di, *n* = 13). Data are presented as the mean ± SEM, except in box plots (**H**, **I**, and **S**), in which center lines indicate the median, box edges represent the first and third quartiles, and whiskers denote minimal and maximal values. *P < 0.05, **P < 0.01, and ***P < 0.001, by 2-way, repeated-measures ANOVA followed by separate 1-way ANOVA (**N**) and 2-tailed, unpaired Student’s *t* test (**E**, **F**, **H**, **I**, **K**–**M**, **P**, **R**, and **S**).

To address whether the anxiety-induced hyperactivation of the CeM^CRH^→LPGi circuit alters breast tumor progression, we performed bilateral injection of retrogradely transported retro-AAV expressing Cre recombinase (rAAV2/retro-CRH-Cre) into the LPGi and a Cre-dependent AAV encoding hM4Di-mCherry or mCherry (rAAV2/9-EF1α-DIO-hM4Di-mCherry or rAAV2/9-EF1α-DIO-mCherry) into the CeM (Figure 8, A–C). As expected, we found that pharmacogenetic suppression of the CeM^CRH^→LPGi circuit not only significantly alleviated anxiety-like behaviors (Figure 8, D–M), but also significantly decreased the tumor growth rate (Figure 8N), weight (Figure 8, O and P), luciferase intensity (Figure 8, Q and R), and NE content (Figure 8S) of 4T1 tumors.

Likewise, consistent with the effects of pharmacogenetic suppression, optogenetic inhibition (Supplemental Figure 20, A–C) of the CeM^CRH^→LPGi circuit also significantly inhibited anxiety-like behaviors (Supplemental Figure 20, D–M) and significantly decreased the tumor growth rate (Supplemental Figure 20N), weight (Supplemental Figure 20, O and P), luciferase intensity (Supplemental Figure 20, Q and R), and NE content (Supplemental Figure 20S) of 4T1 tumors. Thus, these results provide valid evidence to support the notion that inhibition of CeM^CRH^→LPGi significantly suppresses cancer-induced anxiety, sympathetic nerve activity, and tumor progression in mammary tumor–bearing mice.

### Activation of the CeM^CRH^→LPGi circuit accelerates cancer-induced anxiety and breast tumor progression.

**Figure 9:**
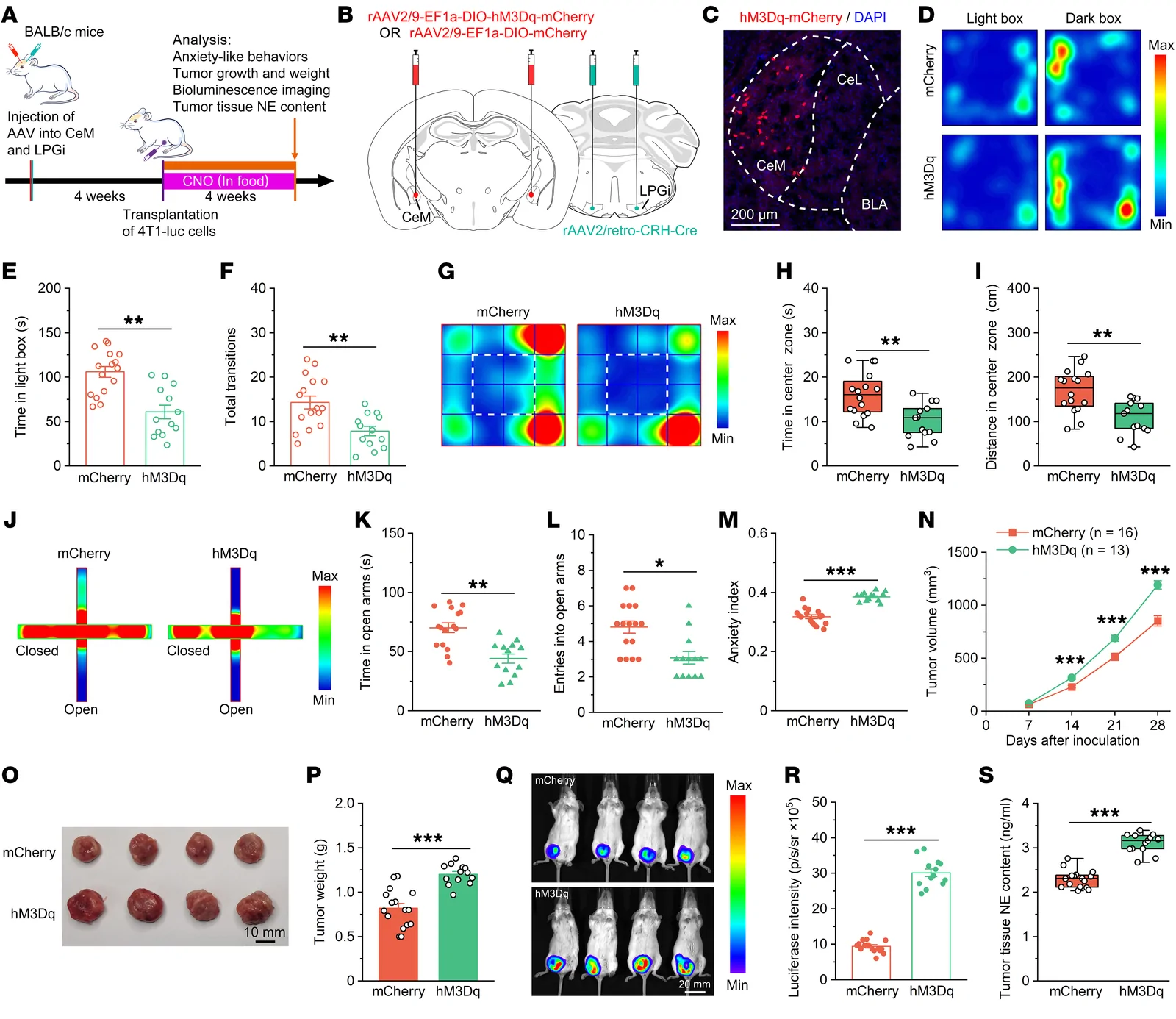
Chemogenetic activation of the CeM^CRH^→LPGi circuit significantly increases cancer-induced anxiety and accelerates 4T1 tumor progression. (**A**) Schematic illustration of the experimental design. (**B**) Schematic showing bilateral injection of rAAV2/retro-CRH-Cre into the LPGi and of rAAV2/9-EF1α-DIO-hM3Dq-mCherry or rAAV2/9-EF1α-DIO-mCherry into the CeM. (**C**) Representative images showing hM3Dq-mCherry expression in the CeM. Scale bar: 200 μm. (**D**–**M**) Representative heatmaps and summary data for the mCherry (*n* = 16) and hM3Dq (*n* = 13) groups in the LDT (**D**–**F**), the OFT (**G**–**I**), and the EPM test (**J**–**M**). (**N**) Chemogenetic activation of the CeM^CRH^→LPGi circuit significantly accelerated 4T1 tumor growth. (**O**) Representative image of tumors dissected from mice of the 2 groups. Scale bar: 10 mm. (**P**) Chemogenetic activation of the CeM^CRH^→LPGi circuit significantly increased 4T1 tumor weight. (**Q**) Representative bioluminescence images of mice of the 2 groups. Scale bar: 20 mm. (**R** and **S**) Chemogenetic activation of the CeM^CRH^→LPGi circuit significantly increased the luciferase intensity of 4T1 tumors (**R**) and tumor tissue NE content (**S**). (**P**, **R**, and **S**: mCherry, *n* = 16, hM3Dq, *n* = 13). Data are presented as the mean ± SEM, except in box plots (**H**, **I**, and **S**), in which center lines indicate the median, box edges represent the first and third quartiles, and whiskers denote minimal and maximal values. *P < 0.05, **P < 0.01, and ***P < 0.001, by 2-way, repeated-measures ANOVA followed by separate 1-way ANOVA (**N**) and 2-tailed, unpaired Student’s *t* test (**E**, **F**, **H**, **I**, **K**–**M**, **P**, **R**, and **S**).

Next, to confirm and further examine whether anxiety-induced activation of the CeM^CRH^→LPGi circuit affects tumor progression, we bilaterally injected a retrograde rAAV2/retro-CRH-Cre virus into the LPGi and a Cre-dependent rAAV2/9-EF1α-DIO-hM3Dq-mCherry or rAAV2/9-EF1α-DIO-mCherry (as a control) virus into the CeM (Figure 9, A–C). When CNO was administered from day 1 after tumor cell inoculation, anxiety-like behaviors of 4T1 tumor–bearing tumor mice expressing hM3Dq were significantly increased compared with control mice (Figure 9, D–M). Conversely, pharmacogenetic activation of the CeM^CRH^→LPGi circuit significantly increased the tumor growth rate (Figure 9N), weight (Figure 9, O and P), luciferase intensity (Figure 9, Q and R), and NE content (Figure 9S) of 4T1 tumors.

In addition, we also observed that optogenetic activation of the CeM^CRH^→LPGi circuit not only significantly enhanced anxiety-like behaviors (Supplemental Figure 21, D–M), but also significantly increased the tumor growth rate (Supplemental Figure 21N), weight (Supplemental Figure 21, O and P), luciferase intensity (Supplemental Figure 21, Q and R), and NE content (Supplemental Figure 21S) of 4T1 tumors. Collectively, these results suggest that activation of the CeM^CRH^→LPGi circuit significantly increased cancer-induced anxiety, sympathetic nerve activity, and tumor progression in mammary tumor–bearing mice.

### Alprazolam is a promising agent for slowing breast tumor progression.

**Figure 10:**
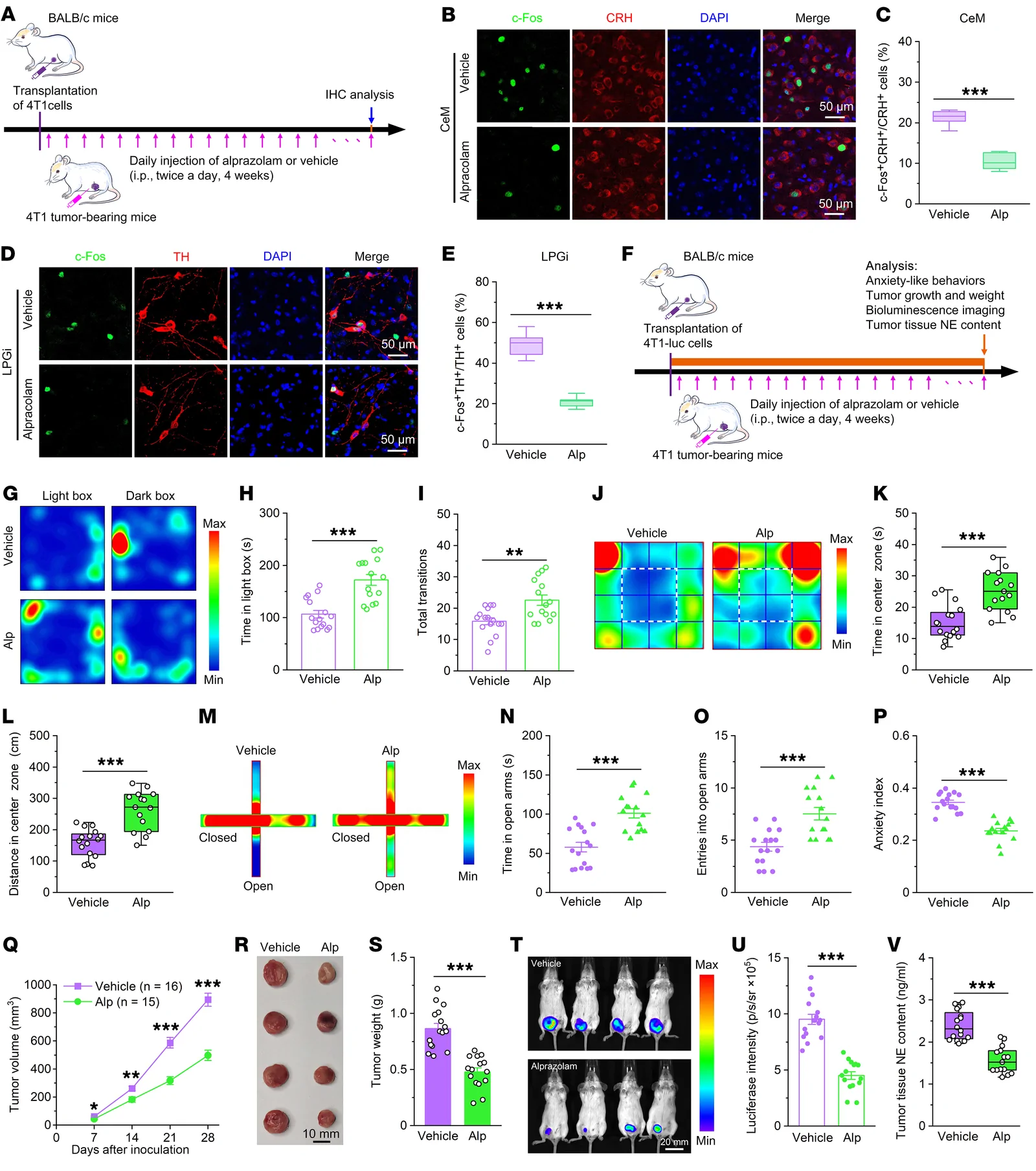
Alprazolam treatment significantly inhibits the activity of CeM^CRH^ neurons and LPGi^CA^ neurons and decelerates the progression of 4T1 breast tumors. (**A**) Experimental protocol for treatment with alprazolam or vehicle and immunofluorescence staining. (**B**–**E**) Representative images and summarized data for c-Fos expression in CeM ^CRH^ neurons (**B** and **C**) and LPGi^CA^ neurons (**D** and **E**) after treatment with alprazolam (Alp) or vehicle (*n* = 5 for each group). Scale bars: 50 μm (**B** and **D**). (**F**) Schematic overview of the experimental design. (**G**–**P**) Representative heatmaps and summary data for vehicle (*n* = 16) and alprazolam (*n* = 15) treatment groups in the LDT (**G**–**I**), the OFT (**J**–**L**), and the EPM test (**M**–**P**). (**Q**) Alprazolam treatment significantly decelerated 4T1 tumor growth. (**R**) Representative image of tumors dissected from mice of the 2 groups. Scale bar: 10 mm. (**S**) Alprazolam treatment significantly reduced 4T1 tumor weight. (**T**) Representative bioluminescence images of mice of the 2 groups. Scale bar: 20 mm. (**U** and **V**) Alprazolam treatment significantly reduced the luciferase intensity of 4T1 tumors (**U**) and tumor tissue NE content (**V**). (**S**, **U**, and **V**: vehicle, *n* = 16, alprazolam, *n* = 15). Data are presented as the mean ± SEM, except in box plots (**C**, **E**, **K**, **L**, and **V**), in which center lines indicate the median, box edges represent the first and third quartiles, and whiskers denote minimal and maximal values. *P < 0.05, **P < 0.01, and ***P < 0.001, by 2-way, repeated-measures ANOVA followed by separate 1-way ANOVA (**Q**) and 2-tailed, unpaired Student’s *t* test (**C**, **E**, **H**, **I**, **K**, **L**, **N**–**P**, **S**, **U**, and **V**).

The present results suggest that cancer-induced anxiety and overactivity of CeM^CRH^ neurons play an important role in the progression of breast tumors. Alprazolam is widely used to treat anxiety disorders (51, 52). Moreover, it has been shown that acute or chronic treatment with alprazolam or other benzodiazepines significantly reduced amygdala activity (53–56). First, to determine whether treatment with alprazolam affects the activity of CeM^CRH^ and LPGi^CA^ neurons in tumor-bearing mice, we examined the activity of CeM^CRH^ and LPGi^CA^ neurons using c-Fos as a neuronal activity marker. Double-immunofluorescence staining showed that the percentage of c-Fos^+^ CRH^+^ neurons among CeM^CRH^ neurons and of c-Fos^+^ CA^+^ neurons among LPGi^CA^ neurons decreased significantly after daily injection of alprazolam (Figure 10, A–E), suggesting that alprazolam treatment could significantly reduce the activity of CeM^CRH^ and LPGi^CA^ neurons in tumor-bearing mice.

Next, we tested the therapeutic effects of alprazolam on cancer-induced anxiety and breast tumor progression. Following injection of 4T1 cells, tumor-bearing mice were subjected to twice-daily injection of alprazolam or vehicle for 4 weeks (Figure 10F). Daily treatment with alprazolam significantly alleviated anxiety-like behaviors (Figure 10, G–P) and significantly decreased the tumor growth rate (Figure 10Q), weight (Figure 10, R and S), luciferase intensity (Figure 10, T and U), and NE content (Figure 10V) of 4T1 tumors. Moreover, flow cytometric analysis showed that daily treatment with alprazolam (Supplemental Figure 23A) resulted in significant increases in infiltrated CD45^+^ leukocytes, CD4^+^ T cells, and CD8^+^ T cells in 4T1 tumors (Supplemental Figure 23, B–D). Alprazolam treatment significantly reduced the percentage of Tregs (CD4^+^CD25^+^FOXP3^+^), CD4^+^PD-1^+^ T cells, and CD8^+^PD-1^+^ T cells and significantly increased the percentage of CD4^+^IFN-γ^+^ T cells and CD8^+^IFN-γ^+^ T cells in PyMT tumors (Supplemental Figure 23, E–I). We also observed significant increases in the percentage of CD4^+^ and CD8^+^ T cells in the spleens of 4T1 tumor–bearing mice following alprazolam treatment (Supplemental Figure 23, J and K). Furthermore, alprazolam treatment significantly increased the percentage of CD11b^+^F4/80^+^CD86^+^CD206^−^ M1 macrophages and the ratio of M1/M2 macrophages, but did not significantly affect the percentage of CD11b^+^F4/80^+^CD86^−^CD206^+^ M2 macrophages in 4T1 tumors (Supplemental Figure 24). Notably, as a control, to rule out any possibility of an effect of alprazolam on proliferation and apoptosis of 4T1 cells in vitro, 4T1 cells were cultured with or without alprazolam. As expected, we observed no significant effect on the proliferation and apoptosis of 4T1 cells in vitro (Supplemental Figure 22).

**Figure 11:**
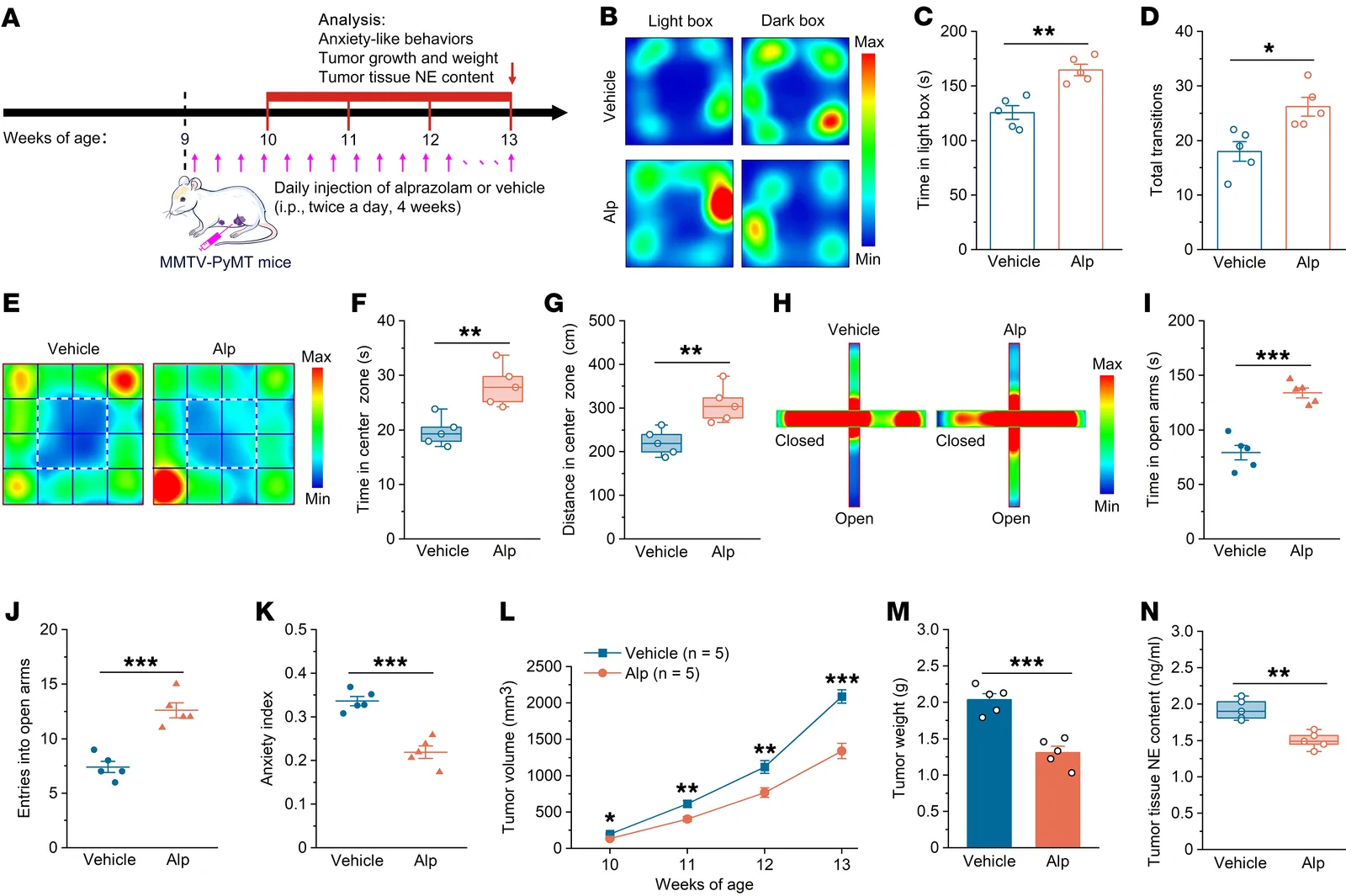
Alprazolam treatment significantly reduces cancer-induced anxiety and suppresses tumor progression in MMTV-PyMT mice. (**A**) Schematic of the experimental design. (**B**–**K**) Representative heatmaps and summary data for the vehicle (*n* = 5) and alprazolam (*n* = 5) treatment groups in the LDT (**B**–**D**), the OFT (**E**–**G**), and the EPM test (**H**–**K**). (**L**) Alprazolam treatment significantly decelerated PyMT tumor growth. (**M**) Alprazolam treatment significantly reduced PyMT tumor weight. (**N**) Alprazolam treatment significantly reduced the NE content of PyMT tumor tissue (*n* = 5 for each group). Data are presented as the mean ± SEM, except in box plots (**F**, **G**, and **N**), in which center lines indicate the median, box edges represent the first and third quartiles, and whiskers denote minimal and maximal values. *P < 0.05, **P < 0.01, and ***P < 0.001, by 2-way, repeated-measures ANOVA followed by separate 1-way ANOVA (**M**) and 2-tailed, unpaired Student’s *t* test (**C**, **D**, **F**, **G**, **I**–**K**, and **M**).

Additionally, in the MMTV-PyMT mouse model, we also examined the functional role of alprazolam treatment in cancer-induced anxiety and progression of spontaneous mammary tumors (Figure 11A). As expected, we found that alprazolam treatment significantly suppressed anxiety-like behaviors of PyMT tumor–bearing mice (Figure 11, B–K). Consistently, the alprazolam treatment also significantly slowed PyMT tumor growth (Figure 11L) and significantly decreased the weight and NE content of PyMT tumors (Figure 11, M and N). In addition, flow cytometric analysis showed that alprazolam treatment (Supplemental Figure 25A) resulted in significant increases in infiltrated CD45^+^ leukocytes, CD4^+^ T cells, and CD8^+^ T cells in PyMT tumors (Supplemental Figure 25, B–D). Alprazolam significantly reduced the percentage of Tregs (CD4^+^CD25^+^FOXP3^+^), CD4^+^PD-1^+^ T cells, and CD8^+^PD-1^+^ T cells, whereas the same manipulation resulted in a significant increase in the percentage of CD4^+^IFN-γ^+^ T cells and CD8^+^IFN-γ^+^ T cells in PyMT tumors (Supplemental Figure 25, E–I). We observed significant increases in the percentage of CD4^+^ and CD8^+^ T cells in the spleens of PyMT tumor–bearing mice after ablation of CeM^CRH^ neurons (Supplemental Figure 25, J and K). Furthermore, alprazolam treatment also significantly increased the percentage of CD11b^+^F4/80^+^CD86^+^CD206^−^ M1 macrophages, CD11b^+^F4/80^+^CD86^−^CD206^+^ M2 macrophages, and the ratio of M1/M2 macrophages in PyMT tumors (Supplemental Figure 26). Taken together, these results suggest that alprazolam treatment significantly inhibited the activity of CeM^CRH^ and LPGi^CA^ neurons, cancer-induced anxiety, and sympathetic nerve activity and also significantly enhanced antitumor immunity, slowing the progression of cancer in both orthotopic and spontaneous mammary tumor–bearing mice.

## Discussion

Unlike antiangiogenic and immunomodulatory therapies, which have become a mainstay of clinical oncology, we are only beginning to uncover how the nervous system modulates cancer growth (14, 16, 49). Notably, several studies have shown that peripheral nerves are emerging regulators of cancer progression (19, 21, 22). Peripheral nerves may underlie the increased progression and mortality of cancer patients with high psychosocial stress. Although peripheral nerves are ultimately connected to the brain, the neural mechanism underlying anxiety-promoted tumor progression remains unclear. In this study, we used interdisciplinary approaches combining cancer research and neuroscience to examine how the brain contributes to tumor progression via direct tumor-nerve crosstalk. We showed that newly formed sympathetic nerves were distributed in breast tumors at the early stage of progression and that the nerves were polysynaptically connected to CeM^CRH^ neurons. Additionally, CeM^CRH^ neurons and the CeM^CRH^→LPGi circuit, an upstream neural circuit of the sympathetic nervous system, were activated in breast tumor–bearing mice. Artificial manipulation of the activity of CeM^CRH^ neurons and the CeM^CRH^→LPGi circuit significantly affected cancer-induced anxiety, sympathetic nerve activity, antitumor immunity, and cancer progression. Together, we have established a causal link between the brain activity of CeM^CRH^ neurons and tumor growth. We demonstrate the crucial role of a brain-tumor circuit underlying cancer-induced anxiety via sympathetic innervation that controls breast tumor progression.

It remains to be shown whether targeting brain-tumor crosstalk with specific therapies can lead to clinical benefits (15). In preclinical experiments, tricyclic antidepressants have been shown to control the growth of breast tumors, and relevant phase I clinical trial are currently underway (57). As a commonly used antianxiety drug, benzodiazepine (such as alprazolam) is generally considered to be beneficial for patients with cancer-induced anxiety (13, 58, 59). We found that alprazolam significantly reduced CeM^CRH^ and LPGi^CA^ neuron activity, cancer-induced anxiety, and sympathetic nerve activity, and then observably improved antitumor immunity and slowed tumor progression in tumor-bearing mice. The results suggested that antianxiety drugs and neural circuit interventions could be a potential avenue for the treatment of breast cancer.

Prior studies have shown that innervation occurred only after the tumor grew for a period of time (21, 22). However, we discovered that in the initial phases of tumor growth (5 days), there was obvious new sympathetic nerve growth in the tumor. These results indicated that sympathetic nerves may be involved in active regulation of breast cancer progression from the initial phases of tumor development.

This study aimed to demonstrate a functional connection between an emotion-regulating neuronal circuit and tumor growth. We showed that manipulation of CeM^CRH^ neurons or the CeM^CRH^→LPGi circuit resulted in the direct regulation of intratumoral sympathetic nerve activity, local levels of NE, and tumor progression. Previous studies have confirmed that peripheral sympathetic innervation regulates cancer initiation and development (19, 21, 22, 60–62). As a key factor in nerve-cancer crosstalk in the tumor microenvironment, the neurotransmitter NE transmits sympathetic signals to various cells through adrenergic receptors (ARs) and plays multiple roles in tumor development (15, 63, 64). Anxiety is considered to induce activation of the sympathetic nervous system (23, 24), and then the released NE acts directly on cancer cells to affect tumor progression (21, 22, 65). Several studies have shown that excessive activation of the α2-AR significantly accelerates breast cancer progression. For example, dexmedetomidine, a highly selective α2-AR agonist, has been reported to significantly increase the proliferation, migration, and invasion of the breast cancer cell lines MCF-7 and MDA-MB-231 in vitro by activating the α2-AR and downstream signaling pathways (66–68). Moreover, dexmedetomidine could also significantly elevate the weight and volume of MDA-MB-231 breast tumors in vivo (67). In contrast, suppression of the α2-AR could significantly inhibit the proliferation, migration, and invasion of MDA-MB-231 cells in vivo (69). Additionally, it has been proven that blocking the β-AR significantly slows tumor growth and progression of both MDA-MB-231 and BT-549 breast tumors in vivo (22). Concordantly, in several clinical studies, treatment with beta blockers significantly reduced the biomarkers and pathways associated with metastasis in patients with breast cancer (70–72). These results suggest that local sympathetic nerves can influence the progression of breast cancer by secreting NE, which directly binds to the corresponding receptors of breast cancer cells.

In addition, the other possible mechanism underlying sympathetic innervation–affected tumor progression is that the secreted NE indirectly acts on immune cells to modulate antitumor immunity (16, 65). It is generally believed that sympathetic nerves directly innervate all primary and secondary immune organs to regulate immunity and that sympathetic activity suppresses antitumor immunity (50, 73). Indeed, our results also showed that the hyperactivation of intratumoral sympathetic nerves via artificial activation of CeM^CRH^ neurons resulted in significant decreases in infiltrated CD45^+^ leukocytes, CD4^+^ T cells, CD8^+^ T cells, CD4^+^IFN-γ^+^ T cells, and CD8^+^IFN-γ^+^ T cells, but significantly increased Tregs, CD4^+^PD-1^+^ T cells, and CD8^+^PD-1^+^ T cells in tumors. The M1-like phenotype of the tumor microenvironment is immunostimulatory and can restrain tumor development and progression (74, 75). Correspondingly, our data showed that activation of intratumoral sympathetic nerves significantly inhibited M1 polarization and reduced the M1/M2 macrophage ratio in tumors. In contrast, suppression of local sympathetic activity through artificial inhibition of the brain slowed tumor growth by decreasing local levels of NE and enhancing antitumor immunity. Collectively, these findings raise the possibility that cancer-induced anxiety activates sympathetic nerves, which in turn inhibits the antitumor immune response and promotes the progression of breast cancer. It is probably also worth noting that 4T1 cells do not express functional ARs, but that sympathetic nerves also regulate the 4T1 tumor growth via modulation of the immune system (20). Nevertheless, it is likely that such a brain manipulation will also affect other physiological systems that can also contribute to tumor growth. For example, it has also been shown that intratumoral sympathetic nerves release NE to accelerate tumor growth by promoting angiogenesis (61, 63). Thus, attention should be given not only to direct neuron–cancer cell interactions but also to the influence of the nervous system on other cells of the local stromal, immune, and systemic tumor environment. However, rather than dissecting the specific molecular mechanisms mediating these effects, our study aims to demonstrate a functional connection between a negative mood–regulating neuronal circuit and tumor growth. The detailed mechanisms underlying how peripheral sympathetic innervation modulates breast cancer progression need further investigation.

In summary, our findings reveal a brain-tumor neural circuit that is activated by cancer-induced anxiety and controls tumor progression. These findings may lead to new therapeutic interventions for breast cancer.

## Methods

Additional methods are available in Supplemental Methods.

### Statistics.

Statistical significance was determined by 2-tailed, unpaired Student’s *t* test, 1-way ANOVA followed by Tukey post hoc test, or 2-way, repeated-measures ANOVA followed by a separate 1-way ANOVA or a 2-tailed, unpaired Student’s *t* test using SPSS software for Windows (version 25.0). In addition, 2-sided linear regression analysis was used to evaluate the correlation. A value of *P* value of less than 0.05 was considered statistically significant. All data in the figures are presented as the mean ± SEM.

### Study approval.

All animal experiments were approved by the Animal Care Committee of Army Medical University, in accordance with the principles outlined in the NIH’s *Guide for the Care and Use of Laboratory Animals* (National Academies Press, 2011).

### Data availability.

Raw data for this study are also available in the Supplemental Supporting Data Values file or from the corresponding author upon request.

## Author contributions

GYW and YZ initiated and designed the research. SYX, HZW, and GYW performed most of the experiments, analyzed the data, and prepared the figures. LMD, YXL, ZQW, XJY, YRW, PHC, SZY, and XWQ contributed to some of the experiments. YLY helped with figure preparation. WS assisted with flow cytometry. GYW, YZ, HZW, and SYX wrote the manuscript. All the authors commented on the manuscript.

## Supplementary Material

Supplemental data

Supporting data values

## Version 1 10/17/2023

In-Press Preview

## Version 2 12/15/2023

Electronic publication

## Version 3 08/26/2026

Correction to supporting data values file
