# Supplementary material for: A brain-tumor neural circuit controls breast cancer progression in mice
Source: J Clin Invest. 2023 Dec 15;133(24):e167725. doi: 10.1172/JCI167725 (PMC10721160; doi:10.1172/JCI167725)
Supplement: Supplemental data [file jci-133-167725-s146.pdf]

Supplementary Materials for

A brain–tumor neural circuit controls breast cancer progression in mice

Si-Yi Xiong[1,8], Hui-Zhong Wen[2,8], Li-Meng Dai[3], Yun-Xiao Lou[2], Zhao-Qun Wang[2], Yi-Lun Yi[4], Xiao-Jing Yan[5], Ya-Ran Wu[6], Wei Sun[7], Peng-Hui Chen[2], Si-Zhe Yang[1], Xiao-Wei Qi[1], Yi Zhang[1*], Guang-Yan Wu[4*]

[1]Breast and Thyroid Surgery, Southwest Hospital, Army Medical University, Chongqing 400038, China

[2]Department of Neurobiology, Chongqing Key Laboratory of Neurobiology, College of Basic Medical Sciences, Army Medical University, Chongqing 400038, China

[3]Department of Medical Genetics, College of Basic Medical Sciences, Army Medical University, Chongqing 400038, China

[4]Experimental Center of Basic Medicine, Chongqing Key Laboratory of Neurobiology, College of Basic Medical Sciences, Army Medical University, Chongqing 400038, China

[5]Department of Biochemistry and Molecular Biology, College of Basic Medical Sciences, Army Medical University, Chongqing 400038, China

[6]Department of Clinical Biochemistry, Faculty of Pharmacy and Laboratory Medicine, Army Medical University, Chongqing 400038, China

[7]Biomedical Analysis Center, Army Medical University, Chongqing 400038, China

[8]These authors contributed equally

[*]Corresponding author. wgy009@163.com (GYW); yzhang@tmmu.edu.cn (YZ)

The PDF file includes:

Methods

Supplemental Figures 1–26

Supplemental Table 1

Methods

Animals

Female BALB/c mice (8–10 weeks of age), FVB/NJGpt-Tg (MMTV-PyMT)/Gpt transgenic mice (MMTV-PyMT mice; 5–6 weeks of age; T004993, GemPharmatech, China), and FVB/NJ mice (13 weeks of age) were used in this study. These mice were housed in standard laboratory cages on a 12:12 h light/dark cycle (7:00 to 19:00, lights on) with ad libitum access to food and water.

Stereotaxic surgery and virus injection

Mice were anesthetized with 1.5–2% isoflurane and then fixed in a stereotaxic apparatus (Model 942, David Kopf Instruments, USA). The viruses were injected into the brain at a rate of 20 nl/min or into the tumor at a rate of 100 nl/min using a glass pipette with a tip diameter of approximately 20 μm, controlled by a programmable nanoliter injector (Nanoject III, Drummond Scientific, USA). Following the injection, the needle was left in place for an additional 5 min and then slowly withdrawn.

To selectively ablate CeM^{CRH} neurons, mice were microinjected with 50 nl of rAAV2/2-CRH-Cre (titer: 2.63× 10^{12} vg/mL) mixed with rAAV2/9-EF1α-Flex-taCasp3-TEVp (titer: 3.29 × 10^{12} vg/mL) or rAAV2/9-EF1α-DIO-EYFP (titer: 3.21 × 10^{12} vg/mL) as a control into the bilateral CeM (anteroposterior (AP): − 1.30 mm from bregma, mediolateral (ML): ± 2.50 mm, dorsoventral (DV): − 4.50 mm; Figure 4, B and C; Supplemental Figure 10B).

For pharmacogenetic inhibition of CeM^{CRH} neurons, mice were microinjected with 50 nl of rAAV2/2-CRH-Cre (titer: 2.63× 10^{12} vg/mL) mixed with rAAV2/9-EF1α-DIO-hM4Di-mCherry (titer: 2.73 × 10^{12} vg/mL) or rAAV2/9-EF1α-DIO-mCherry (titer: 3.63 × 10^{12} vg/mL) as a control into the bilateral CeM (Figure 5, B and C).

For pharmacogenetic activation of CeM^{CRH} neurons, mice were microinjected with 50 nl of rAAV2/2-CRH-Cre (titer: 2.63× 10^{12} vg/mL) mixed with rAAV2/9-EF1α-DIO-hM3Dq-mCherry (titer: 2.23 × 10^{12} vg/mL) or rAAV2/9-EF1α-DIO-mCherry (titer: 3.63 × 10^{12} vg/mL) as a control into the bilateral CeM (Figure 6, B and C).

For optogenetic inhibition of CeM^{CRH} neurons, mice were microinjected with 50 nl of rAAV2/2-CRH-Cre (titer: 2.63× 1012 vg/mL) mixed with rAAV2/9-EF1α-DIO-eNpHR3.0-mCherry (titer: 2.34

× 10^{12} vg/mL) or rAAV2/9-EF1α-DIO-mCherry (titer: 3.63 × 10^{12} vg/mL) as a control into the bilateral CeM. For optogenetic manipulation, a wireless optogenetic probe was implanted into the CeM and then secured to the skull using dental cement (Supplemental Figure 18, B and C).

For optogenetic activation of CeM^{CRH} neurons, mice were microinjected with 50 nl of rAAV2/2-CRH-Cre (titer: 2.63× 10^{12} vg/mL) mixed with rAAV2/9-EF1α-DIO-ChR2-mCherry (titer: 2.79 × 10^{12} vg/mL) or rAAV2/9-EF1α-DIO-mCherry (titer: 3.63 × 10^{12} vg/mL) as a control into the bilateral CeM. For optogenetic manipulation, a wireless optogenetic probe was implanted into the CeM and then secured to the skull using dental cement (Supplemental Figure 19, B and C).

For selective pharmacogenetic inhibition of the CeM^{CRH}→LPGi circuit (i.e., LPGi-projecting CRH neurons in the CeM), we injected rAAV2/retro-CRH-Cre (titer: 5.76× 10^{12} vg/mL) into the bilateral LPGi (AP: − 6.30 mm, ML: ± 1.20 mm, DV: − 5.80 mm) and rAAV2/9-EF1α-DIO-hM4Di-mCherry (titer: 2.25 × 10^{12} Vg/mL) or rAAV2/9-EF1α-DIO-mCherry (titer: 3.63 × 10^{12} vg/mL) as a control into the bilateral CeM (Figure 8, B and C) at a volume of 50 nl for each site.

For selective pharmacogenetic activation of the CeM^{CRH} → LPGi circuit, we injected rAAV2/retro-CRH-Cre (titer: 5.76× 10^{12} vg/mL) into the bilateral LPGi and rAAV2/9-EF1α-DIO-hM3Dq-mCherry (titer: 2.23 × 10^{12} vg/mL) or rAAV2/9-EF1α-DIO-mCherry (titer: 3.63 × 10^{12} vg/mL) as a control into the bilateral CeM (Figure 9, B and C) at a volume of 50 nl for each site.

For selective optogenetic inhibition of the CeM^{CRH}→LPGi circuit, we injected rAAV2/retro-CRH-Cre (titer: 5.76× 10^{12} vg/mL) into the bilateral LPGi and rAAV2/9-EF1α-DIO-eNpHR3.0-mCherry (titer: 2.34 × 10^{12} vg/mL) or rAAV2/9-EF1α-DIO-mCherry (titer: 3.63 × 10^{12} vg/mL) as a control into the bilateral CeM at a volume of 50 nl for each site. For optogenetic manipulation, a wireless optogenetic probe was implanted into the CeM and then secured to the skull using dental cement (Supplemental Figure 20, B and C).

For selective optogenetic activation of the CeM^{CRH}→LPGi circuit, we injected rAAV2/retro-CRH-Cre (titer: 5.76× 10^{12} vg/mL) into the bilateral LPGi and rAAV2/9-EF1α-DIO-ChR2-mCherry (titer: 2.79 × 10^{12} vg/mL) or rAAV2/9-EF1α-DIO-mCherry (titer: 3.63 × 10^{12} vg/mL) as a control into the bilateral CeM at a volume of 50 nl for each site. For optogenetic manipulation, a wireless optogenetic probe was implanted into the CeM and then secured to the skull using dental cement

(Supplemental Figure 21, B and C).

For selective optogenetic stimulation of CeM^{CRH} neurons and fiber photometry recording of the activity of sympathetic nerves distributed in the tumor stroma, we injected rAAV2/2-CRH-Cre (titer: 2.63×10^{12} vg/mL) mixed with rAAV2/9-EF1α-DIO-ChR2-mCherry (titer: 2.79×10^{12} vg/mL) into the bilateral CeM at a volume of 50 nl for each site and implanted optical fibers above the bilateral CeM (AP: − 1.30 mm, ML: ± 2.50 mm, DV: − 4.30 mm). Two weeks later, we also injected pLenti-CMV-$GRAB_{NE2h}$ (titer: 6.08×10^8 TU/mL, 1 μL) or pLenti-CMV-EGFP (titer: 8.75×10^8 TU/mL, 1 μL) virus into the tumor stroma of the above mice (Figure 3, F–H).

For selective optogenetic stimulation of CeM^{CRH}→LPGi circuits and fiber photometry recording of the activity of sympathetic nerves distributed in the tumor stroma, we injected rAAV2/retro-CRH-Cre (titer: 5.76×10^{12} vg/mL) into the bilateral LPGi and rAAV2/9-EF1α-DIO-ChR2-mCherry (titer: 2.79×10^{12} vg/mL) into the bilateral CeM at a volume of 50 nl for each site. We also implanted optical fibers above the bilateral CeM (AP: − 1.30 mm, ML: ± 2.50 mm, DV: − 4.30 mm). Two weeks later, we injected pLenti-CMV-$GRAB_{NE2h}$ (titer: 6.08×10^8 TU/mL, 1 μL) or pLenti-CMV-EGFP (titer: 8.75 $\times 10^8$ TU/mL, 1 μL) virus into the tumor stroma of the above mice (Figure 7, F–H).

For retrograde monosynaptic tracing, we injected rAAV2/8-Dbh-Cre (2.97×10^{12} vg/ml) together with AAV-helper viruses (rAAV2/9-EF1α-DIO-mCherry-TVA (2.92×10^{12} vg/ml) and rAAV2/9-EF1α-DIO-RVG (2.82×10^{12} vg/ml)) into the LPGi at a volume of 50 nl. Three weeks later, RV-EnvA-ΔG–EGFP (2.5×10^8 IFU/mL, 50 nl) was injected into the same LPGi. Seven days after the last injection, the mice were deeply anesthetized with isoflurane and transcardially perfused. The brains were removed from the skull and prepared for tracing the EGFP signal or performing immunofluorescence staining with CRH in the CeM (Figure 7, A–C).

For PRV retrograde polysynaptic tracing from the tumor or mammary gland, we injected 1 μL of PRV-CAG-EGFP (2.0×10^8 PFU/mL) into the 4T1 tumor stroma 7 days after tumor cell inoculation (Figure 1C), into the mammary gland of non-tumor bearing mice (Supplemental Figure 6 B), or into the spontaneous mammary tumor of MMTV-PyMT mice (Supplemental Figure 7 B). For histology, brains were collected 6 days after PRV injection.

These viruses were obtained from Obio Technology (Shanghai, China), BrainVTA (Wuhan, China) or Brain Case (ShenZhen, China). For all experiments, animals with incorrect injection sites were

excluded from further analysis. The efficacy of viruses in pharmacogenetic and optogenetic experiments has been confirmed in our previous works (1, 2).

Cell culture

The 4T1 breast cancer cell line (4T1-CMV-luc-Puro, HYC3496; 4T1, HYC3076) was obtained from Obio Technology (Shanghai, China). The cells were cultured in RPMI 1640 medium (11875093, Thermo Fisher Scientific) containing 10% FBS (10437036, Thermo Fisher Scientific) and supplemented with 1.0 μg/mL puromycin (A1113802; Thermo Fisher Scientific) in a humidified incubator at 37°C with 5% CO^2 (Forma CO^2 Incubator, Thermo Fisher Scientific). Transfected cells at the logarithmic growth stage were collected and diluted to the desired concentration with Matrigel (356234, BD Bioscience).

Breast tumor induction

4T1-luc or 4T1 breast cancer cells (0.2×10^6 cells per mouse, 100 μL) were subcutaneously injected into the mammary gland of BALB/c mice, and the control female mice were inoculated with equal Matrigel in the same site. Breast tumor sizes were measured every 7 days using a digital caliper, and the tumor volume was calculated as $(\text{length} \times \text{width}^2)/2$.

Bioluminescence imaging

The growth and metastasis of breast tumors were observed by an in vivo bioluminescence imaging system (AniView100, Guangzhou Biolight Biotechnology Co., Ltd.). Before observation, each mouse was injected (i.p., 150 mg/kg) with fluorescein substrate (D-Luciferin, Sodium Salt, 40901ES01, Yeasen, China). After anesthesia with 1.5–2% isoflurane, the mice were placed into an in vivo bioluminescence imaging system to obtain bioluminescence images and data. Data were analyzed using AniView software (Guangzhou Biolight Biotechnology Co., Ltd.).

Behavioral experiments

The anxiety-like behaviors of mice were evaluated with the light-dark box test (LDT), open field test (OFT), and elevated plus maze (EPM) test at 28 days after tumor cell inoculation. During the scoring process, the operator was blinded to the experimental groups.

Light-dark box test

The apparatus consists of a covered box (34 × 17 × 17 cm), which is equally divided into two compartments by a partition with a hole at floor level. The light compartment is brightly illuminated by white diodes (390 lux), and the dark compartment is completely dark. During the test, each mouse was placed into the light compartment and left to move freely between the light and dark compartments for 5 min. The transition behavior between the light and dark compartments was monitored with a digital camera during the experiment, and the data were acquired and analyzed by VisuTrack 2020 (Xinruan, Shanghai, China).

Open field test

The OFT was performed in a square chamber (50 × 50 cm) with 45 cm high walls. The floor of the chamber is evenly divided into sixteen squares; the central four squares are named the center zone, and the others are named peripheral zones. During the test, each mouse was placed in the center zone and then allowed to freely move for approximately 5 min. The motion trials were recorded by a video camera during the experiment, and the data were acquired and analyzed by VisuTrack 2020 (Xinruan, Shanghai, China).

Elevated plus maze test

The EPM is a crossed shape platform elevated 60 cm above the floor and consists of a central intersection platform (5 × 5 cm), two open arms (35 × 5 cm) and the two closed arms (35 × 5 × 16 cm). During the test, each mouse was placed on the central intersection platform and left to explore the maze for 10 min. The measures of the EPM test were recorded by video camera during the experiment, and the data were acquired and analyzed by VisuTrack 2020 (Xinruan, Shanghai, China). The anxiety index was calculated using the following equation: (1−((open arm time/total time on the maze) + (open arm entry/total entry)))/2.

Breast tumor procurement

After the behavioral experiments, the tumor-bearing mice were deeply anesthetized with isoflurane, and the primary breast tumor tissues were quickly removed and placed on ice. The breast

tumor tissues were washed with precooled phosphate-buffered saline (PBS, 0.01 M, pH = 7.4) to remove the residual blood, and then the weight of the tumors was measured as quickly as possible.

NE assessment

For the analysis of NE levels in breast tumor tissues in different groups, NE enzyme-linked immunosorbent assay (ELISA) kits (ml063805, Enzyme-linked Biotechnology Co., Ltd., Shanghai, China) were used. The tumor tissues (0.1 g) were weighed, 0.9 ml PBS (0.01 M, pH = 7.4) was added and then they were fully homogenized by a homogenizer. Next, the supernatants were carefully collected after centrifugation for 15 min at 2000 × g. Fifty microliters of different concentrations of the standard and 50 μl of different sample supernatants were added to the well according to the manufacturer's instructions. Then, 100 μL of HRP-conjugated reagent was added and incubated for 60 min at 37°C. The plates were washed with washing buffer 5 times, and the mixed substrate (100 μL) was added to each well. After incubation for 15 min at 37°C, 50 μL termination solutions were added to each well, and the optical density (OD) value was measured by a microplate reader (Imark, Bio-Rad, California, United States) at 450 nm 3 times.

Pharmacogenetic manipulations

For in vivo pharmacogenetic inhibition or activation of CeM^{CRH} neurons (Figures 5 and 6) or the CeM^{CRH}→LPGi circuit (Figures 8 and 9), tumor-bearing mice were raised on food with CNO (~5 mg/kg, assuming food consumption of 5 g per day per mouse) after cancer cell injection for 4 weeks.

Optogenetic manipulations

For in vivo wireless optogenetic inhibition of CeM^{CRH} neurons (Supplemental Figure 18) or the CeM^{CRH}→LPGi circuit (Supplemental Figure 20), tumor-bearing mice expressing eNpHR3.0 or mCherry received 60-min rest–stimulation cycles (5 min laser off–5 min LED on, repeated 6 times) of illumination (589 nm, continuous, ~2 mW) twice a day. The 589-nm wireless optogenetic probe was controlled by a wireless optogenetic controller (inper, China).

For in vivo wireless optogenetic activation of CeM^{CRH} neurons (Supplemental Figure 19) or the CeM^{CRH}→LPGi circuit (Supplemental Figure 21), tumor-bearing mice expressing ChR2 or mCherry

received 60-min rest–stimulation cycles (5 min laser off–5 min LED on, repeated 6 times) of illumination (470 nm, ~2 mW, 10 Hz, 15 ms) twice a day. The 470-nm wireless optogenetic probe was controlled by a wireless optogenetic controller (inper, China).

For optogenetic stimulation of CeM^{CRH} neurons (Figure 3, E–M) or the CeM^{CRH}→LPGi circuit (Figure 7, E–M), tumor-bearing mice expressing ChR2 received laser illumination (473 nm, ~5 mW, 20 Hz, 15-ms pulse duration, 20 epochs of 5 s, separated by an interval of 60–120 s) delivered from a 473-nm laser (BL473T3, SLOC, China)

Alprazolam treatment.

To reduce the anxiety of tumor-bearing mice, the anxiolytic drug alprazolam was administered (i.p., 0.3 mg/kg, twice a day). Control mice were similarly injected with vehicle (Figures, 10A and 11A).

Fiber photometry

The activity of sympathetic nerves distributed in the tumor stroma was measured by NE release. Fluorescence signals of the $GRAB_{NE2h}$ sensor or EGFP in the tumor tissue were recorded by a fiber photometry recording system (inper, China) during optogenetic stimulation of CeM^{CRH} neurons or the CeM^{CRH}→LPGi circuit. Fluorescence signal excitation was provided by a 488-nm laser. Moreover, a 405-nm laser was concurrently used to isolate other signals from the channel. The laser power was adjusted at the tip of the optical fiber to a low level of 20–40 μW to minimize bleaching of $GRAB_{NE2h}$ and EGFP.

The fiber photometry data were analyzed using built-in software. The fluorescence value during each optogenetic stimulation was derived. The change in the fluorescence values (ΔF/F) was calculated by (F-F0)/F0, where F refers to the fluorescence values at each time point (-2 to 6 s relative to the onset of the optogenetic stimulation), and F0 refers to the median of the fluorescence values during the baseline period (-2 to 0 s relative to the onset of the optogenetic stimulation). To visualize the fluorescence change, the Δ F/F values are presented with heatmaps or plots with shaded areas indicating SEM. To statistically quantify the change in fluorescence values across the optogenetic stimulation, the average amplitude of ΔF/F was defined as the average fluorescence amplitude change

from the baseline during the peak period (0 to 5 s relative to the onset of the optogenetic stimulation; Figure 3, E–M and Figure 7, E–M).

Cell viability assay

A CCK8 assay was performed according to the manufacturer's protocol (C0042, Beyotime, China). The 4T1 cells (2000 cells per well with 100 μL of medium) were plated in 96-well plates. At different time points, 10 μl of CCK8 solution was added to each well, and the wells were cultured at 37 °C in a humidified 5% CO2 atmosphere for 1 h. Then, the absorbance at 450 nm of each well was measured.

The Anaixin V-FITC/PI Cell Apoptosis Detection Kit (C1062M, Beyotime, China) was used for cell apoptosis detection. About 1 million cells were resuspended with cold D-PBS. Afterwards, the cells were suspended for 15 min at 37 °C in combined buffer supplemented with Annexin V-FITC and PI. Finally, these cells were analyzed using a Accuri C6 plus flow cytometer (BD Biosciences).

Isolation of lymphocytes and macrophages from spleens or tumor tissues

The fresh tumor tissues from 4T1 or PyMT tumor-bearing mice were cut into ~1 mm^3 pieces and digested with 25 ml of DMEM supplemented with 1 mg/ml collagenase IV (A004186, Sangon Biotech, China), 3% FBS, 0.1 mg /ml hyaluronidase (H8030, Solarbio, China) and 0.01 mg/ml DNase I (D8071, Solarbio, China) at 37 °C for 60 min. The dissociated cells were collected into a 15-ml tube and centrifuged at 500 × g for 5 min. The cells were resuspended in 5 ml of Red Blood Cell Lysis Buffer (R1010, Solarbio, China), kept still for 5 min at 37 °C, centrifuged at 500 × g for 5 min and washed with Buffer A (PBS with 2 mM EDTA, 0.5% FBS, and 25 mM HEPES) twice before filtration with a 70-μm filter. These cells were collected and resuspended in Buffer A for further analysis.

The fresh spleens from 4T1 or PyMT tumor-bearing mice were dissected and put in fresh Buffer A in a 60-mm dish. The spleen was gently rubbed between the two rough sides of frosted slides. The dissociated cells were filtered through a 70-μm filter, and then collected into a 15-ml tube and centrifuged at 500 × g for 5 min. The pellets were resuspended with 5ml Red Blood Cell Lysis Buffer for 5 min at 37 °C and centrifuged at 500 × g for 5 min. These cells were washed with Buffer A again and resuspended in an appropriate amount for further sorting of lymphocytes and macrophages.

Fluorescence-activated cell sorting (FACS)

The tumor tissue-derived cells and splenocytes in Buffer A were counted. An aliquot of cells was pelleted and resuspended in the Buffer A at 10^7 cells/mL. A total of 1×10^6 cells per sample were incubated with FVS780 according to the manufacturer's protocol (565388, BD Biosciences) for live/dead discrimination. For lymphocytes analysis, cells were stained with antibodies for 30 min at 4 °C. The antibodies included FITC anti-mouse CD45 antibody (103108, Biolegend), Brilliant Violet 510™ anti-mouse CD3 antibody (100234, Biolegend), PerCP/Cyanine5.5 anti-mouse CD4 antibody (100434, Biolegend), PE/Cyanine7 anti-mouse CD8a antibody (100722, Biolegend), BV650 Hamster anti-mouse CD279 antibody (PD-1; 744546, BD Biosciences), BV786 anti-mouse CD25 antibody (564023, BD Biosciences). Then, the antibodies of Brilliant Violet 421™ anti-mouse FOXP3 (126419, Biolegend) and Alexa Fluor® 647 anti-mouse IFN-γ (505814, Biolegend) were incubated according to the manufacturer's protocol of Transcription Factor Buffer Set (562574, BD Biosciences).

For macrophages analysis, cells were incubated with TruStain FcX™ (anti-mouse CD16/32) blocking antibody (101320, Biolegend) prior to staining with fluorochrome-conjugated antibodies for 15 min at 4 °C. The fluorochrome-conjugated antibodies included FITC anti-mouse CD45 antibody (103108, Biolegend), Brilliant Violet 421™ anti-mouse F4/80 antibody (123132, Biolegend), PerCP/Cyanine5.5 anti-mouse/human CD11b antibody (101228, Biolegend), PE anti-mouse CD86 antibody (105008, Biolegend), and APC anti-mouse CD206 antibody (141708, Biolegend).

The cells were resuspended in Buffer A for FACS analysis on a BD LSRFortessa™ Cell Analyzer (BD Biosciences) or CytoFLEX LX flow cytometer (Beckman Coulter). The $CD4^+$ T cells were assessed as $CD45+CD3^+CD4^+$ cells. The $CD8^+$ T cells were assessed as $CD45^+CD3^+CD8^+$ cells. T regulatory cells (Tregs) were assessed as $CD4^+CD25^+FOXP3^+$ cells. M1-like macrophages were assessed as $CD45^+CD11b^+F4/80^+CD86^+CD206^-$ cells. The M2-like macrophages were assessed as $CD45^+CD11b^+F4/80^+CD86^-CD206^+$ cells.

Immunofluorescence staining

Mice were deeply anesthetized with isoflurane and perfused intracardially with preheated (37°C) physiological saline and precooled (4°C) 4% paraformaldehyde (PFA). The brains or tumors were removed and stored in 4% PFA at 4°C for 24 h and then transferred to 20% sucrose solutions at 4°C for 48 h. The brains or tumors were sectioned at a thickness of 30 μm using a freezing microtome

(CM1900, Leica). Slices were blocked in solution containing 10% normal goat serum and 0.5% Triton X-100 for 30 min at 37°C. Next, the slices were incubated with the primary antibodies at 37°C for 1 h and then transferred to a slow shaker at 4°C overnight. On the following day, the slices were washed 3 × 5 min with 0.01 M PBS (pH 7.4) and then incubated with the corresponding fluorescent secondary antibodies at room temperature for 2 h. Slices were washed with PBS (5 min), incubated for 10 min with DAPI (1:2000, D9542, Sigma–Aldrich) and then subjected to 3 more wash steps for 5 min in PBS, followed by mounting and cover-slipping on microscope slides. The images were acquired using a scanning laser microscope (LSM 800, Carl Zeiss, Germany; or SpinSR, Olympus, Japan) or virtual slide system (VS200, Olympus, Japan). Details of the antibody information are presented in Supplemental Table 1.

TUNEL assay

To examine tumor cell apoptosis, the terminal deoxynucleotidyl transferase – mediated deoxyuridine triphosphate nick end (TUNEL) assay was performed. Briefly, after washing with PBS, the tumor slices were permeabilized with 0.5% Triton X-100 for 30 min. Then, the slices were washed in PBS and incubated with a TUNEL reagent containing FITC fluorescein-dUTP for 60 min. After incubation, the slices were also stained with DAPI (1:2000) for 10 min to label the cell nucleus. All slices were mounted in pre-cleaned slides with antifade mounting medium, then imaged with a scanning laser microscope (SpinSR, Olympus).

Histology

To examine the expression of viruses and the placement of the optical fiber and wireless optogenetic probe, mice were deeply anesthetized with isoflurane and transcardially perfused with preheated (37°C) physiological saline and precooled (4°C) 4% PFA. The brains or tumors were removed and stored in 4% PFA at 4°C for 24 h and then transferred to 20% sucrose solutions at 4°C for 48 h. The brains or tumors were sectioned at a thickness of 30 μm using a freezing microtome (CM1900, Leica). Slices were washed with PBS (5 min), incubated for 10 min with DAPI (1:2000) and then subjected to 3 more wash steps for 5 min in PBS, followed by mounting and cover slipping on microscope slides. The virus expression and the placement of the optical fiber and wireless

optogenetic probe were analyzed using an Olympus VS200 virtual slide system (Japan) or a Carl Zeiss LSM 800 scanning laser microscope (Germany).

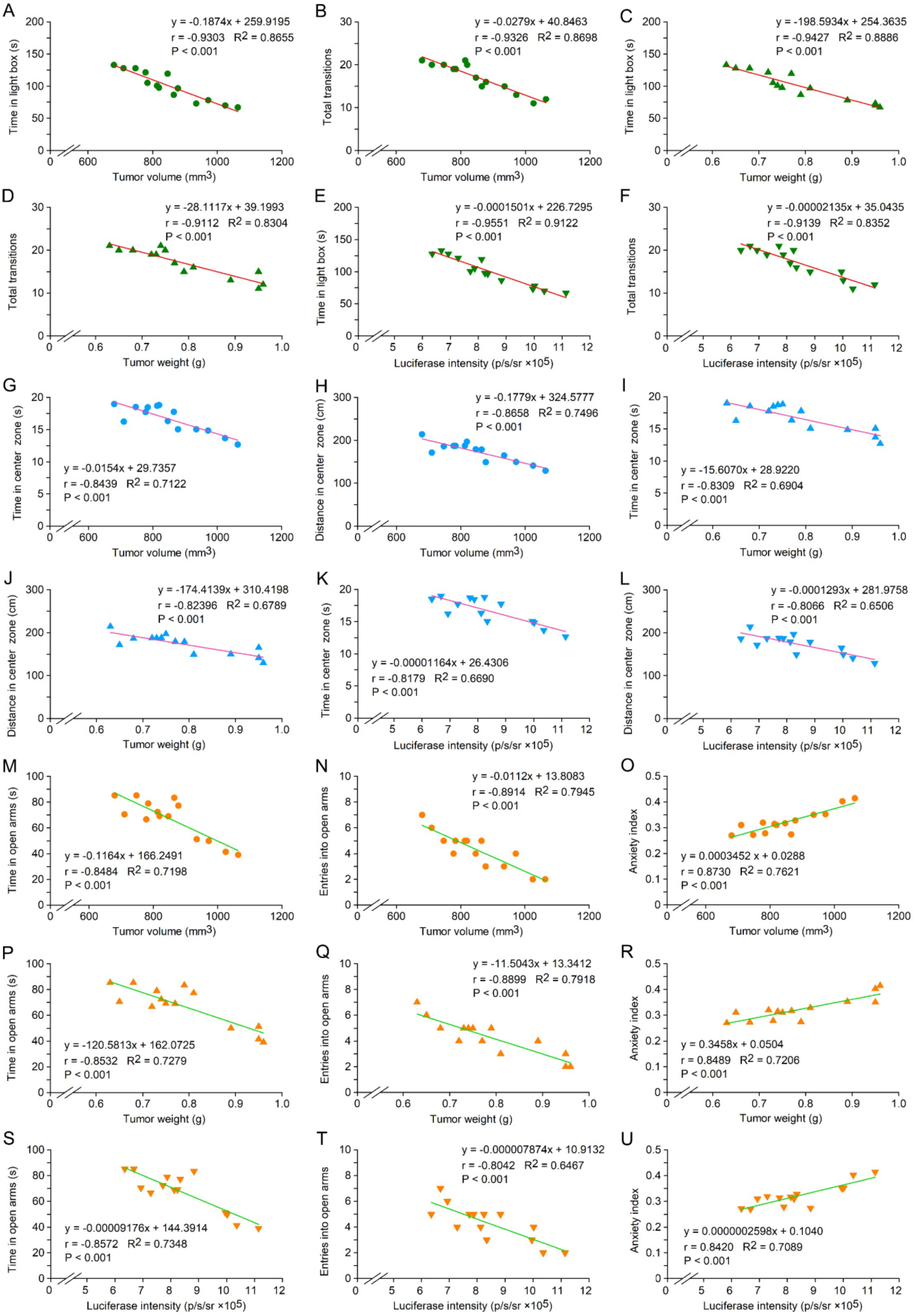
A
y = -0.1874x + 259.9195
r = -0.9303 R2 = 0.8655
P < 0.001
Time in light box (s)
Tumor volume (mm3)
B
y = -0.0279x + 40.8463
r = -0.9326 R2 = 0.8698
P < 0.001
Total transitions
Tumor volume (mm3)
C
y = -198.5934x + 254.3635
r = -0.9427 R2 = 0.8886
P < 0.001
Time in light box (s)
Tumor weight (g)
D
y = -28.1117x + 39.1993
r = -0.9112 R2 = 0.8304
P < 0.001
Total transitions
Tumor weight (g)
E
y = -0.0001501x + 226.7295
r = -0.9551 R2 = 0.9122
P < 0.001
Time in light box (s)
Luciferase intensity (p/s/sr ×105)
F
y = -0.00002135x + 35.0435
r = -0.9139 R2 = 0.8352
P < 0.001
Total transitions
Luciferase intensity (p/s/sr ×105)
G
y = -0.0154x + 29.7357
r = -0.8439 R2 = 0.7122
P < 0.001
Time in center zone (s)
Tumor volume (mm3)
H
y = -0.1779x + 324.5777
r = -0.8658 R2 = 0.7496
P < 0.001
Distance in center zone (cm)
Tumor volume (mm3)
I
y = -15.6070x + 28.9220
r = -0.8309 R2 = 0.6904
P < 0.001
Time in center zone (s)
Tumor weight (g)
J
y = -174.4139x + 310.4198
r = -0.82396 R2 = 0.6789
P < 0.001
Distance in center zone (cm)
Tumor weight (g)
K
y = -0.00001164x + 26.4306
r = -0.8179 R2 = 0.6690
P < 0.001
Time in center zone (s)
Luciferase intensity (p/s/sr ×105)
L
y = -0.0001293x + 281.9758
r = -0.8066 R2 = 0.6506
P < 0.001
Distance in center zone (cm)
Luciferase intensity (p/s/sr ×105)
M
y = -0.1164x + 166.2491
r = -0.8484 R2 = 0.7198
P < 0.001
Time in open arms (s)
Tumor volume (mm3)
N
y = -0.0112x + 13.8083
r = -0.8914 R2 = 0.7945
P < 0.001
Entries into open arms
Tumor volume (mm3)
O
y = 0.0003452 x + 0.0288
r = 0.8730 R2 = 0.7621
P < 0.001
Anxiety index
Tumor volume (mm3)
P
y = -120.5813x + 162.0725
r = -0.8532 R2 = 0.7279
P < 0.001
Time in open arms (s)
Tumor weight (g)
Q
y = -11.5043x + 13.3412
r = -0.8899 R2 = 0.7918
P < 0.001
Entries into open arms
Tumor weight (g)
R
y = 0.3458x + 0.0504
r = 0.8489 R2 = 0.7206
P < 0.001
Anxiety index
Tumor weight (g)
S
y = -0.00009176x + 144.3914
r = -0.8572 R2 = 0.7348
P < 0.001
Time in open arms (s)
Luciferase intensity (p/s/sr ×105)
T
y = -0.000007874x + 10.9132
r = -0.8042 R2 = 0.6467
P < 0.001
Entries into open arms
Luciferase intensity (p/s/sr ×105)
U
y = 0.0000002598x + 0.1040
r = 0.8420 R2 = 0.7089
P < 0.001
Anxiety index
Luciferase intensity (p/s/sr ×105)

Supplemental Figure 1. A positive and significant correlation was found between tumor growth and cancer-induced anxiety-like behavior in mice 4 weeks after 4T1-luc cells inoculation.

(A–F) Quantitative analysis of the correlation between tumor volume and time in the light box (A), tumor volume and total transitions (B), tumor weight and time in the light box (C), tumor weight and total transitions (D), tumor luciferase intensity and time in the light box (E), tumor luciferase intensity and total transitions (F). Related to Figure 1, G–I.

(G–L) Quantitative analysis of the correlation between tumor volume and time in the center zone (G), tumor volume and distance traveled in the center zone (H), tumor weight and time in the center zone (I), tumor weight and distance traveled in the center zone (J), tumor luciferase intensity and time in the center zone (K), tumor luciferase intensity and distance traveled in the center zone (L). Related to Figure 1, J–L.

(M–U) Quantitative analysis of the correlation between tumor volume and time in the open arms (M), tumor volume and entries into open arms (N), tumor volume and anxiety index (O), tumor weight and time in the open arms (P), tumor weight and entries into open arms (Q), tumor weight and anxiety index (R), tumor luciferase intensity and time in the open arms (S), tumor luciferase intensity and entries into open arms (T), tumor luciferase intensity and anxiety index (U). Related to Figure 1, M–P.

Two-sided linear regression analysis.

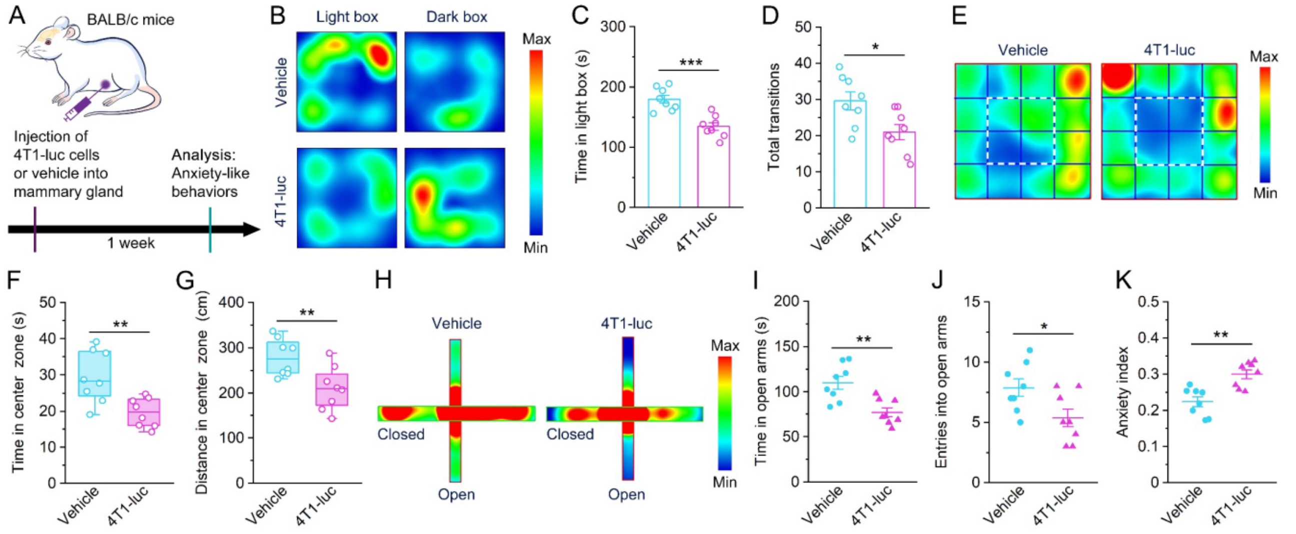

Supplemental Figure 2. 4T1 tumor-bearing mice showing serious anxiety at the early stage of progression.

(A) Schematic of the experimental design.

(B–D) In the light–dark box test, representative heatmaps (B), and quantitative summary of time spent in light box (C) and the total number of transitions (D) in the Vehicle (n = 8) and 4T1-luc (n = 8) groups.

(E–G) In the open field test, representative heatmaps (E), and quantification of the time spent in the center zone (F) and the distance traveled in the center zone (G) in the Vehicle (n = 8) and 4T1-luc (n = 8) groups.

(H–K) In the elevated plus maze test, representative heatmaps (H), and quantification of the time spent in the open arms (I), entries into open arms (J), and the anxiety index (K) in the Vehicle (n = 8) and 4T1-luc (n = 8) groups.

Data are presented as means ± SEM, except in box plot (F, G), in which the centerline indicates the median, box edges represent the first and third quartiles and whiskers denote minimal and maximal values. $^{*}P < 0.05$, $^{**}P < 0.01$, $^{***}P < 0.001$, two-tailed unpaired Student's t test (C, D, F, G, I–K).

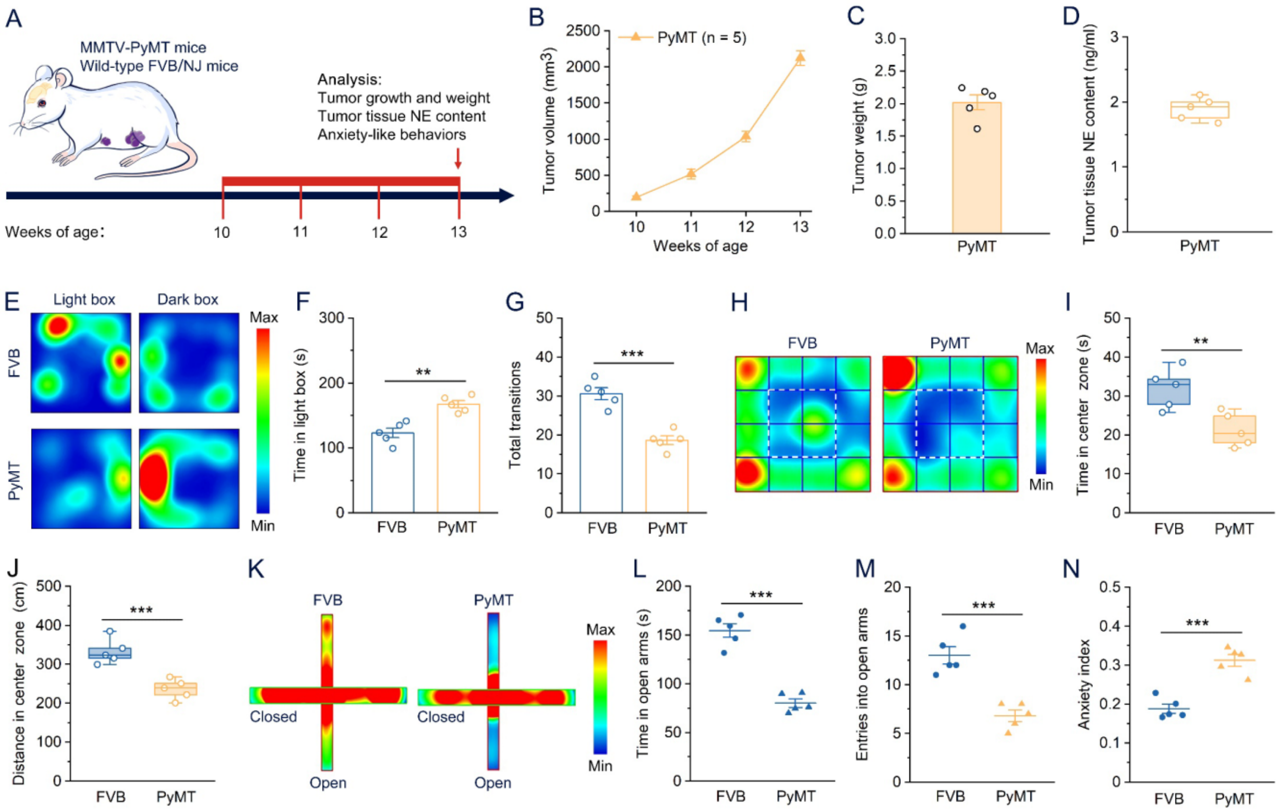

Supplemental Figure 3. PyMT tumor-bearing mice showing obvious anxiety.

(A) Schematic of the experimental design.

(B) Tumor growth curve of MMTV-PyMT mice (n = 5).

(C) The tumor weight of MMTV-PyMT mice at 13 weeks of age (n = 5).

(D) The NE content of tumor tissue from MMTV-PyMT mice at 13 weeks of age (n = 5).

(E–G) In the light–dark box test, representative heatmaps (E), and quantitative summary of time spent in light box (F) and the total number of transitions (G) in MMTV-PyMT (n = 5) and wild-type FVB/NJ (n = 5) mice.

(H–J) In the open field test, representative heatmaps (H), and quantification of the time spent in the center zone (I) and the distance traveled in the center zone (J) in MMTV-PyMT (n = 5) and wild-type FVB/NJ (n = 5) mice.

(K–N) In the elevated plus maze test, representative heatmaps (K), and quantification of the time spent in the open arms (L), entries into open arms (M), and the anxiety index (N) in MMTV-PyMT (n = 5) and wild-type FVB/NJ (n = 5) mice.

Data are presented as means ± SEM, except in box plot (I, J), in which the centerline indicates the median, box edges represent the first and third quartiles and whiskers denote minimal and maximal values. **P < 0.01, ***P < 0.001, two-tailed unpaired Student's t test (F, G, I, J, L–N).

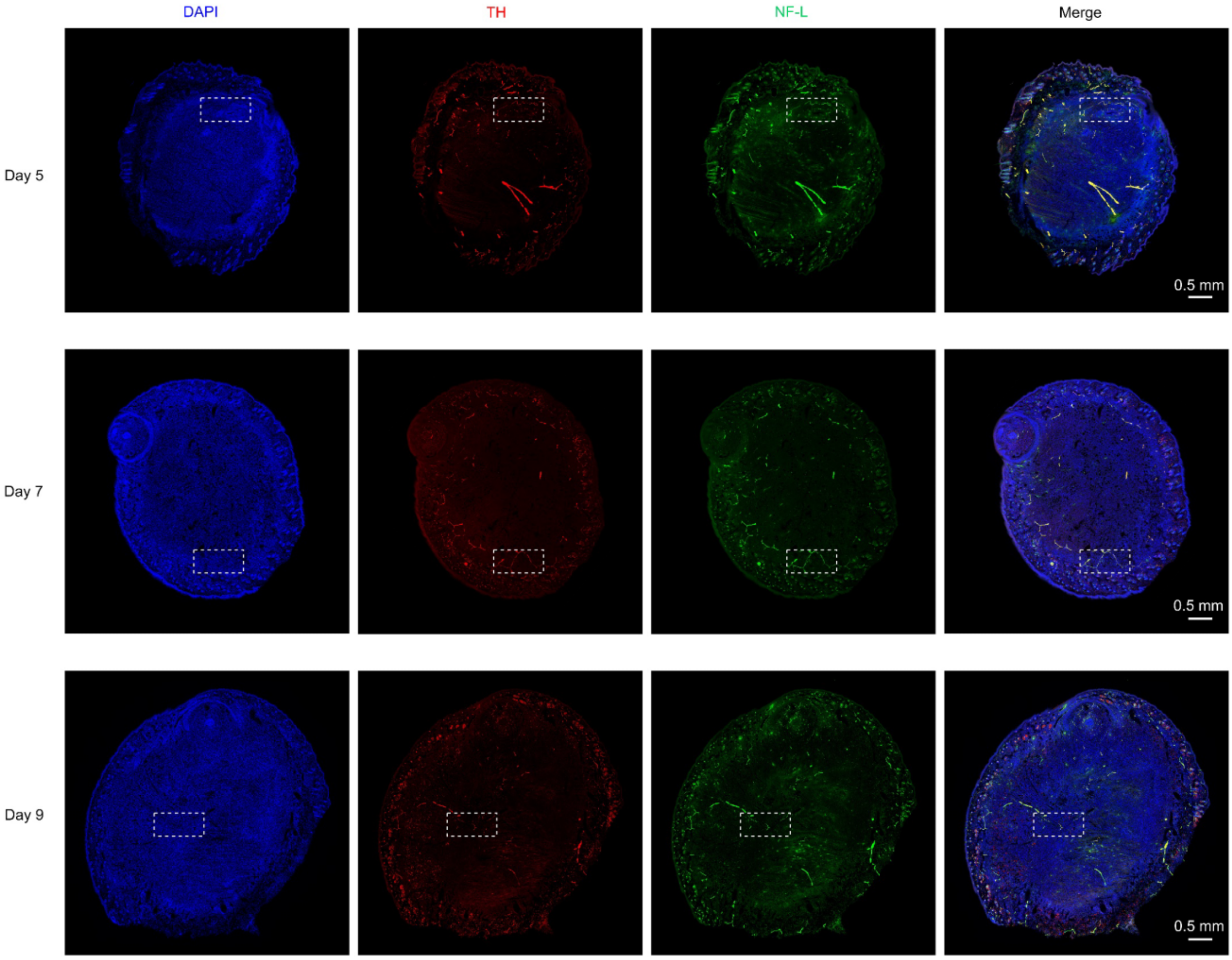

Supplemental Figure 4. Newly formed sympathetic nerves innervating 4T1 tumor. Related to Figure 2, A–C.

Representative images of tumor sections showing immunofluorescence staining of TH and NF-L 5 (upper), 7 (middle) and 9 (lower) days after 4T1 cells inoculation (n = 4 for each group). The boxed areas with higher magnification are shown in Figure 2B.

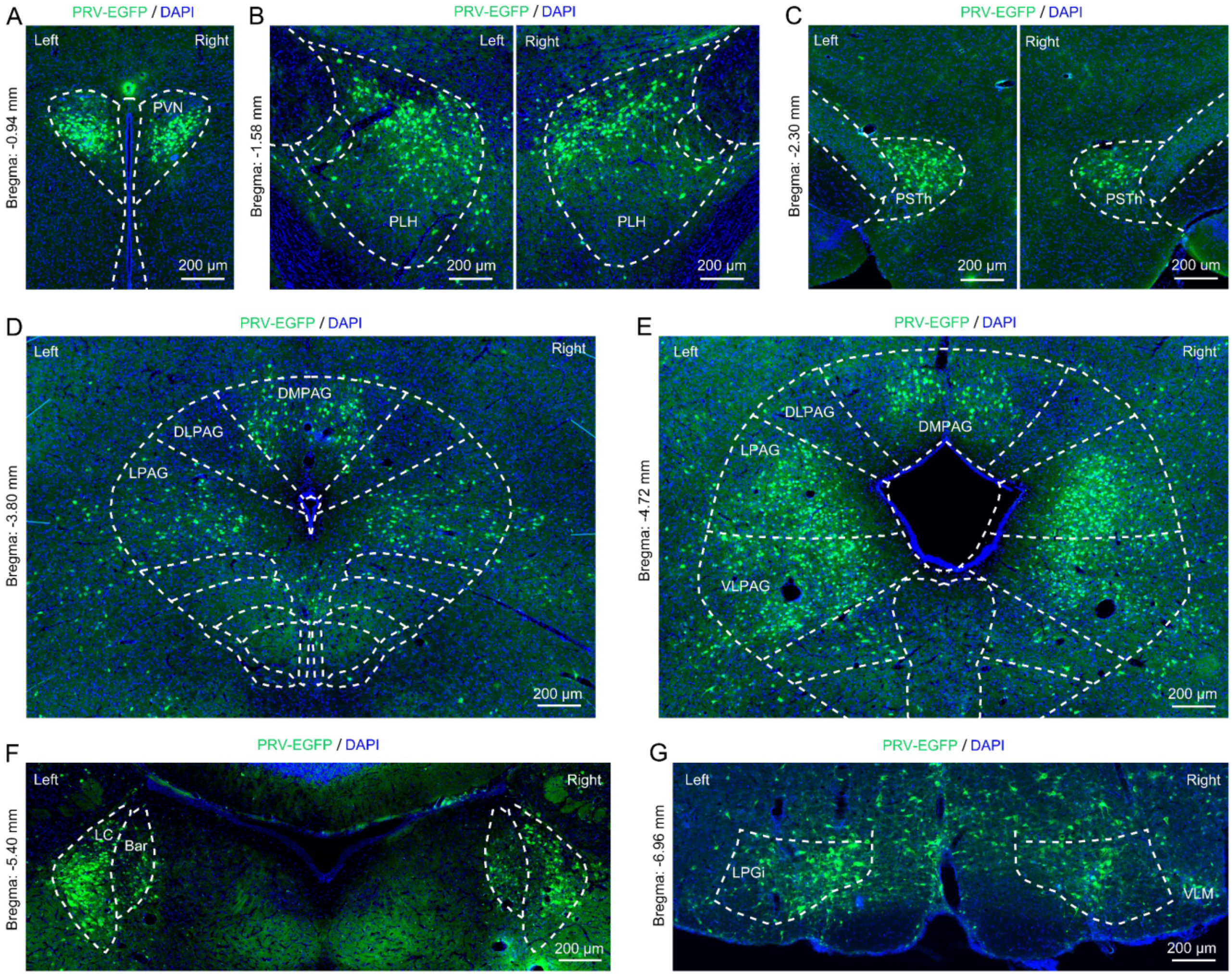

Supplemental Figure 5. Retrograde PRV tracing from the newly formed sympathetic innervation of 4T1 tumor. Related to Figure 2, D–J.

(A–G) Selected central nervous system regions that were infected by PRV.

Bar, Barrington’s nucleus; DLPAG, dorsolateral periaqueductal gray; DMPAG, dorsomedial periaqueductal gray; LC, locus coeruleus; LPAG, lateral periaqueductal gray; LPGi, lateral paragigantocellular nucleus; PLH, peduncular part of lateral hypothalamus; PSTh, parasubthalamic nucleus; PVN, paraventricular nucleus; VLM, ventrolateral medulla; VLPAG, ventrolateral periaqueductal gray.

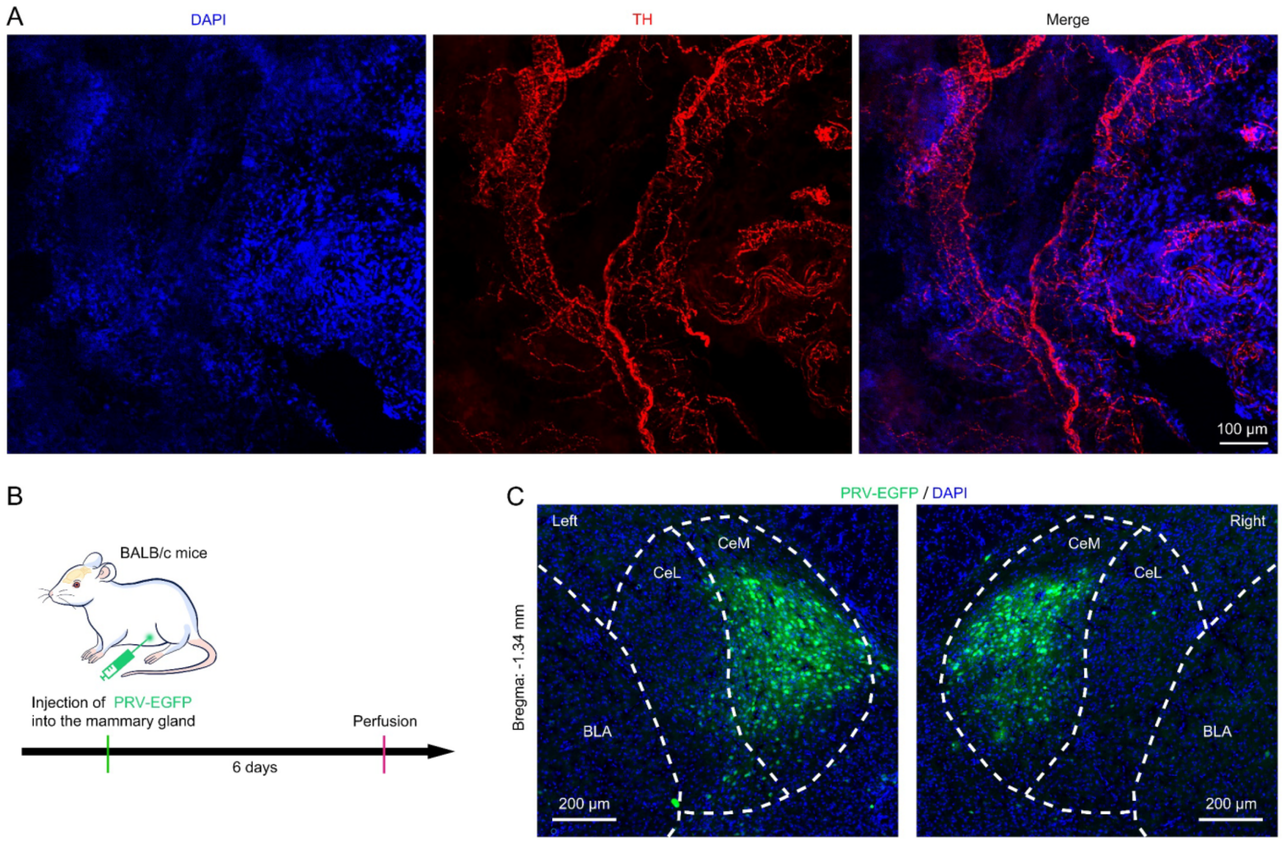

Supplemental Figure 6. The local sympathetic innervation in the mammary gland connects the CeM.

(A) Representative images showing high density TH^{+} sympathetic innervation in the mammary gland of wild-type BALB/c mice (non-tumor bearing mice).

(B) Experimental scheme depicting injection of neurotropic retrograde trans-polysynaptic pseudorabies virus PRV-EGFP into the mammary gland.

(C) Representative images of PRV^{+} neurons in CeM from the wild-type BALB/c mice 6 days after PRV-EGFP injection into the mammary gland.

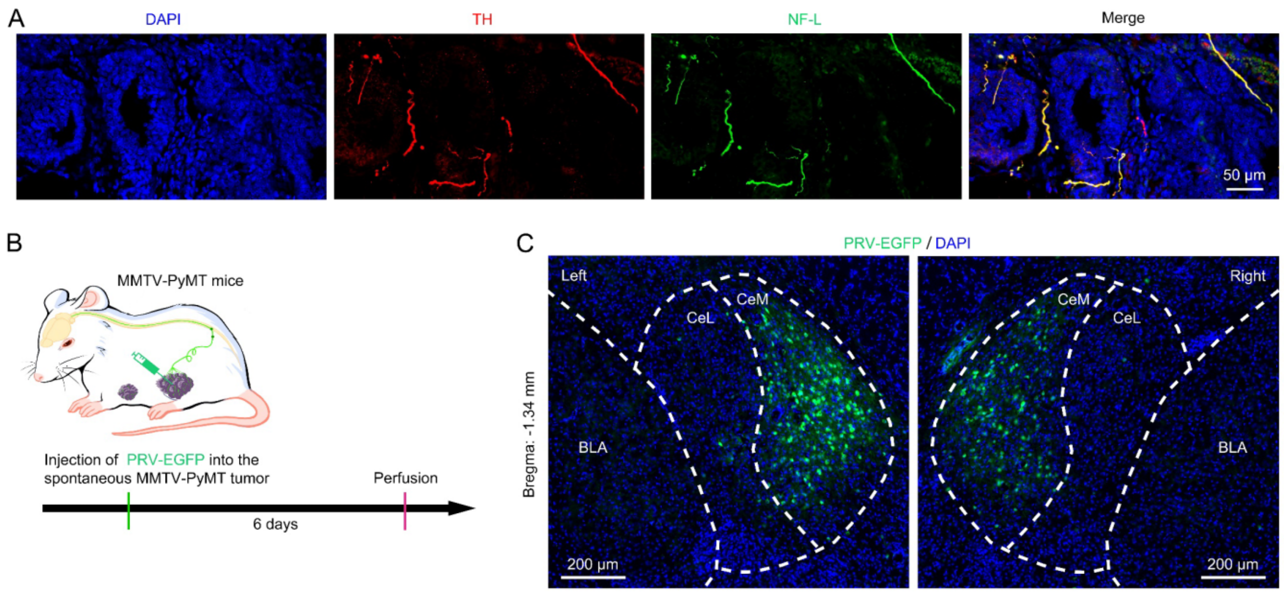

Supplemental Figure 7. The local sympathetic innervation of spontaneous mammary tumor connects the CeM in MMTV-PyMT mice.

(A) Representative images showing TH^+ sympathetic innervation of the spontaneous mammary tumor in MMTV-PyMT mice at 10 weeks of age.

(B) Experimental scheme depicting injection of neurotropic retrograde trans-polysynaptic pseudorabies virus PRV-EGFP into the spontaneous mammary tumor of MMTV-PyMT mice at 10 weeks of age.

(C) Representative images of PRV^+ neurons in CeM from the MMTV-PyMT mice 6 days after PRV-EGFP injection into the spontaneous mammary tumor.

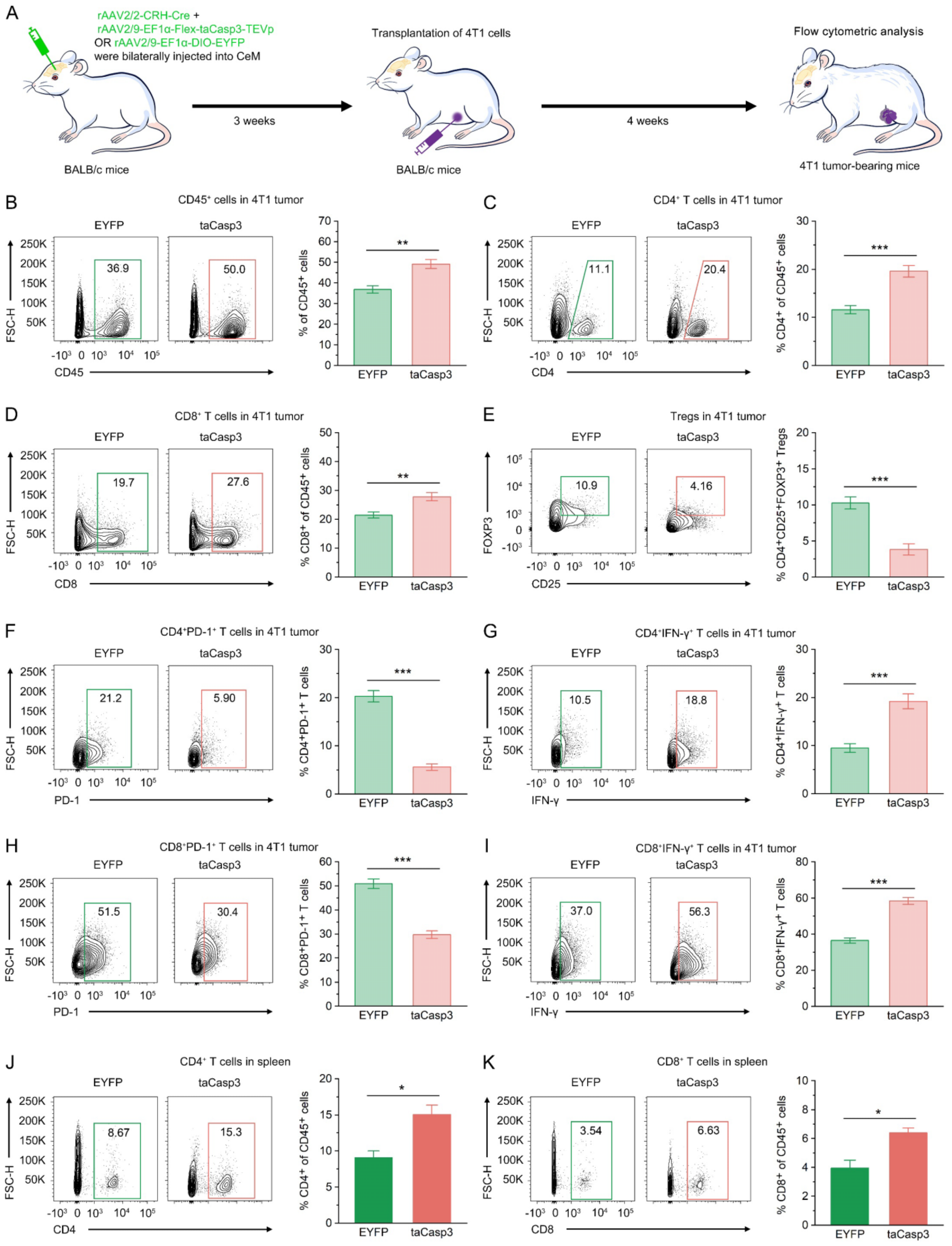

Supplemental Figure 8. The ablation of CeM^{CRH} neurons significantly enhances the antitumor immunity in 4T1 tumor-bearing mice. Related to Figure 4.

(A) Schematic of the experimental design.

(B) Representative flow cytometric plots and quantification of infiltrated $CD45^+$ cells in 4T1 tumor of the EYFP and taCasp3 groups.

(C, D) Representative flow cytometric plots and quantification of infiltrated $CD4^+$ T cells (C) and $CD8^+$ T cells (D) in 4T1 tumor of the EYFP and taCasp3 groups.

(E) Representative flow cytometric plots and quantification of infiltrated Tregs ($CD4^+CD25^+FOXP3^+$ T regulatory cells) in 4T1 tumor of the EYFP and taCasp3 groups.

(F, G) Representative flow cytometric plots and quantification of infiltrated $CD4^+PD\text{-}1^+$ T cells (F) and $CD4^+IFN\text{-}\gamma^+$ T cells (G) in 4T1 tumor of the EYFP and taCasp3 groups.

(H, I) Representative flow cytometric plots and quantification of infiltrated $CD8^+PD\text{-}1^+$ T cells (H) and $CD8^+IFN\text{-}\gamma^+$ T cells (I) in 4T1 tumor of the EYFP and taCasp3 groups.

(J, K) Representative flow cytometric plots and quantification of infiltrated $CD4^+$ T cells (J) and $CD8^+$ T cells (K) in spleen of the EYFP and taCasp3 groups.

n = 5 for each group. Data are presented as means ± SEM. *$P < 0.05$, **$P < 0.01$, ***$P < 0.001$, two-tailed unpaired Student’s t test.

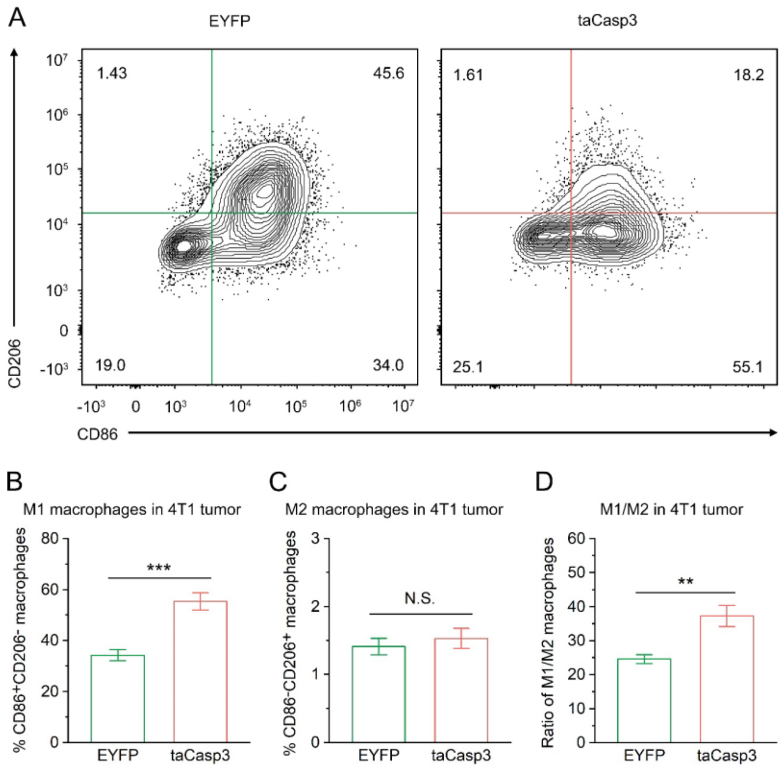

Supplemental Figure 9. The ablation of CeM^{CRH} neurons significantly increases M1 macrophages in tumor of 4T1 tumor-bearing mice. Related to Figure 4.

(A) Representative flow cytometry analysis of macrophages stained with CD86 and CD206 in 4T1 tumor.

(B, C) Quantification of M1 macrophages population (CD11b^{+}F4/80^{+}CD86^{+}CD206^{-}) and M2 macrophages population (CD11b^{+}F4/80^{+}CD86^{-}CD206^{+}) in 4T1 tumor of the EYFP and taCasp3 groups.

(D) The ratio of M1/M2 macrophage in 4T1 tumor of the EYFP and taCasp3 groups.

n = 5 for each group. Data are presented as means ± SEM. N.S., not significant, **$P < 0.01$, ***$P < 0.001$, two-tailed unpaired Student’s t test.

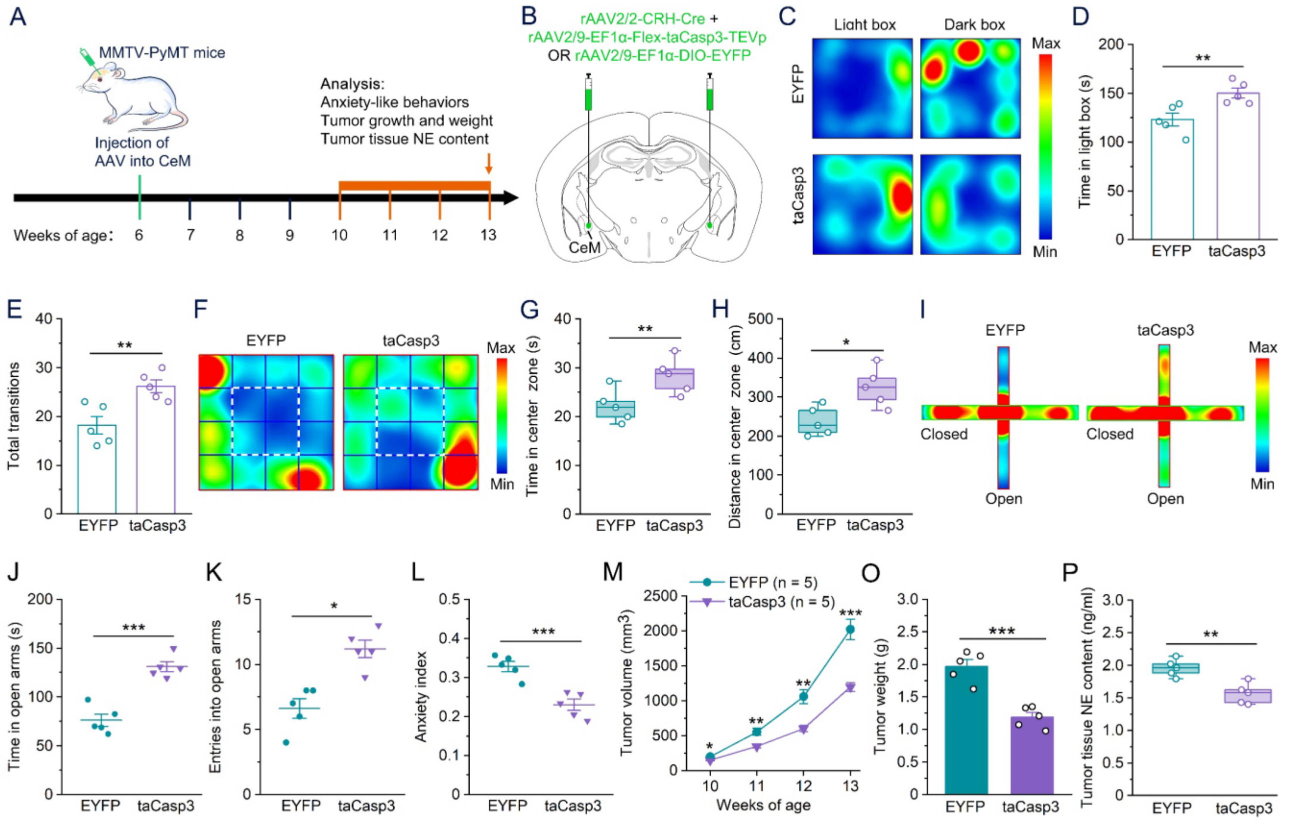

Supplemental Figure 10. The ablation of CeM^{CRH} neurons significantly reduces cancer-induced anxiety and suppresses tumor progression in MMTV-PyMT mice.

(A) Schematic of the experimental design.

(B) Schematic showing bilateral injections of rAAV2/2-CRH-Cre together with rAAV2/9-EF1α-Flex-taCasp3-TEVp or rAAV2/9-EF1α-DIO-EYFP into the CeM of MMTV-PyMT mice at 6 weeks of age.

(C–E) In the light–dark box test, representative heatmaps (C) and quantification of time spent in light box (D), and the total number of transitions (E) in the EYFP (n = 5) and taCasp3 (n = 5) groups.

(F–H) In the open field test, representative heatmaps (F) and quantification of the time spent in the center zone (G), and the distance traveled in the center zone (H) in the EYFP (n = 5) and taCasp3 (n = 5) groups.

(I–L) In the elevated plus maze test, representative heatmaps (I) and quantification of the time spent in the open arms (J), entries into open arms (K), and the anxiety index (L) in the EYFP (n = 5) and taCasp3 (n = 5) groups.

(M) The ablation of CeM^{CRH} neurons significantly slows PyMT tumor growth.

(O) The ablation of CeM^{CRH} neurons significantly reduces PyMT tumor weight.

(P) The ablation of CeM^{CRH} neurons significantly decreases NE content of PyMT tumor tissue (n = 5

for each group).

Data are presented as means ± SEM, except in box plots (G, H, P), in which centerlines indicate medians, box edges represent the first and third quartiles and whiskers denote minimal and maximal values. *$P < 0.05$, **$P < 0.01$, ***$P < 0.001$, two-way repeated-measures ANOVA followed by separate one-way ANOVA (M), two-tailed unpaired Student's t test (D, E, G, H, J–L, O, P).

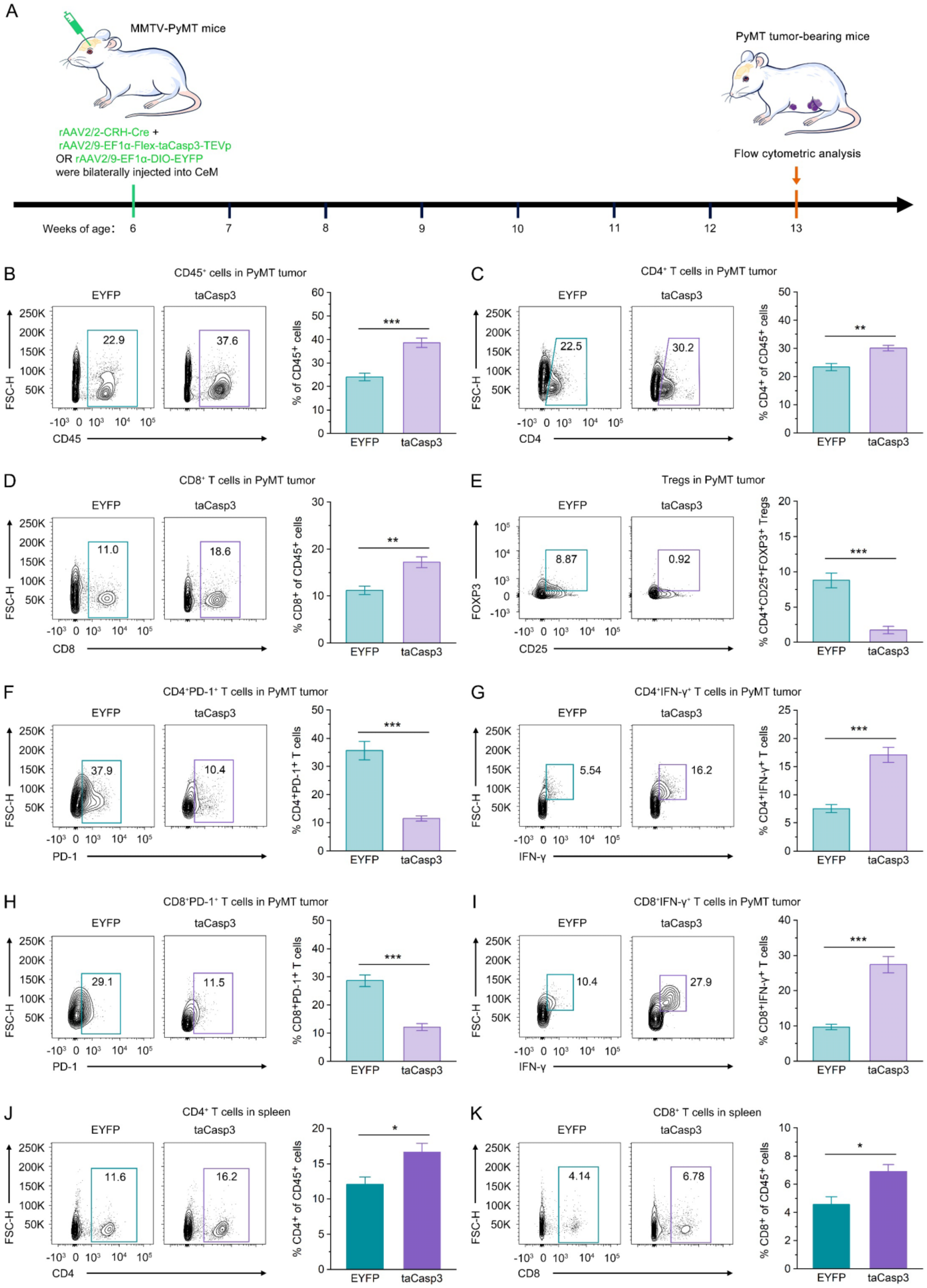
A
MMTV-PyMT mice
rAAV2/2-CRH-Cre +
rAAV2/9-EF1α-Flex-taCasp3-TEVp
OR rAAV2/9-EF1α-DIO-EYFP
were bilaterally injected into CeM
PyMT tumor-bearing mice
Flow cytometric analysis
Weeks of age: 6 7 8 9 10 11 12 13
B
CD45⁺ cells in PyMT tumor
EYFP taCasp3
22.9 37.6
% of CD45⁺ cells
C
CD4⁺ T cells in PyMT tumor
22.5 30.2
% CD4⁺ of CD45⁺ cells
D
CD8⁺ T cells in PyMT tumor
11.0 18.6
% CD8⁺ of CD45⁺ cells
E
Tregs in PyMT tumor
8.87 0.92
% CD4⁺CD25⁺FOXP3⁺ Tregs
F
CD4⁺PD-1⁺ T cells in PyMT tumor
37.9 10.4
% CD4⁺PD-1⁺ T cells
G
CD4⁺IFN-γ⁺ T cells in PyMT tumor
5.54 16.2
% CD4⁺IFN-γ⁺ T cells
H
CD8⁺PD-1⁺ T cells in PyMT tumor
29.1 11.5
% CD8⁺PD-1⁺ T cells
I
CD8⁺IFN-γ⁺ T cells in PyMT tumor
10.4 27.9
% CD8⁺IFN-γ⁺ T cells
J
CD4⁺ T cells in spleen
11.6 16.2
% CD4⁺ of CD45⁺ cells
K
CD8⁺ T cells in spleen
4.14 6.78
% CD8⁺ of CD45⁺ cells
FSC-H
CD45 CD4 CD8 CD25 FOXP3 PD-1 IFN-γ

Supplemental Figure 11. The ablation of CeM^{CRH} neurons significantly enhances the antitumor immunity in the PyMT tumor-bearing mice. Related to Supplemental Figure 10.

(A) Schematic of the experimental design.

(B) Representative flow cytometric plots and quantification of infiltrated $CD45^+$ cells in PyMT tumor of the EYFP and taCasp3 groups.

(C, D) Representative flow cytometric plots and quantification of infiltrated $CD4^+$ T cells (C) and $CD8^+$ T cells (D) in PyMT tumor of the EYFP and taCasp3 groups.

(E) Representative flow cytometric plots and quantification of infiltrated Tregs ($CD4^+CD25^+FOXP3^+$ T regulatory cells) in PyMT tumor of the EYFP and taCasp3 groups.

(F, G) Representative flow cytometric plots and quantification of infiltrated $CD4^+PD\text{-}1^+$ T cells (F) and $CD4^+IFN\text{-}\gamma^+$ T cells (G) in PyMT tumor of the EYFP and taCasp3 groups.

(H, I) Representative flow cytometric plots and quantification of infiltrated $CD8^+PD\text{-}1^+$ T cells (H) and $CD8^+IFN\text{-}\gamma^+$ T cells (I) in PyMT tumor of the EYFP and taCasp3 groups.

(J, K) Representative flow cytometric plots and quantification of infiltrated $CD4^+$ T cells (J) and $CD8^+$ T cells (K) in spleen of the MMTV-PyMT mice in the EYFP and taCasp3 groups.

n = 5 for each group. Data are presented as means ± SEM. *$P < 0.05$, **$P < 0.01$, ***$P < 0.001$, two-tailed unpaired Student’s t test.

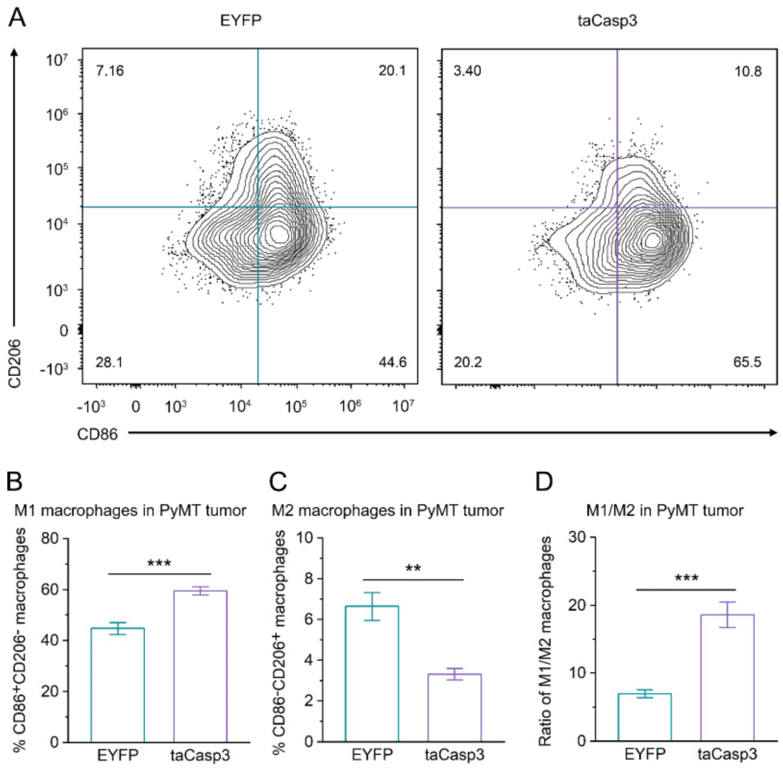

Supplemental Figure 12. The ablation of CeM^{CRH} neurons significantly increases M1 macrophages and decreases M2 macrophages in tumor of PyMT tumor-bearing mice. Related to Supplemental Figure 10.

(A) Representative flow cytometry analysis of macrophages stained with CD86 and CD206 in PyMT tumor.

(B, C) Quantification of M1 macrophages population ($CD11b^+F4/80^+CD86^+CD206^-$) and M2 macrophages population ($CD11b^+F4/80^+CD86^-CD206^+$) in PyMT tumor of the EYFP and taCasp3 groups.

(D) The ratio of M1/M2 macrophage in PyMT tumor of the EYFP and taCasp3 groups.

n = 5 for each group. Data are presented as means ± SEM. **P < 0.01, ***P < 0.001, two-tailed unpaired Student's t test.

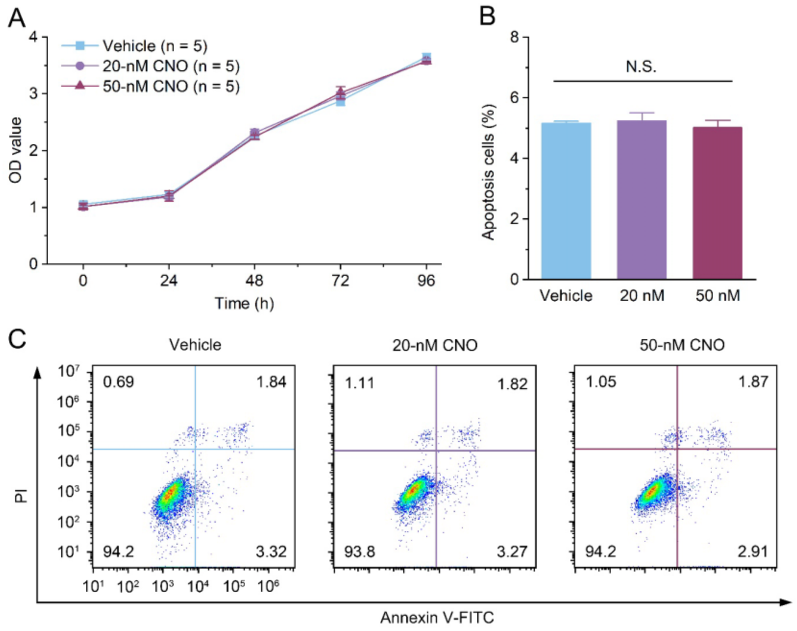

Supplemental Figure 13. CNO has no significant effect on proliferation and apoptosis of 4T1 breast cancer cells in vitro. Related to Figures 5 and 6.

(A) CNO administration has no significant effect on the proliferation of 4T1 cells in vitro. CCK8 assay was performed to determine the cell growth of 4T1 cells at consecutive time points with alprazolam.

(B and C) CNO administration has no significant effect on the apoptosis of 4T1 cells in vitro. After 48 hours of CNO administration, the apoptosis rate of 4T1 cells was determined by flow cytometry analysis (n = 4 for each group).

N.S., not significant, two-way repeated-measures ANOVA followed by separate one-way ANOVA (A), one-way ANOVA followed by Tukey post hoc test (B).

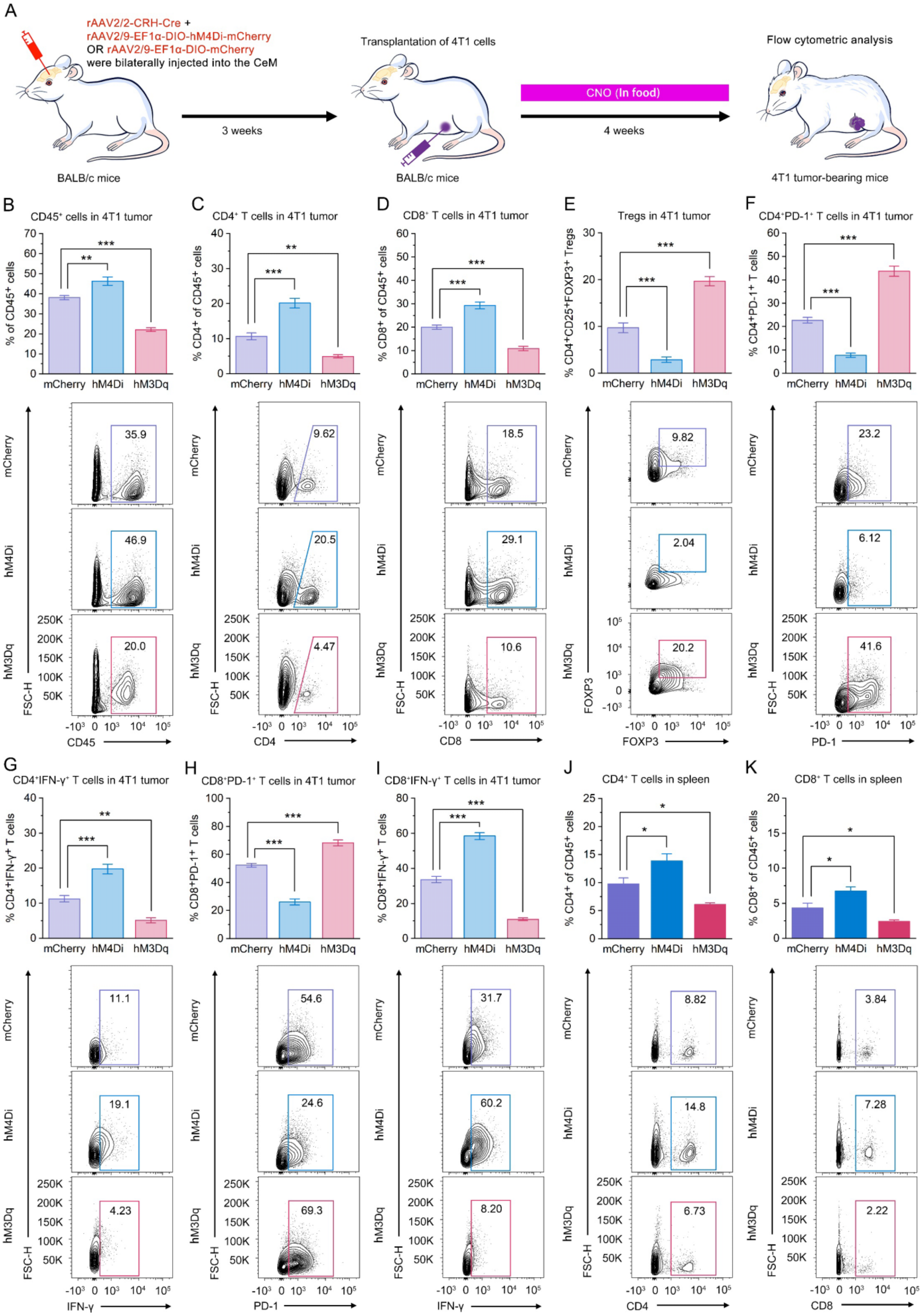
A
rAAV2/2-CRH-Cre +
rAAV2/9-EF1α-DIO-hM4Di-mCherry
OR rAAV2/9-EF1α-DIO-mCherry
were bilaterally injected into the CeM
BALB/c mice
3 weeks
Transplantation of 4T1 cells
BALB/c mice
CNO (In food)
4 weeks
Flow cytometric analysis
4T1 tumor-bearing mice
B
CD45⁺ cells in 4T1 tumor
% of CD45⁺ cells
C
CD4⁺ T cells in 4T1 tumor
% CD4⁺ of CD45⁺ cells
D
CD8⁺ T cells in 4T1 tumor
% CD8⁺ of CD45⁺ cells
E
Tregs in 4T1 tumor
% CD4⁺CD25⁺FOXP3⁺ Tregs
F
CD4⁺PD-1⁺ T cells in 4T1 tumor
% CD4⁺PD-1⁺ T cells
mCherry
hM4Di
hM3Dq
FSC-H
CD45
CD4
CD8
FOXP3
PD-1
35.9
46.9
20.0
9.62
20.5
4.47
18.5
29.1
10.6
9.82
2.04
20.2
23.2
6.12
41.6
G
CD4⁺IFN-γ⁺ T cells in 4T1 tumor
% CD4⁺IFN-γ⁺ T cells
H
CD8⁺PD-1⁺ T cells in 4T1 tumor
% CD8⁺PD-1⁺ T cells
I
CD8⁺IFN-γ⁺ T cells in 4T1 tumor
% CD8⁺IFN-γ⁺ T cells
J
CD4⁺ T cells in spleen
% CD4⁺ of CD45⁺ cells
K
CD8⁺ T cells in spleen
% CD8⁺ of CD45⁺ cells
IFN-γ
PD-1
11.1
19.1
4.23
54.6
24.6
69.3
31.7
60.2
8.20
8.82
14.8
6.73
3.84
7.28
2.22

Supplemental Figure 14. Chemogenetic manipulation of CeM^{CRH} neurons significantly affects antitumor immunity in 4T1 tumor-bearing mice. Related to Figures 5 and 6.

(A) Schematic of the experimental design.

(B) Representative flow cytometric plots and quantification of infiltrated CD45^{+} cells in 4T1 tumor of the mCherry, hM4Di and hM3Dq groups.

(C, D) Representative flow cytometric plots and quantification of infiltrated CD4^{+} T cells (C) and CD8^{+} T cells (D) in 4T1 tumor of the mCherry, hM4Di and hM3Dq groups.

(E) Representative flow cytometric plots and quantification of infiltrated Tregs (CD4^{+}CD25^{+}FOXP3^{+} T regulatory cells) in 4T1 tumor of the mCherry, hM4Di and hM3Dq groups.

(F, G) Representative flow cytometric plots and quantification of infiltrated CD4^{+}PD-1^{+} T cells (F) and CD4^{+}IFN-γ^{+} T cells (G) in 4T1 tumor of the mCherry, hM4Di and hM3Dq groups.

(H, I) Representative flow cytometric plots and quantification of infiltrated CD8^{+}PD-1^{+} T cells (H) and CD8^{+}IFN-γ^{+} T cells (I) in 4T1 tumor of the mCherry, hM4Di and hM3Dq groups.

(J, K) Representative flow cytometric plots and quantification of infiltrated CD4^{+} T cells (J) and CD8^{+} T cells (K) in spleen of the mCherry, hM4Di and hM3Dq groups.

n = 5 for each group. Data are presented as means ± SEM. $*P < 0.05$, $**P < 0.01$, $***P < 0.001$, one-way ANOVA followed by Tukey post-hoc test.

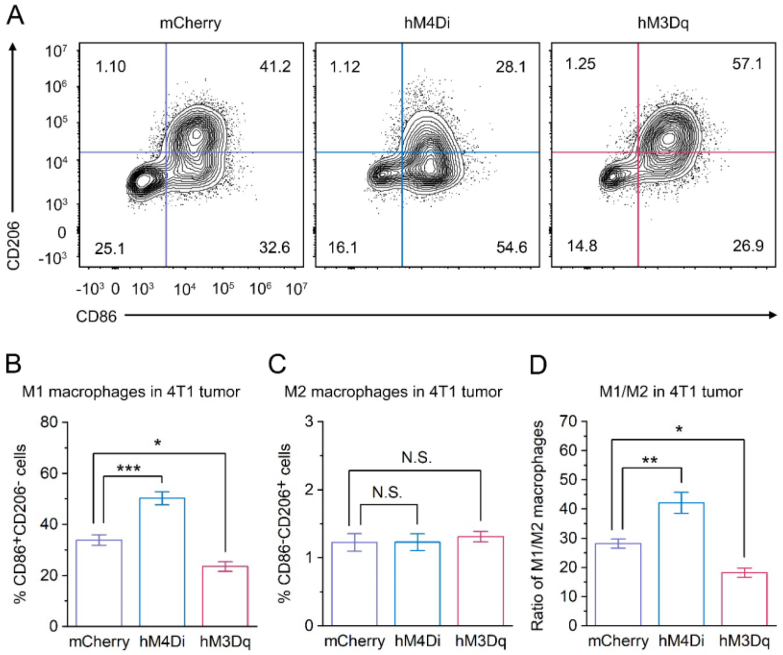

Supplemental Figure 15. Chemogenetic manipulation of CeM^{CRH} neurons significantly affects M1 macrophages in tumor from 4T1 tumor-bearing mice. Related to Figures 5 and 6.

(A) Representative flow cytometry analysis of macrophages stained with CD86 and CD206 in 4T1 tumor.

(B, C) Quantification of M1 macrophages population (CD11b^{+}F4/80^{+}CD86^{+}CD206$^{–}$) and M2 macrophages population (CD11b^{+}F4/80^{+}CD86$^{–}$CD206^{+}) in 4T1 tumor of the mCherry, hM4Di and hM3Dq groups.

(D) The ratio of M1/M2 macrophage in 4T1 tumor of the mCherry, hM4Di and hM3Dq groups.

n = 5 for each group. Data are presented as means ± SEM. N.S., not significant, *P < 0.05, **P < 0.01, ***P < 0.001, one-way ANOVA followed by Tukey post-hoc test.

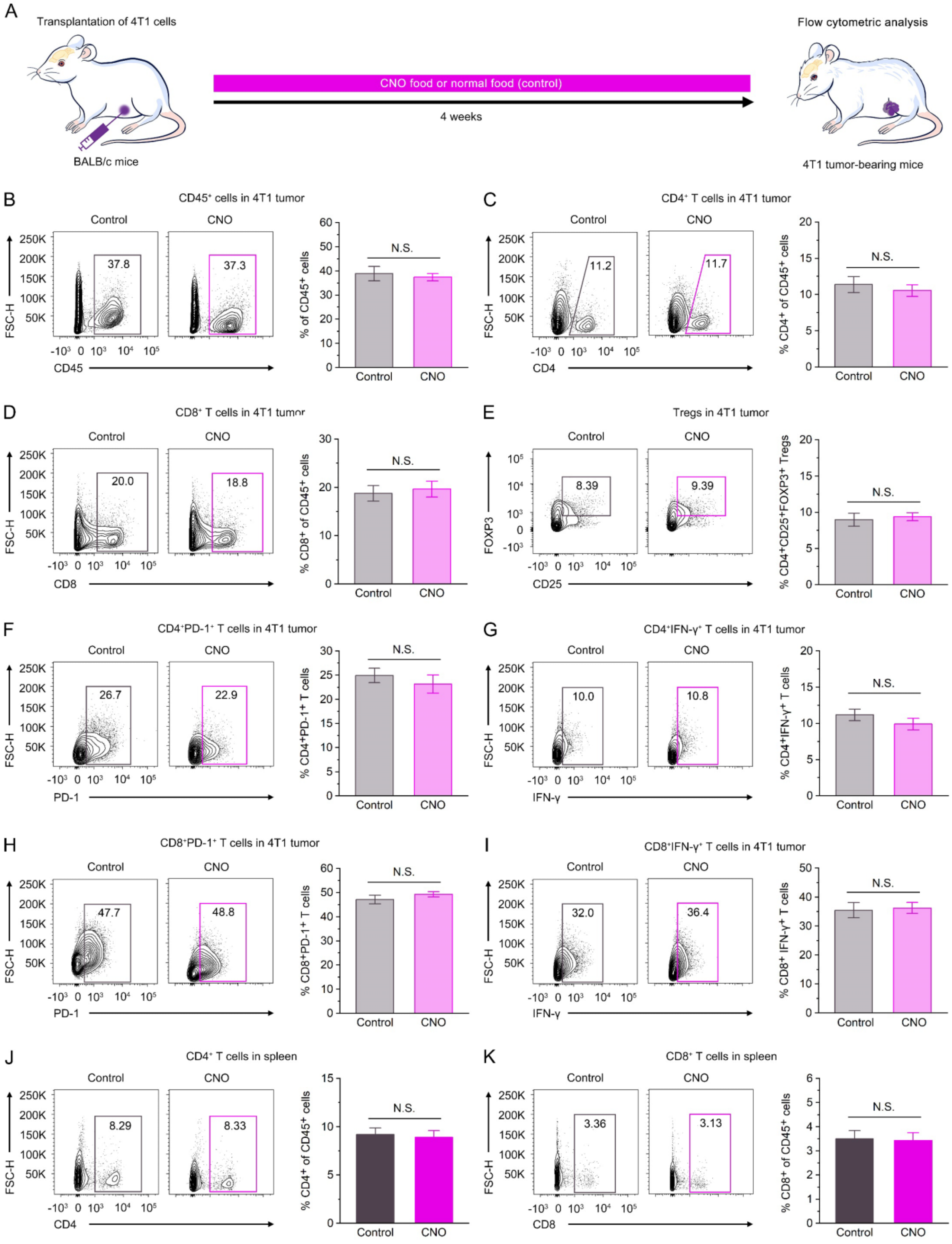

Supplemental Figure 16. The CNO has no significant effect on antitumor immunity in 4T1 tumor-bearing mice. Related to Figures 5 and 6.

(A) Schematic of the experimental design.

(B) Representative flow cytometric plots and quantification of infiltrated $CD45^+$ cells in 4T1 tumor of the control (normal food) and CNO groups.

(C, D) Representative flow cytometric plots and quantification of infiltrated $CD4^+$ T cells (C) and $CD8^+$ T cells (D) in 4T1 tumor of the control and CNO groups.

(E) Representative flow cytometric plots and quantification of infiltrated Tregs ($CD4^+CD25^+FOXP3^+$ T regulatory cells) in 4T1 tumor of the control and CNO groups.

(F, G) Representative flow cytometric plots and quantification of infiltrated $CD4^+PD\text{-}1^+$ T cells (F) and $CD4^+IFN\text{-}\gamma^+$ T cells (G) in 4T1 tumor of the control and CNO groups.

(H, I) Representative flow cytometric plots and quantification of infiltrated $CD8^+PD\text{-}1^+$ T cells (H) and $CD8^+IFN\text{-}\gamma^+$ T cells (I) in 4T1 tumor of the control and CNO groups.

(J, K) Representative flow cytometric plots and quantification of infiltrated $CD4^+$ T cells (J) and $CD8^+$ T cells (K) in spleen of the control and CNO groups.

n = 5 for each group. Data are presented as means ± SEM. *$P < 0.05$, **$P < 0.01$, ***$P < 0.001$, two-tailed unpaired Student’s t test.

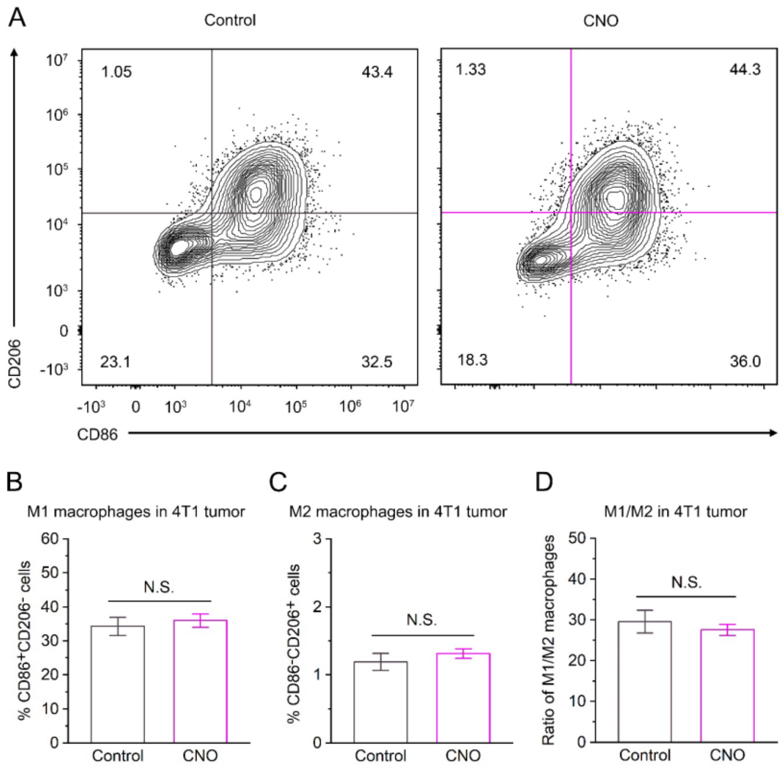

Supplemental Figure 17. The CNO has no significant effect on M1 macrophages and M2 macrophage in tumor from 4T1 tumor-bearing mice. Related to Figures 5 and 6.

(A) Representative flow cytometry analysis of macrophages stained with CD86 and CD206 in 4T1 tumor.

(B, C) Quantification of M1 macrophages population ($CD11b^{+}F4/80^{+}CD86^{+}CD206^{-}$) and M2 macrophages population ($CD11b^{+}F4/80^{+}CD86^{-}CD206^{+}$) in 4T1 tumor of the control and CNO groups.

(D) The ratio of M1/M2 macrophage in 4T1 tumor of the control and CNO groups.

n = 5 for each group. Data are presented as means ± SEM. N.S., not significant, two-tailed unpaired Student’s t test.

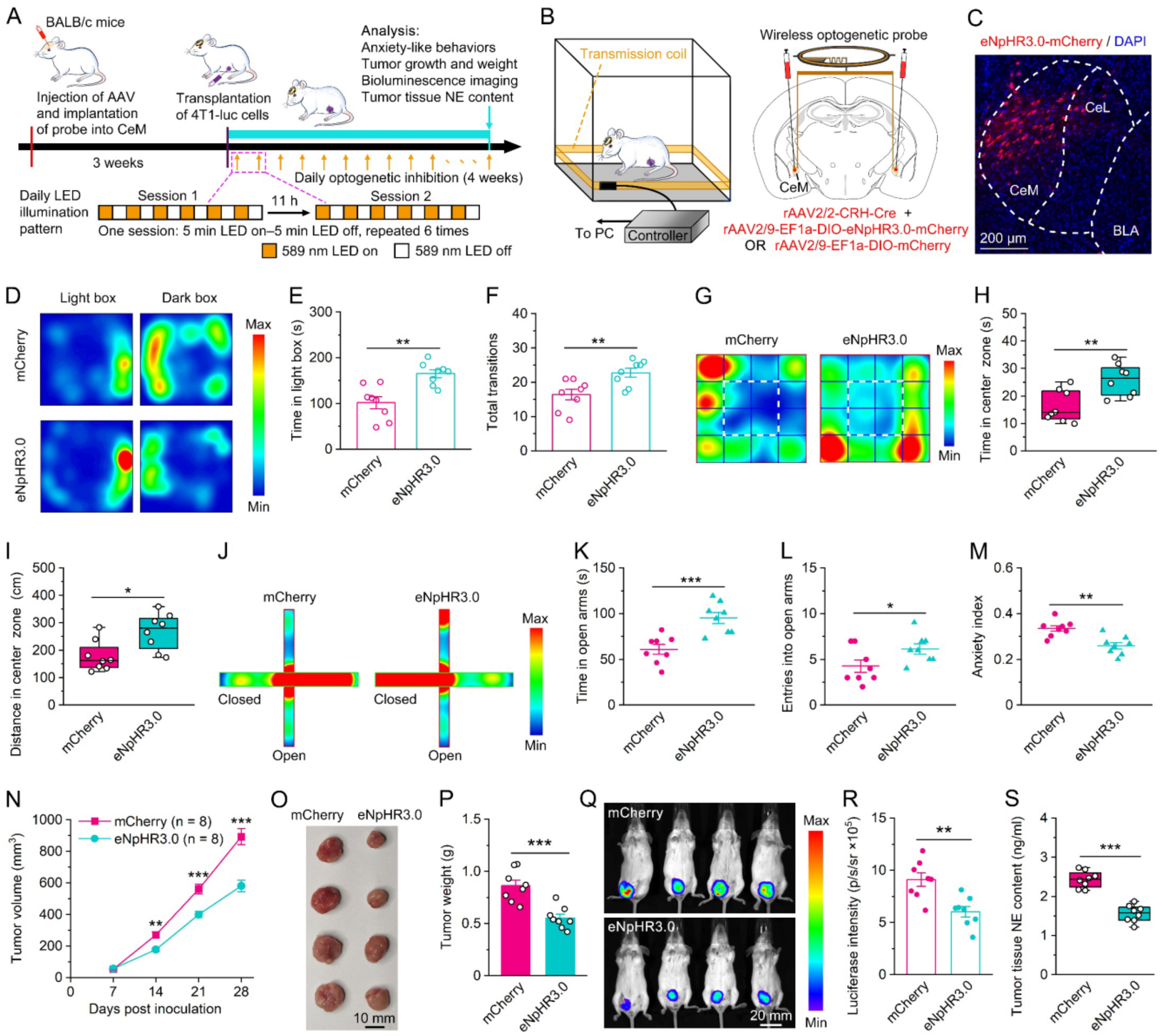

Supplemental Figure 18. Optogenetic inhibition of CeM^{CRH} neurons significantly attenuates both cancer-induced anxiety and 4T1 tumor progression.

(A) Schematic of the experimental design.

(B) Schematic showing bilateral injections of rAAV2/2-CRH-Cre together with rAAV2/9-EF1α-DIO-eNpHR3.0-mCherry or rAAV2/9-EF1α-DIO-mCherry into the CeM, and wireless optogenetic inhibition.

(C) Representative images showing eNpHR3.0-mCherry expression in CeM^{CRH} neurons.

(D–F) In the light–dark box test, representative heatmaps (D), and quantitative summary of time spent in light box (E) and the total number of transitions (F) in the mCherry (n = 8) and eNpHR3.0 (n = 8) groups.

(G–I) In the open field test, representative heatmaps (G), and quantitative summary of the time spent

in the center zone (H) and the distance traveled in the center zone (I) in the mCherry (n = 8) and eNpHR3.0 (n = 8) groups.

(J–M) In the elevated plus maze test, representative heatmaps (J), and quantitative summary of the time spent in the open arms (K), entries into open arms (L) and the anxiety index (M) in the mCherry (n = 8) and eNpHR3.0 (n = 8) groups.

(N) Optogenetic inhibition of CeM^{CRH} neurons significantly slows 4T1 tumor growth.

(O) Representative tumors dissected from the two groups.

(P) Optogenetic inhibition of CeM^{CRH} neurons significantly reduces 4T1 tumor weight.

(Q) Representative bioluminescence images of the two groups.

(R, S) Chemogenetic inhibition of the CeM^{CRH} neurons significantly reduces the luciferase intensity of 4T1 tumor (R) and tumor tissue NE content (S; P, R, S: mCherry, n = 8, eNpHR3.0, n = 8).

Data are presented as means ± SEM, except in box plots (H, I, S), in which centerlines indicate medians, box edges represent the first and third quartiles and whiskers denote minimal and maximal values. *P < 0.05, **P < 0.01, ***P < 0.001, two-way repeated-measures ANOVA followed by separate one-way ANOVA (N), two-tailed unpaired Student’s t test (E, F, H, I, K–M, P, R, S).

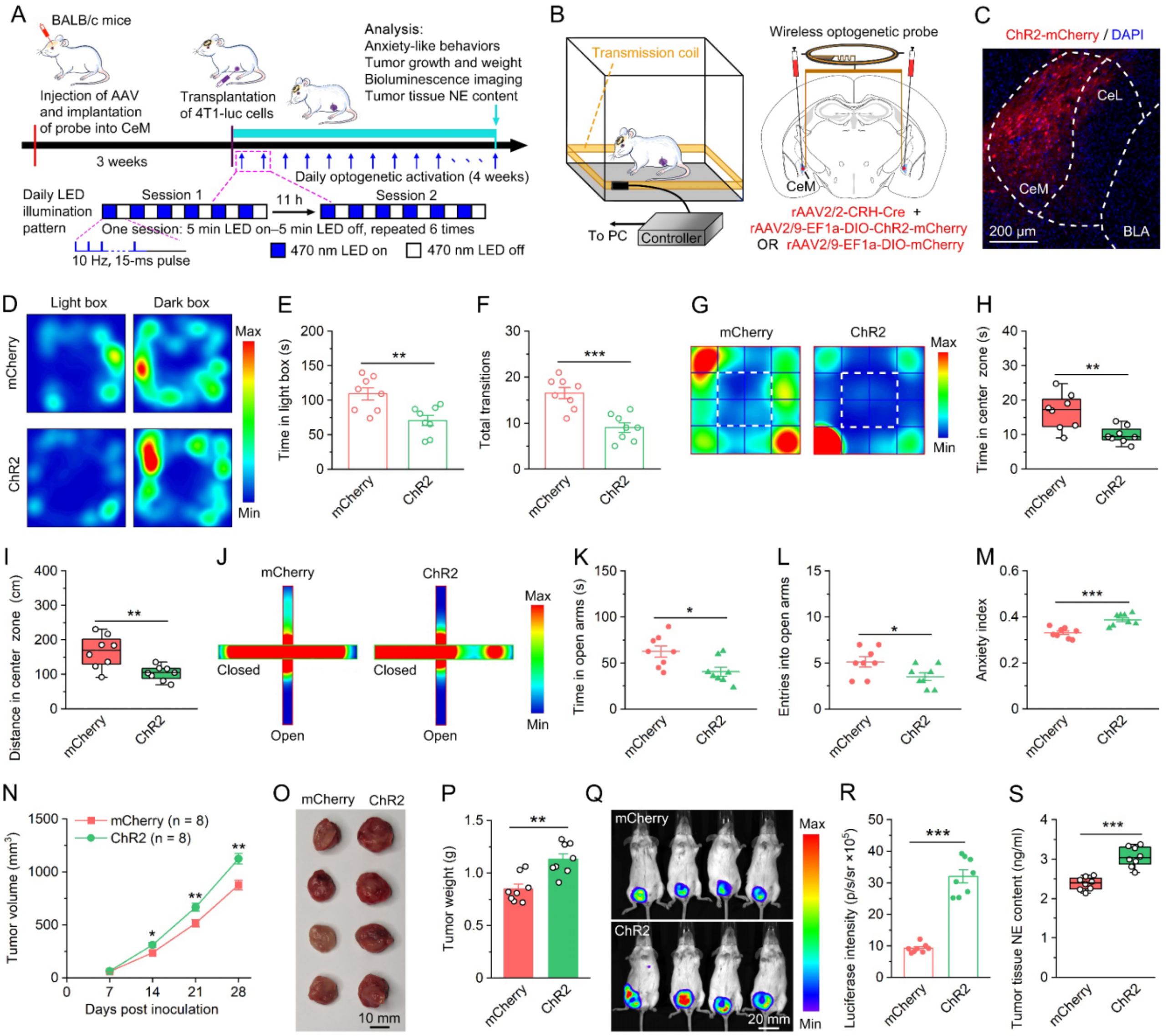

Supplemental Figure 19. Optogenetic activation of the CeM^{CRH} neurons significantly increases cancer-induced anxiety and accelerates 4T1 tumor progression.

(A) Schematic illustration of the experimental design.

(B) Schematic showing bilateral injections of rAAV2/2-CRH-Cre together with rAAV2/9-EF1α-DIO-ChR2-mCherry or rAAV2/9-EF1α-DIO-mCherry into the CeM, and wireless optogenetic activation.

(C) Representative images showing ChR2-mCherry expression in the CeM.

(D–F) In the light–dark box test, representative heatmaps (D), and quantitative summary of time spent in light box (E) and the total number of transitions (F) in the mCherry (n = 8) and ChR2 (n = 8) groups.

(G–I) In the open field test, representative heatmaps (G), and quantitative summary of the time spent in the center zone (H) and the distance traveled in the center zone (I) in the mCherry (n = 8) and ChR2 (n = 8) groups.

(J–M) In the elevated plus maze test, representative heatmaps (J), and quantitative summary of the time spent in the open arms (K), entries into open arms (L) and the anxiety index (M) in the mCherry (n = 8) and ChR2 (n = 8) groups.

(N) Optogenetic activation of CeM^{CRH} neurons significantly accelerates 4T1 tumor growth.

(O) Representative tumors dissected from the two groups.

(P) Optogenetic activation of the CeM^{CRH} neurons significantly increases 4T1 tumor weight.

(Q) Representative bioluminescence images of the two groups.

(R, S) Optogenetic activation of the CeM^{CRH} neurons significantly increases the luciferase intensity of the 4T1 tumor (R) and tumor tissue NE content (S; P, R, S: mCherry, n = 8, ChR2, n = 8).

Data are presented as means ± SEM, except in box plots (H, I, S), in which centerlines indicate medians, box edges represent the first and third quartiles and whiskers denote minimal and maximal values. *P < 0.05, **P < 0.01, ***P < 0.001, two-way repeated-measures ANOVA followed by separate one-way ANOVA (N), two-tailed unpaired Student's t test (E, F, H, I, K–M, P, R, S).

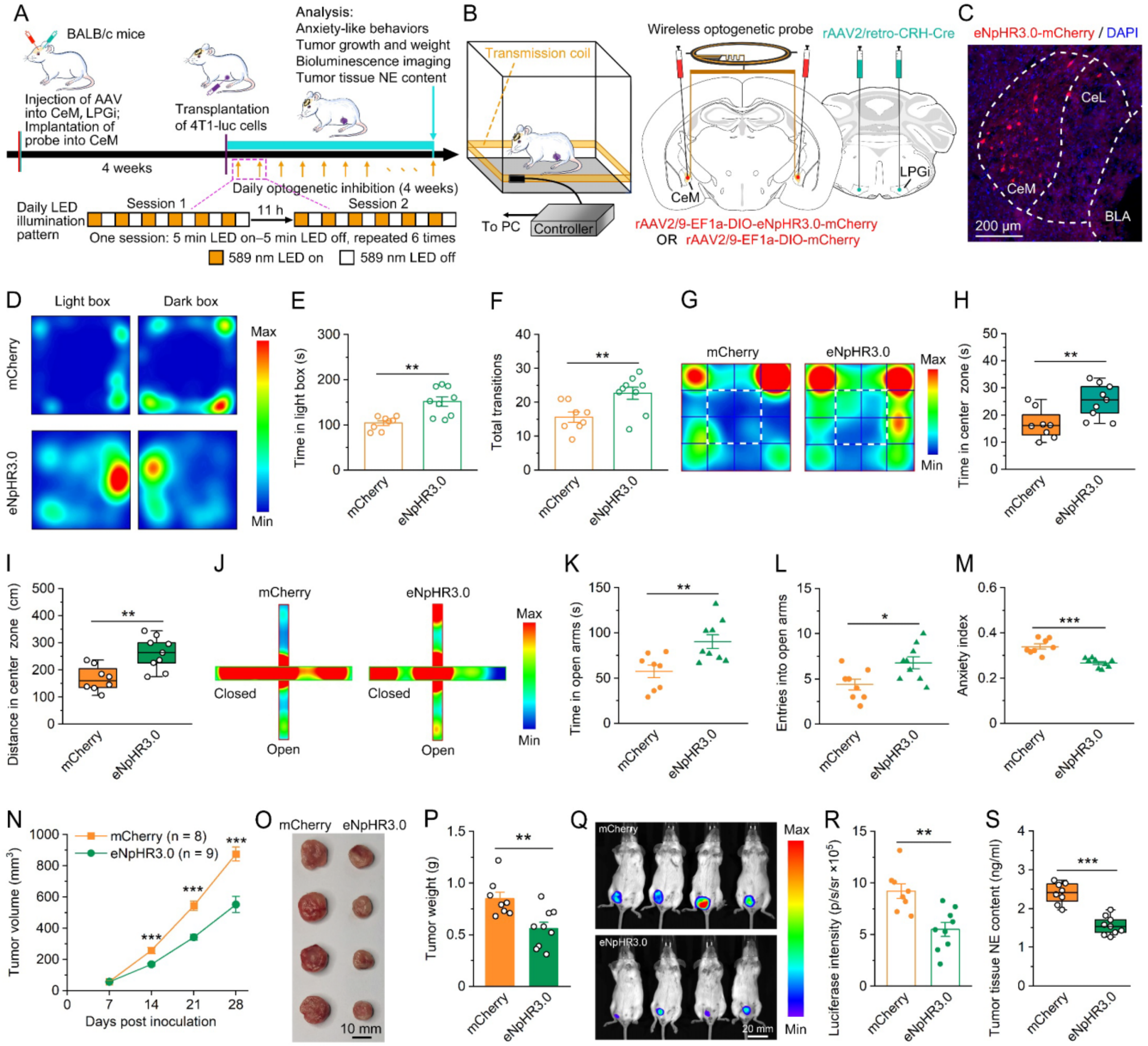

Supplemental Figure 20. Optogenetic inhibition of the CeM^{CRH}→LPGi circuit significantly attenuates both cancer-induced anxiety and 4T1 tumor progression.

(A) Schematic of the experimental design.

(B) Schematic showing bilateral injections of rAAV2/retro-CRH-Cre into the LPGi, and of rAAV2/9-EF1α-DIO-eNpHR3.0-mCherry or rAAV2/9-EF1α-DIO-mCherry into the CeM, and wireless optogenetic inhibition.

(C) Representative images showing eNpHR3.0-mCherry expression in CeM^{CRH} neurons.

(D–F) In the light–dark box test, representative heatmaps (D), and quantitative summary of time spent in light box (E) and the total number of transitions (F) in the mCherry (n = 8) and eNpHR3.0 (n = 9) groups.

(G–I) In the open field test, representative heatmaps (G), and quantitative summary of the time spent in the center zone (H) and the distance traveled in the center zone (I) in the mCherry (n = 8) and eNpHR3.0 (n = 9) groups.

(J–M) In the elevated plus maze test, representative heatmaps (J), and quantitative summary of the time spent in the open arms (K), entries into open arms (L) and the anxiety index (M) in the mCherry (n = 8) and eNpHR3.0 (n = 9) groups.

(N) Optogenetic inhibition of the CeM^{CRH}→LPGi circuit significantly slows 4T1 tumor growth.

(O) Representative tumors dissected from the two groups.

(P) Optogenetic inhibition of the CeM^{CRH}→LPGi circuit significantly reduces 4T1 tumor weight.

(Q) Representative bioluminescence images of the two groups.

(R, S) Chemogenetic inhibition of the CeM^{CRH}→LPGi circuit significantly reduces the luciferase intensity of 4T1 tumor (R) and tumor tissue NE content (S; P, R, S: mCherry, n = 8, eNpHR3.0, n = 9). Data are presented as means ± SEM, except in box plots (H, I, S), in which centerlines indicate medians, box edges represent the first and third quartiles and whiskers denote minimal and maximal values. $^{*}P < 0.05$, $^{**}P < 0.01$, $^{***}P < 0.001$, two-way repeated-measures ANOVA followed by separate one-way ANOVA (N), two-tailed unpaired Student’s t test (E, F, H, I, K–M, P, R, S).

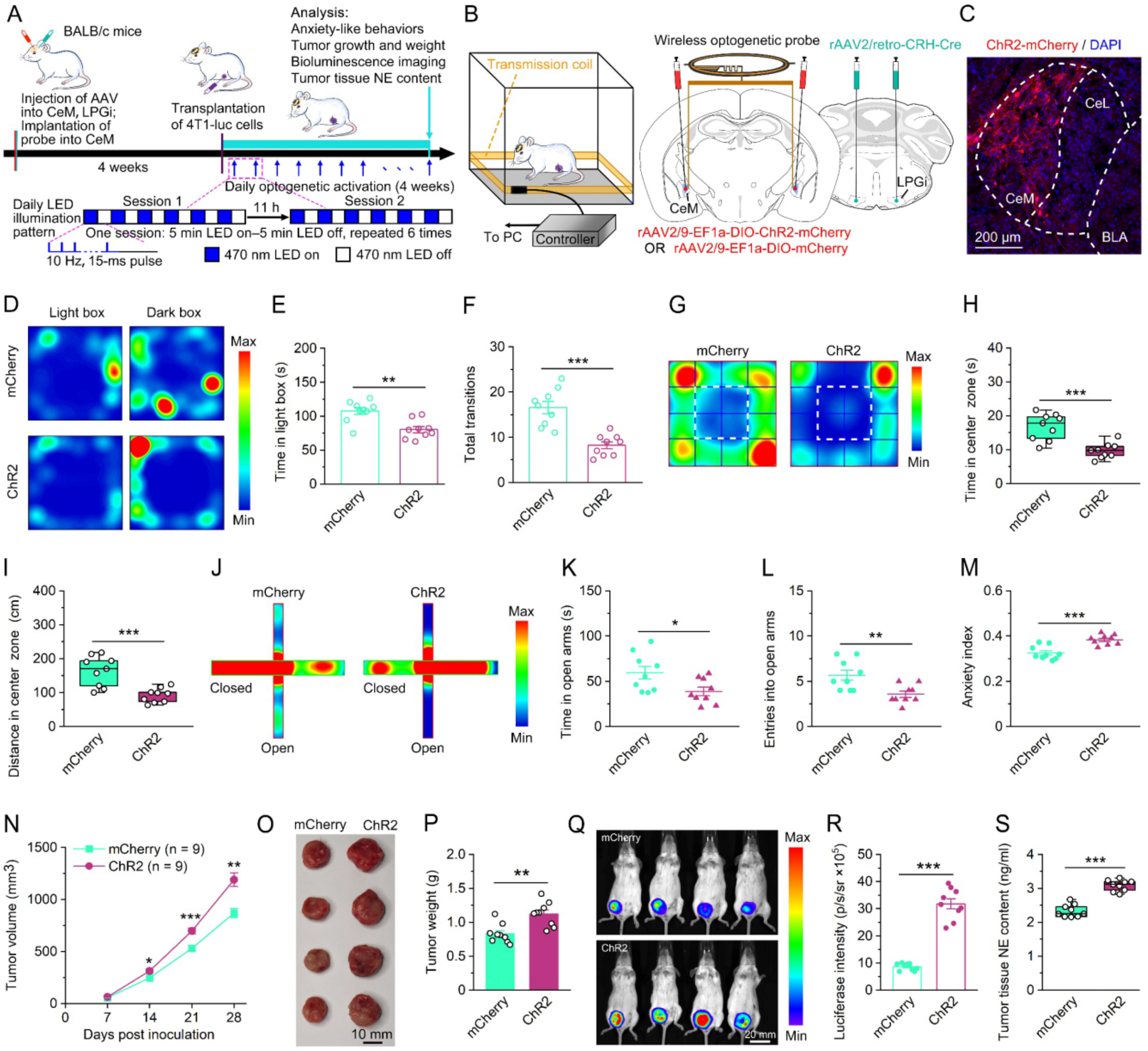

Figure 21. Optogenetic activation of the CeM^{CRH}→LPGi circuit significantly increases cancer-induced anxiety and accelerates 4T1 tumor progression.

(A) Schematic illustration of the experimental design.

(B) Schematic showing bilateral injections of rAAV2/retro-CRH-Cre into the LPGi, and of rAAV2/9-EF1α-DIO-ChR2-mCherry or rAAV2/9-EF1α-DIO-mCherry into the CeM, and wireless optogenetic activation.

(C) Representative images showing ChR2-mCherry expression in the CeM.

(D–F) In the light–dark box test, representative heatmaps (D), and quantitative summary of time spent in light box (E) and the total number of transitions (F) in the mCherry (n = 9) and ChR2 (n = 9) groups.

(G–I) In the open field test, representative heatmaps (G), and quantitative summary of the time spent

in the center zone (H) and the distance traveled in the center zone (I) in the mCherry (n = 9) and ChR2 (n = 9) groups.

(J–M) In the elevated plus maze test, representative heatmaps (J), and quantitative summary of the time spent in the open arms (K), entries into open arms (L) and the anxiety index (M) in mCherry (n = 9) and ChR2 (n = 9) groups.

(N) Optogenetic activation of the CeM^{CRH}→LPGi circuit significantly accelerates 4T1 tumor growth.

(O) Representative tumors dissected from the two groups.

(P) Optogenetic activation of the CeM^{CRH}→LPGi circuit significantly increases 4T1 tumor weight.

(Q) Representative bioluminescence images of the two groups.

(R, S) Optogenetic activation of the CeM^{CRH}→LPGi circuit significantly increases the luciferase intensity of 4T1 tumor (R) and tumor tissue NE content (S; P, R, S: mCherry, n = 9, ChR2, n = 9).

Data are presented as means ± SEM, except in box plots (H, I, S), in which centerlines indicate medians, box edges represent the first and third quartiles and whiskers denote minimal and maximal values. *$P < 0.05$, **$P < 0.01$, ***$P < 0.001$, two-way repeated-measures ANOVA followed by separate one-way ANOVA (N), two-tailed unpaired Student’s t test (E, F, H, I, K–M, P, R, S).

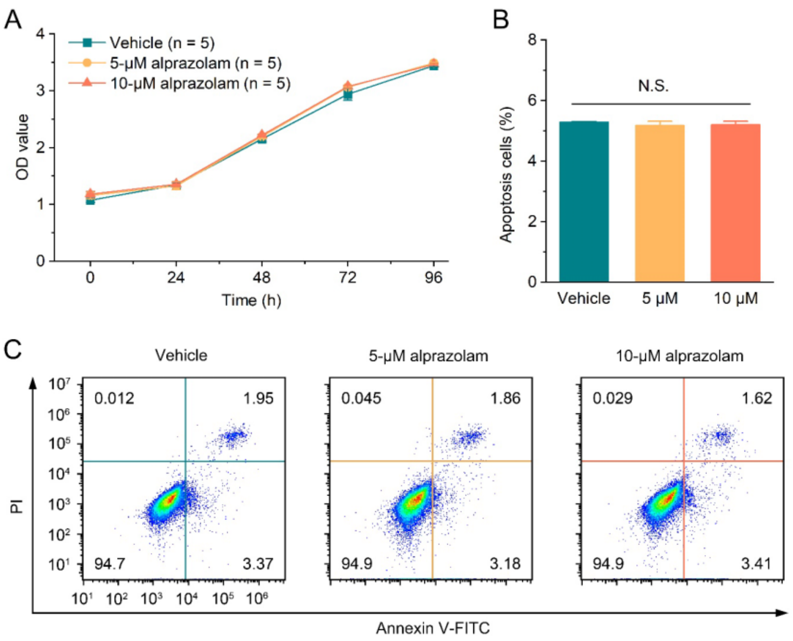

Supplemental Figure 22. Alprazolam has no significant effect on proliferation and apoptosis of 4T1 breast cancer cells in vitro. Related to Figure 10.

(A) Alprazolam has no significant effect on the proliferation of 4T1 cells in vitro. CCK8 assay was performed to determine the cell growth of 4T1 cells at consecutive time points with alprazolam.

(B and C) Alprazolam has no significant effect on the apoptosis of 4T1 cells in vitro. After 48 hours of alprazolam treatment, the apoptosis rate of 4T1 cells was determined by flow cytometry analysis (n = 4 for each group).

N.S., not significant, two-way repeated-measures ANOVA followed by separate one-way ANOVA (A), one-way ANOVA followed by Tukey post hoc test (B).

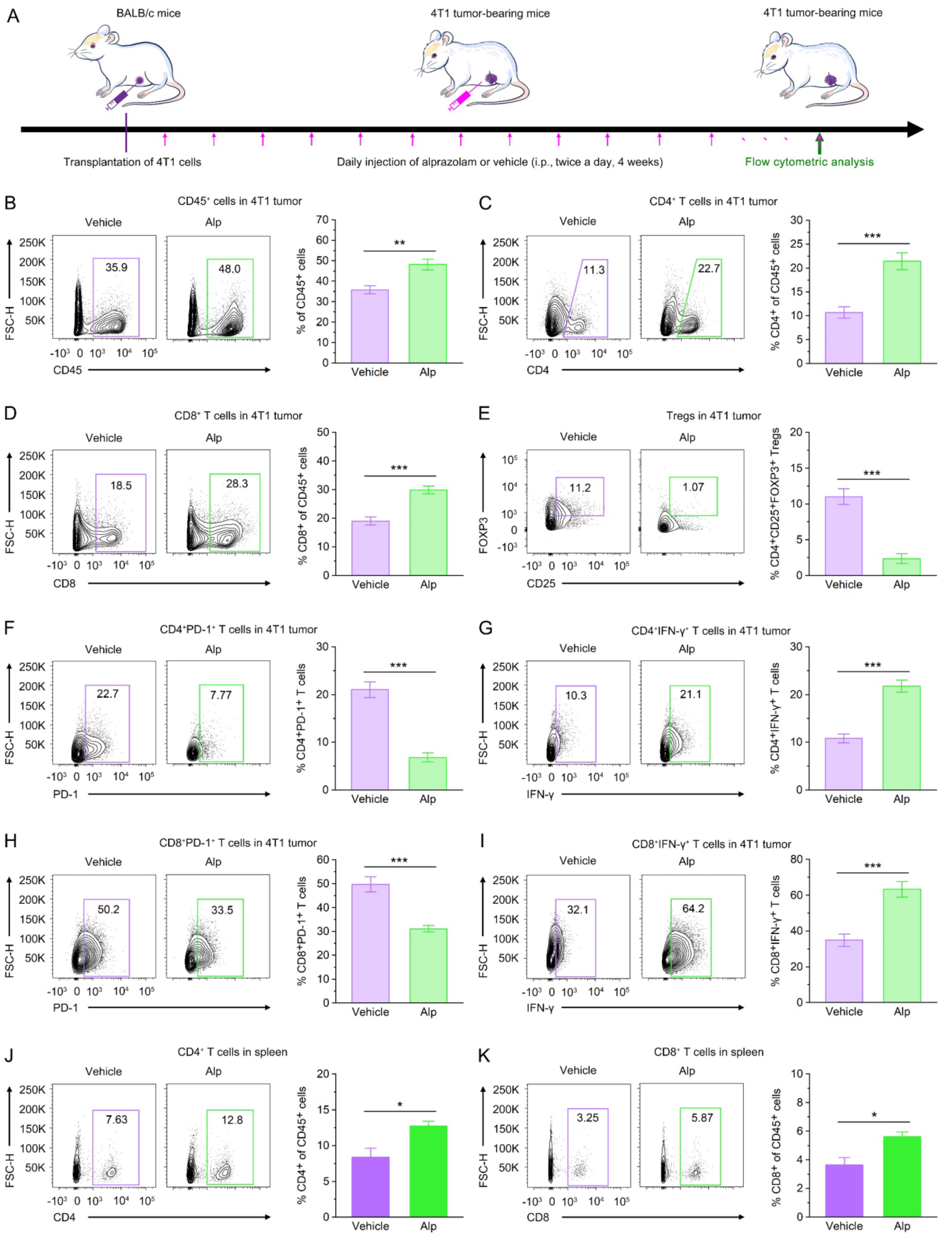

Supplemental Figure 23. Alprazolam treatment significantly enhances the antitumor immunity in 4T1 tumor-bearing mice. Related to Figure 10.

(A) Schematic of the experimental design.

(B) Representative flow cytometric plots and quantification of infiltrated $CD45^+$ cells in 4T1 tumor of the Vehicle and Alp groups.

(C, D) Representative flow cytometric plots and quantification of infiltrated $CD4^+$ T cells (C) and $CD8^+$ T cells (D) in 4T1 tumor of the Vehicle and Alp groups.

(E) Representative flow cytometric plots and quantification of infiltrated Tregs ($CD4^+CD25^+FOXP3^+$ T regulatory cells) in 4T1 tumor of the Vehicle and Alp groups.

(F, G) Representative flow cytometric plots and quantification of infiltrated $CD4^+PD\text{-}1^+$ T cells (F) and $CD4^+IFN\text{-}\gamma^+$ T cells (G) in 4T1 tumor of the Vehicle and Alp groups.

(H, I) Representative flow cytometric plots and quantification of infiltrated $CD8^+PD\text{-}1^+$ T cells (H) and $CD8^+IFN\text{-}\gamma^+$ T cells (I) in 4T1 tumor of the Vehicle and Alp group.

(J, K) Representative flow cytometric plots and quantification of infiltrated $CD4^+$ T cells (J) and $CD8^+$ T cells (K) in spleen of the Vehicle and Alp groups.

n = 5 for each group. Data are presented as means ± SEM. *$P < 0.05$, **$P < 0.01$, ***$P < 0.001$, two-tailed unpaired Student’s t test.

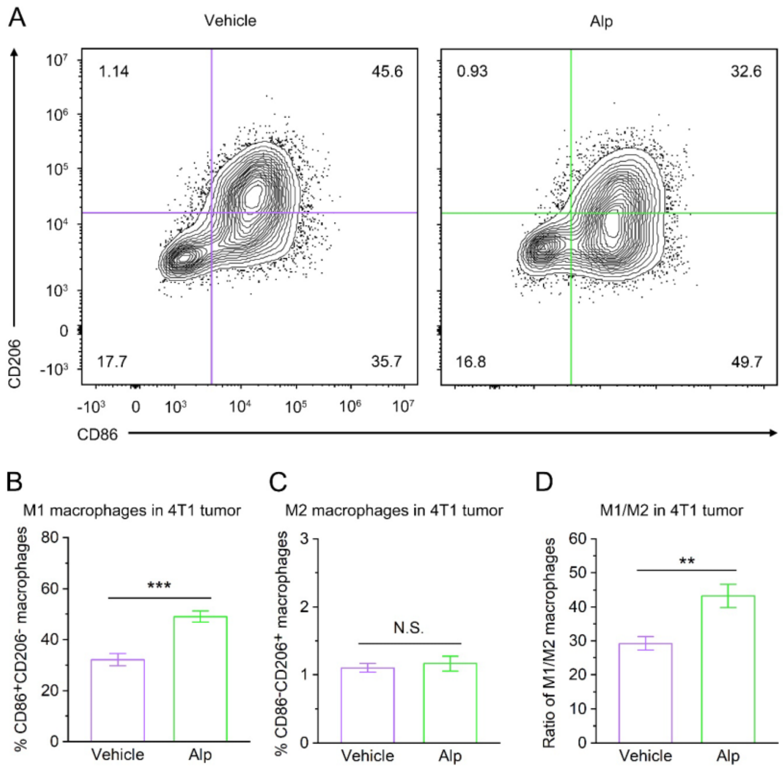

Supplemental Figure 24. Alprazolam treatment significantly increases M1 macrophages in tumor from 4T1 tumor-bearing mice. Related to Figure 10.

(A) Representative flow cytometry analysis of macrophages stained with CD86 and CD206 in 4T1 tumor.

(B, C) Quantification of M1 macrophages population ($CD11b^{+}F4/80^{+}CD86^{+}CD206^{-}$) and M2 macrophages population ($CD11b^{+}F4/80^{+}CD86^{-}CD206^{+}$) in 4T1 tumor of the Vehicle and Alp groups.

(D) The ratio of M1/M2 macrophage in 4T1 tumor of the Vehicle and Alp groups.

n = 5 for each group. Data are presented as means ± SEM. N.S., not significant, $^{**}P < 0.01$, $^{***}P < 0.001$, two-tailed unpaired Student's t test.

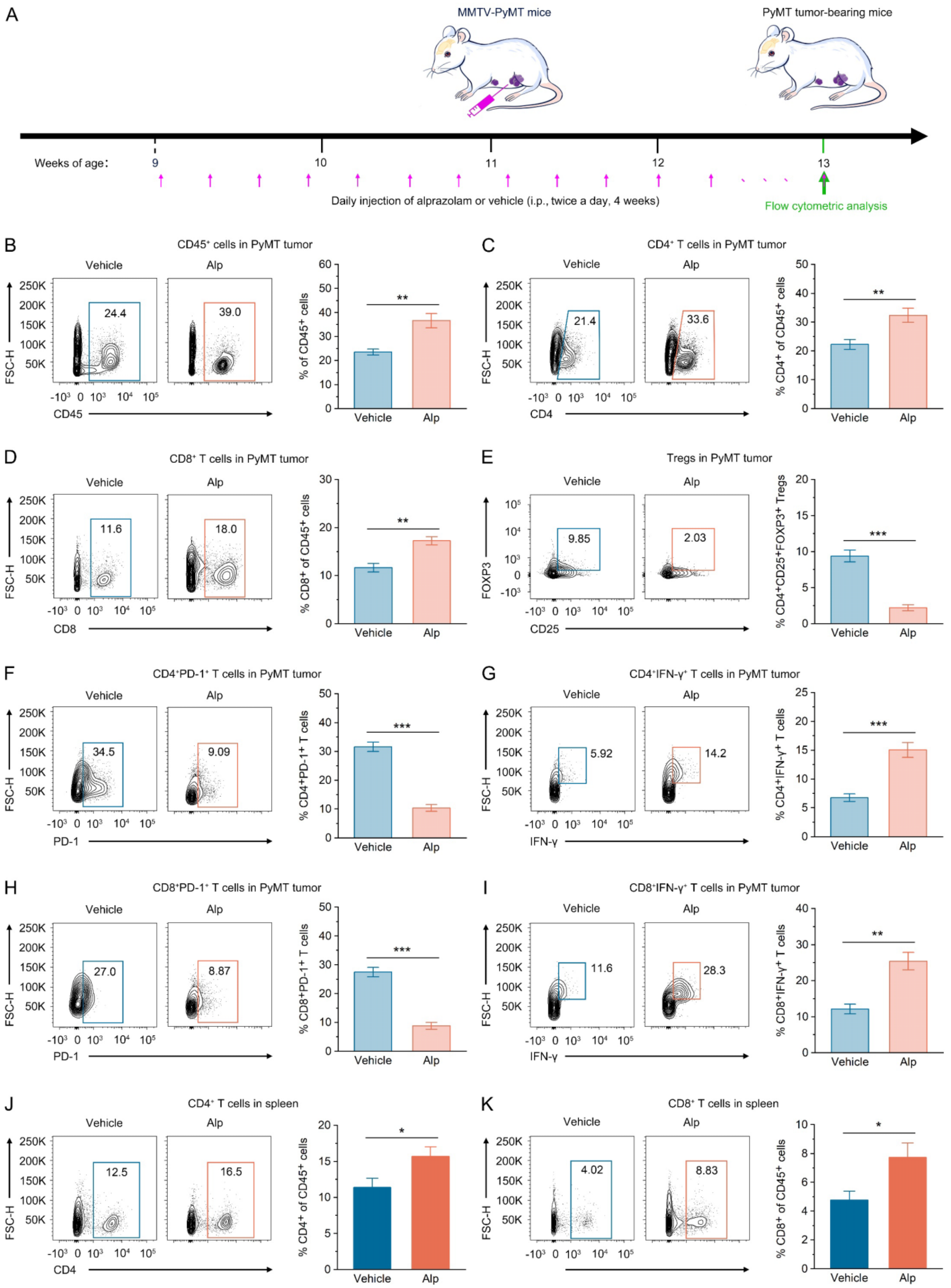

Supplemental Figure 25. Alprazolam treatment significantly enhances the antitumor immunity

in PyMT tumor-bearing mice. Related to Figure 11.

(A) Schematic of the experimental design.

(B) Representative flow cytometric plots and quantification of infiltrated $CD45^+$ cells in PyMT tumor of the Vehicle and Alp groups.

(C, D) Representative flow cytometric plots and quantification of infiltrated $CD4^+$ T cells (C) and $CD8^+$ T cells (D) in PyMT tumor of the Vehicle and Alp groups.

(E) Representative flow cytometric plots and quantification of infiltrated Tregs ($CD4^+CD25^+FOXP3^+$ T regulatory cells) in PyMT tumor of the Vehicle and Alp groups.

(F, G) Representative flow cytometric plots and quantification of infiltrated $CD4^+PD\text{-}1^+$ T cells (F) and $CD4^+IFN\text{-}\gamma^+$ T cells (G) in PyMT tumor of the Vehicle and Alp groups.

(H, I) Representative flow cytometric plots and quantification of infiltrated $CD8^+PD\text{-}1^+$ T cells (H) and $CD8^+IFN\text{-}\gamma^+$ T cells (I) in PyMT tumor of the Vehicle and Alp groups.

(J, K) Representative flow cytometric plots and quantification of infiltrated $CD4^+$ T cells (J) and $CD8^+$ T cells (K) in spleen of MMTV-PyMT mice in the Vehicle and Alp groups.

n = 5 for each group. Data are presented as means ± SEM. *P < 0.05, **P < 0.01, ***P < 0.001, two-tailed unpaired Student’s t test.

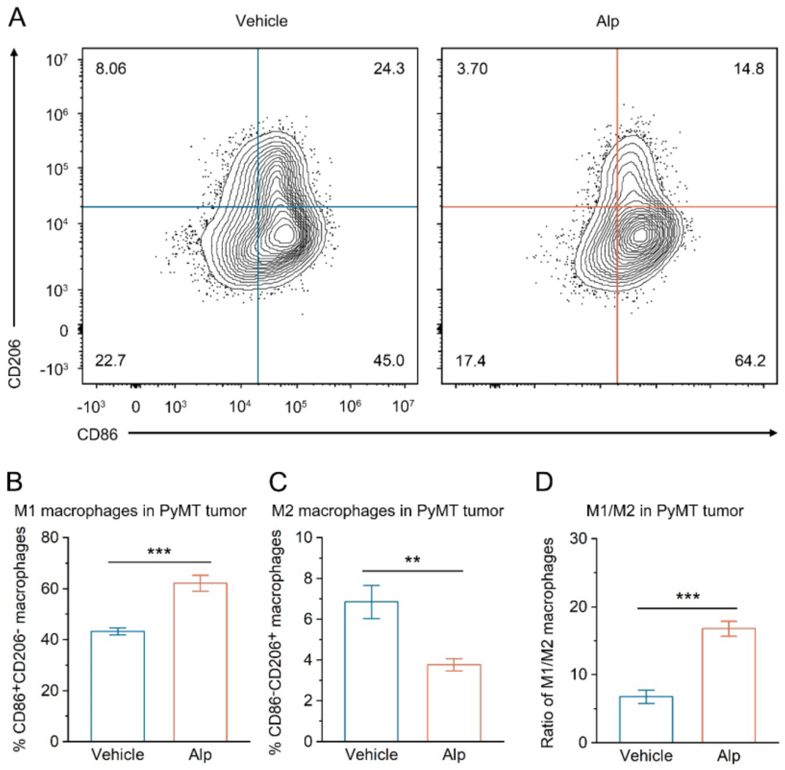

Supplemental Figure 26. Alprazolam treatment significantly increases M1 macrophages and decreases M2 macrophages in tumor from PyMT tumor-bearing mice. Related to Figure 11.

(A) Representative flow cytometry analysis of macrophages stained with CD86 and CD206 in PyMT tumor.

(B, C) Quantification of M1 macrophages population ($CD11b^{+}F4/80^{+}CD86^{+}CD206^{-}$) and M2 macrophages population ($CD11b^{+}F4/80^{+}CD86^{-}CD206^{+}$) in PyMT tumor of the Vehicle and Alp groups.

(D) The ratio of M1/M2 macrophage in PyMT tumor of the Vehicle and Alp groups.

n = 5 for each group. Data are presented as means ± SEM. $^{**}P < 0.01$, $^{***}P < 0.001$, two-tailed unpaired Student’s t test.

Supplemental Table 1. Antibodies information

Primary antibodies	Source	Catalog No.	Concentration
Rat anti-c-Fos	Synaptic Systems	226017	1:500
Rabbit anti-c-Fos	Synaptic Systems	226003	1:500
Rabbit anti-Tyrosine Hydroxylase	Millipore	AB152	1:1000
Rabbit anti-Neurofilament-L	Cell Signaling Technology	2837	1:1000
Mouse anti-Neurofilament-L	Cell Signaling Technology	2835	1:1000
Mouse anti-CRF (CRH)	Santa Cruz	sc-293187	1:100
Rabbit anti-CRF (CRH)	Abcam	ab272391	1:100
Rabbit anti-Ki67	Cell Signaling Technology	9129	1:1000

Secondary antibodies	Source	Catalog No.	Concentration
Goat anti-Mouse, Alexa Fluor™ Plus 488	Invitrogen	A32723	1:1000
Goat anti-Mouse, Alexa Fluor™ 555	Invitrogen	A-21424	1:1000
Goat anti-Rabbit, Alexa Fluor® 488	Cell Signaling Technology	4412	1:1000
Goat anti-Rabbit, Alexa Fluor™ 488	Invitrogen	A-11034	1:1000
Goat anti-Rabbit, Alexa Fluor™ Plus 555	Invitrogen	A32732	1:1000
Goat anti-Rat, Alexa Fluor™ 488	Invitrogen	A-11006	1:1000

Supplemental References:

1. Jiang S, et al. Itch-specific neurons in the ventrolateral orbital cortex selectively modulate the itch processing. *Sci Adv.* 2022;8(30):eabn4408.
2. Wu GY, et al. Medial Prefrontal Cortex-Pontine Nuclei Projections Modulate Suboptimal Cue-Induced Associative Motor Learning. *Cereb Cortex.* 2018;28(3):880-893.
